# Nanocomposites from Au‐Doped Vinylogous Urethane Vitrimers Based on Different Block Copolymers and Their Recyclability in Combination with Plasmonic Heating

**DOI:** 10.1002/marc.202401027

**Published:** 2025-03-06

**Authors:** Patrick Schütz, Siraphat Weerathaworn, Clas Jürgensen, Birgit Hankiewicz, Volker Abetz

**Affiliations:** ^1^ Institute of Physical Chemistry University of Hamburg Grindelallee 117 20146 Hamburg Germany; ^2^ Institute of Membrane Research Helmholtz‐Zentrum Hereon Max‐Planck‐Straße 1 21502 Geesthacht Germany

**Keywords:** gold nanoparticles, ligand exchange, nanocomposites, plasmonic heating, recyclability, self‐healing, vinylogous urethane vitrimers

## Abstract

The combination of gold nanoparticles (Au‐NPs) and block copolymer (BCP)‐based vinylogous urethane vitrimers leads to advanced nanocomposites where the thermal, mechanical, and thermo‐mechanical properties are enhanced without interfering with the formation of vinylogous urethane groups and the transamination in the dynamic polymer network. Photoiniferter reversible addition‐fragmentation chain transfer polymerization (photoRAFT) and inverse Turkevich synthesis are used in this work to fabricate the desired BCPs and spherical Au‐NPs. The key feature of this synthesis is the integration of Au‐NPs into the polymer matrix as fixed parts of the hybrid network, ensuring full recyclability. A wide range of properties can be tuned by variations of gold content, monomers, and BCP architecture. After ligand exchange, network formation, and reprocessing through heat compression, the unique optical properties of Au‐NPs are retained, allowing plasmonic heating to trigger the transamination exchange reaction within the materials. As a result, the Au‐doped vitrimers can self‐heal and exhibit shape‐memory shortly after exposure to not only heat but also light. This incorporation of Au‐NPs into vitrimers could provide a versatile platform for the development of hybrid materials offering potential applications in coatings, sensors, electronic devices, etc.

## Graphical abstract

The incorporation of spherical gold nanoparticles as fixed parts in block copolymer‐based vinylogous urethane vitrimer networks results in improved thermal, mechanical, and thermo‐mechanical properties of the nanocomposites while maintaining the nanoparticles’ unique optical properties. The use of plasmonic heating enables the utilization of not only heat but also light to induce self‐healing and shape‐memory of the composites.

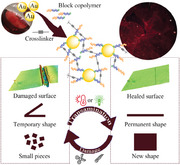

## 1 Introduction

Vitrimers are polymeric materials with characteristic thermo‐mechanical properties, grouped as associative covalent adaptable networks (CANs). This concept was first introduced by Leibler and coworkers in 2011.^[^ ^1^ ^]^ These materials contain dynamic exchange crosslinks that can be thermally activated at high temperatures, resulting in material flow. As the temperature drops, the exchange reaction and the network flow slow down until the material flow freezes or vitrifies, resulting in a permanent network.^[^ ^2^ ^]^ Transamination of vinylogous urethane vitrimers refers to a chemical reaction in which the amino groups in vinylogous urethanes are rearranged by a dynamic covalent exchange with free amino groups. This thermo‐reversible process is the key to the ability of vitrimers to retain their properties after self‐healing, reprocessing, and recycling.^[^ ^2^ ^]^

In BCPs two or more chemically different polymer blocks are covalently bonded. They have unique properties due to the distinct nature of the repeating units and the tendency to microphase into well‐defined morphologies, depending on segmental segregation strength and the relative amount of the different blocks.^[^ ^3^ ^]^ Microphase‐separated BCP materials have different thermal and mechanical properties compared to the corresponding random copolymers.^[^ ^4^ ^]^ In previous studies, linear BCPs have been introduced as nanostructured building blocks for the formation of vinylogous urethane vitrimers, capable of undergoing transamination as a dynamic exchange reaction.^[^ ^5^, ^6^ ^]^

Photoiniferter reversible addition‐fragmentation chain transfer (photoRAFT) polymerization is a type of controlled radical polymerization technique where a RAFT agent acts as a photoinitiator, transfer agent, and terminator.^[^ ^7^ ^]^ This technique allows good control of the molecular weight, dispersity, and architecture of polymers and BCPs.^[^ ^8^ ^]^

Fillers such as carbon nanotubes, graphene, silica, or aluminum oxide have previously been used in vitrimers to enhance their mechanical, viscoelastic, and/or thermal properties.^[^ ^9^ ^]^ Besides improving the properties of the materials, fillers can also be used to add new features to the materials, which have been demonstrated, for example, by the addition of carbon black or graphene leading to electrical conductivity or spiropyran to introduce photochromic behavior.^[^ ^10^ ^]^ Because of their wide range of unique properties, inorganic nanoparticles (NPs) are widely used to create hybrid materials together with polymers. Such hybrid materials are used, for example, in structural applications and in biomedical applications.^[^ ^11^ ^]^

Even in bulk material, inorganic NPs can be used to add unique properties to polymers, creating materials such as magnetic elastomers, photoluminescent polymers, or photoresponsive polymer films.^[^ ^12^, ^13^, ^14^ ^]^ Although these examples have been reported, the translation of the unique properties of NPs into hybrid materials remains a challenge, as many of the optical or electrical properties only exist when NPs are well dispersed in the medium.^[^ ^15^ ^]^ This makes the integration of NPs into the matrix a crucial part of the synthesis of these hybrid materials.

In Au‐NPs, for example, the optical properties are strongly dependent on the size, shape, and composition of the surface or chemical environment, which has been widely investigated and exploited in various applications such as sensing, staining, or photothermal applications.^[^ ^16^ ^]^ Previous investigations have shown that Au‐NPs can be incorporated into polymer networks to fabricate bulk materials such as hydrogels or films and retain their plasmonic properties by functionalizing the particles with polymer ligands and blending the functionalized NPs with additional polymer chains to form networks after crosslinking.^[^ ^13^, ^17^, ^18^, ^19^ ^]^ Within these materials, low amounts (mostly below 1 wt%) of the particles can keep their plasmonic properties and be utilized, for example, for photothermal heating. This could also be shown in vitrimer materials, which were created by blending Au‐NPs into the monomer mixture before crosslinking and/or polymerization, and led to hybrid materials with photo‐responsive self‐healing properties.^[^ ^14^, ^20^, ^21^ ^]^ However, the full recyclability of these Au‐NP blended vitrimers is still a challenge because the reformation of the network can lead to a demixing of the NPs and, therefore, a degradation of the plasmonic properties. To solve this issue, the Au‐NP surface can be functionalized with thiols, dithioesters, or trithiocarbonates (TTC) to link polymer chains directly.^[^ ^22^, ^23^, ^24^ ^]^ Using this method, Au‐NPs could be directly functionalized with BCPs obtained by RAFT polymerization and crosslinked to integrate the NPs as fixed network points of the vitrimer, which would prevent particle aggregation and strongly influence the mechanical properties of the material.

Herein, we present a synthesis of Au‐doped vitrimers based on BCPs that are crosslinked as vinylogous urethanes. The Au‐NPs are synthesized using an inverse Turkevich method to obtain spherical Au‐NPs with a narrow size distribution ≈12 nm.^[^ ^25^ ^]^ Different strategies were employed to find a reliable synthesis route to modify the BCPs, functionalize the Au‐NPs, and form the vitrimer networks while preserving the optical properties of the Au‐NPs. Four different di‐ and triblock copolymers were used to synthesize Au‐doped vitrimers with different gold contents. The influence of gold doping on the dynamic mechanical properties and the recyclability of the material, as well as the optical properties after several recycling steps, were studied. Finally, plasmonic heating of the material was investigated to trigger the dynamic bond exchange reaction with visible light at specific wavelengths.

## 2 Results and Discussion

### 2.1 Fabrication of the Au@vBCP Nanocomposites

**Figure 1:**
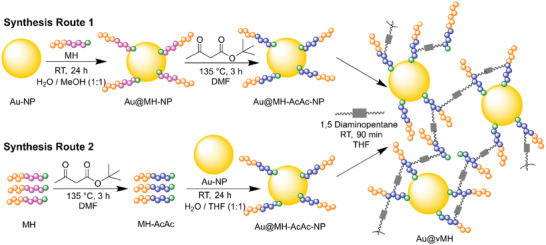
Schematic of the two synthesis routes that were investigated in this study to obtain Au@vBCPs using Au@vMH as an example. Au‐NPs are represented by yellow spheres, MMA (M) units by orange spheres, HEMA (H) units by purple spheres, and acetoacetylated (AcAc) HEMA units by blue spheres, respectively. The TTC group of the polymer chains is represented by a green sphere and the crosslinker by a gray box.

There are multiple possible strategies to synthesize Au‐doped BCPs‐based vitrimers (Au@vBCPs), which can determine the properties of the final product. In this work, two different grafting‐to approaches were chosen and are schematically depicted in **Figure** **1** using the BCP of poly(methyl methacrylate) and poly(2‐hydroxyethyl methacrylate) (PMMA‐*b*‐PHEMA) as an example. Within this work, the nanocomposites will be labeled using abbreviations for the BCP structures shown in Section S2 (Figure S1 and Table S1, Supporting Information) and the theoretical gold:BCP ratio. For example, the Au@vBCP with a 0.5 wt% gold:BCP ratio and being synthesized using PMMA‐*b*‐PHEMA will be labeled Au0.5@vMH.

In both approaches, Au@Citrate‐NPs and the BCPs are first synthesized separately and combined using a ligand exchange procedure in the following step to transfer the NPs to the organic phase before they are crosslinked to form the vitrimer network. The main difference between the two synthesis routes is whether the NPs are introduced before the BCP is acetoacetylated or after, which heavily influences the properties of the materials.

To assure the comparability of the different Au@vBCP materials, the Au‐NPs were always synthesized using an inverse Turkevich method by Schulz et al.^[^ ^25^ ^]^ This method produces narrowly distributed spherical Au@Citrate‐NPs with a mean diameter of 11.8 ± 0.9 nm and very reproducible optical properties (Section S3 and Figures S4 and s5, Supporting Information). The batch size of the reaction is 30 mg of Au‐NPs, which allows for using the same NP batch to investigate a specific parameter like the polymer structure or gold content.

Using RAFT polymerization, according to our previous research, the different BCPs PMMA‐*b*‐PHEMA, PMMA‐*b*‐PHEMA‐*b*‐PDEAEMA (poly(2‐(diethylamino)ethyl methacrylate), D), PDEAEMA), PMMA‐*b*‐PHEMA‐*b*‐PNIPAM (poly(*N*‐isopropyl acrylamide), N), and PMMA‐*b*‐PHEMA‐*b*‐PMMA were synthesized (Figure S1, Supporting Information). These di‐ and triblock copolymers show number average molecular weights determined by ^1^H NMR ${\bar{M}}_{n,\mathrm{NMR}}$ of 16.1–16.9 kDa, number average molecular weights ${\bar{M}}_{n,\mathrm{SEC}}$ of 10.8–11.7 kDa, and dispersities *Đ* of 1.45–1.83 indicating comparable molecular weights of all BCPs due to the use of photoRAFT polymerization. Moreover, PHEMA block weight percentages of 58% are well‐controlled for all backbones (Section S2: Table S1, Supporting Information). Due to the RAFT agent 4‐cyano‐4‐[(dodecylsulfanylthiocarbonyl)sulfanyl]pentanoic acid (CDTPA), all BCP chains are terminated with a carboxy group and a TTC group. This TTC group acts also as an anchoring group to the gold surface.^[^ ^22^, ^26^ ^]^ When using hydrophilic polymers such as PNIPAM or poly(*N*‐acryloylglycinamide), mixing of the polymer and the aqueous Au‐NP suspension is enough to perform a ligand exchange and obtain functionalized Au‐NPs.^[^ ^23^, ^24^, ^27^ ^]^ Due to the poor solubility in water of the BCPs that are used in this work, a simple addition of the polymer to the aqueous Au‐NP suspension will not lead to a ligand exchange, and therefore an adaptation of the procedure is required. In this case, the ligand exchange is done by using a common solvent or, rather, a solvent mixture depending on the solubility of the BCP and BCP‐AcAc (acetoacetylated block copolymer).

In the following section, the preparation of Au@vBCP with synthesis route 2 (Figure 1) is explained in more detail since all materials discussed in this work were synthesized with that method unless specified otherwise. A discussion of synthesis route 1 can be found in Section S4 (Supporting Information).

All of the BCP‐AcAc in this work were soluble in tetrahydrofuran (THF) and in a THF/water mixture (1:1, v/v), which was chosen as the solvent for the ligand exchange. The ligand exchange can be performed by the addition of an equal amount of the polymer solution in THF to the aqueous Au‐NP solution in the desired Au:BCP‐AcAc ratio under vigorous stirring, followed by 1 h of ultrasonication and 22 h of vigorous stirring. To conserve the plasmonic properties of the Au‐NPs in the final material, we need to ensure that the particles do not aggregate during the whole procedure. An essential factor in preventing this from happening was controlling the ionic strength of the Au‐NP suspension, which was done via dialysis against water or aqueous trisodium citrate solutions with different ion concentrations.^[^ ^28^ ^]^

We could show that the initial citrate concentration of the Au‐NP suspension has a strong impact on the properties of the final material, which has been investigated and discussed in Section S5 (Figure S7, Supporting Information). To ensure that the procedure is successful, the as‐synthesized Au@Citrate‐NPs were concentrated threefold via distillation and dialyzed for two weeks against pure water in the optimized protocol. Additionally, we observed that the particles should be used shortly after the dialysis procedure because a long storage period leads to aggregation and cluster formation of the NPs, which is also visible in the final materials (Section S5: Figure S9b, Supporting Information).

**Figure 2:**
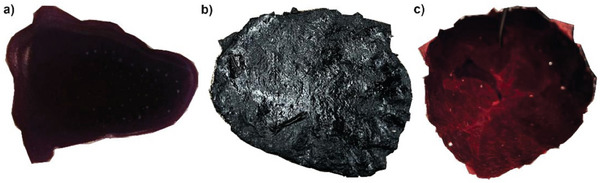
Photographs of (a) drop‐casted colloidal Au0.5@MH‐NPs solution in THF after the addition of the crosslinker before drying, (b) the 0.7 mm thick Au0.5@vMH film after reprocessing, and (c) the same Au0.5@vMH‐film when held into the light.

After the functionalization procedure is completed, the polymer‐coated Au‐NPs (Au@BCP‐NPs) can be recovered via centrifugation or removal of the solvent and redispersed in THF to form a stable colloidal solution. The Au@BCP‐NPs and the excess polymer could then be crosslinked with a diamine linker using the same procedure used for the pure BCP to obtain the vitrimers (**Figure** **2**). The Au@vBCP films show, depending on the thickness, a black‐to‐purple color due to the high absorption coefficient of the Au‐NPs that are distributed throughout the whole film (Figure 2b). When held in the light, the surface plasmon resonance of the Au‐NPs can be seen by the red or purple color, as shown in Figure 2c.

This procedure was used with different gold:BCP ratios (0.1, 0.25, 0.5, 1, and 1.5 wt%) to synthesize the nanocomposites Au0.1@vMH, Au0.25@vMH, Au0.5@vMH, Au1.0@vMH, and Au1.5@vMH. Besides that, the four different BCPs at a constant content of 0.5 wt% Au‐NPs were used to obtain the nanocomposites Au0.5@MH, Au0.5@MHD, Au0.5@MHN and Au0.5@MHM (Section S5, Supporting Information). Depending on the Au content of the nanocomposites, we can synthesize between 2 g and 30 g of the composite using the same Au‐NP and BCP batch. Both the synthesis of the NPs and the BCP could be scaled up using microfluidic reactor systems to increase the synthesis output.^[^ ^29^ ^]^ When doing that, it needs to be considered that the ionic strength of the NPs should be reduced before the ligand exchange and that they should be used shortly after the post‐synthesis treatment to avoid aggregation of the NPs and increase the reproducibility of the synthesis.

To confirm the success of the functionalization and the integration of Au‐NPs, the chemical composition of the Au@MH‐NPs’ and Au1.5@vMH‐film's surfaces were investigated with X‐ray photoelectron spectroscopy (XPS), as shown in Section S6 (Figures S11 and S12, Supporting Information). The presence of both the polymer and gold in the high‐resolution spectra in both samples and the presence of the Au–S linkage in the spectrum of the Au@MH‐NPs suggests the success of the functionalization using the TTC‐groups of the polymer and the integration of the Au‐NPs in the vitrimers. The presence of gold inside the vitrimers could also be verified using graphite furnace atomic absorption spectroscopy (GF‐AAS), showing that 63% of the initially used gold was detected in the final material and listed in Section S5 (Table S2, Supporting Information). When the amount of gold inside the vitrimer is mentioned in the article, it is always referred to as the ratio of Au:BCP, assuming all Au‐NPs were integrated.

Attenuated total reflection Fourier transform infrared (ATR‐FTIR) spectra confirm the successful synthesis of BCPs and their vitrimers (Section S2: Figure S2, Supporting Information). The modification of hydroxyl groups to acetoacetylated groups in the PHEMA block was observed by the decrease of O─H stretching (3272–3427 cm^−1^) and bending (1361–1365 cm^−1^), as well as C─O stretching vibrations in the primary alcohol (1072–1079 cm^−1^). Figure S3 (Supporting Information) shows vitrimer characteristic peaks of vinylogous urethane vitrimers located at 1646–1652 and 1596–1599 cm^−1^, which are assigned to the stretching vibrations of C ═ O and C ═ C bonds in all vitrimer materials.

The gold content in a polymer network is inversely related to the swelling ratio (Section S7: Table S3, Supporting Information). Au‐NPs could act as crosslinking agents within this type of polymer network. When the gold content increases, the degree of crosslinking also increases, resulting in a less flexible network and limiting the ability of the chain to absorb water. The gel fraction, which describes the crosslinked part that is insoluble in solvents, agrees well as the material with the highest gold content exhibits the highest gel fraction, indicating the stronger physical interaction and covalent bond in the network structure. Furthermore, as the concentration of Au‐NPs increases, the available free volume and capacity for water uptake within the matrix may then decrease, thus reducing the swelling ratio. The swelling ratios of di‐ and triblock copolymer vitrimers with the same chemical composition tend not to change notably. Meanwhile, different polymer matrices lead to different swelling ratios depending on the hydrophilicity. At room temperature, PNIPAM is slightly more hydrophilic than PDEAEMA due to the stronger hydrogen bond with water and less bulky side group, so the swelling ratio of Au0.5@vMHN is larger than of Au0.5@vMHD. PMMA is the most hydrophobic of all. In the meantime, the gel fraction does not seem to have a significant influence on the chemical composition of the polymer matrices.

### 2.2 Optical Properties

**Figure 3:**
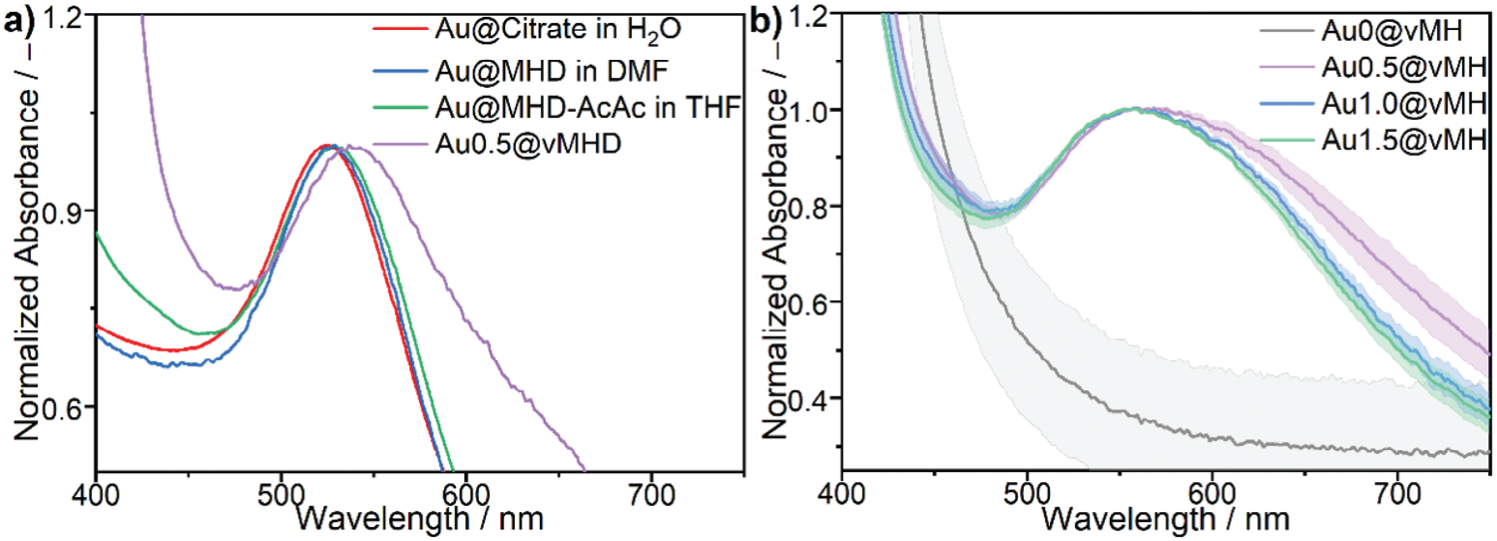
Normalized UV–Vis extinction spectra of: (a) colloidal solutions of Au@Citrate‐NPs in water, Au@MHD‐NPs in *N*,*N*‐dimethylformamide (DMF), Au@MHD‐AcAc‐NPs in THF, and a Au@vMHD vitrimer film using Au@Citrate‐NPs from the same synthesis batch. (b) Au@vMH vitrimer films with gold contents of 0.5 wt%, 1 wt%, and 1.5 wt% (theoretical value) using Au@Citrate‐NPs from the same synthesis batch and the blank vitrimer film. To obtain the spectra of the films, at least three different spots of the film were measured, and the averaged spectra were plotted along their standard deviation.

To utilize the optical properties in the final material, our goal was to prevent aggregation throughout the ligand exchange procedure, the solvent exchange, and the network formation and to achieve a uniform distribution of the NPs in the material. The optical properties of the different functionalized Au‐NP species and the final material throughout the procedure were tracked using ultraviolet–visible (UV–Vis) spectroscopy (**Figure** **3**).

We could observe that the absorption band of the Au@Citrate‐NPs (525 nm in H_2_O) only slightly shifts to higher wavelengths when the particles are functionalized with BCPs (529 nm in DMF) or BCPs‐AcAc (529 nm in THF) and transferred to another solvent due to a change in the chemical environment. In the Au@vBCP films, the optical properties of the Au‐NPs were preserved but shifted to higher wavelengths (539 nm) and slightly broadened. Since the blank vitrimer film does not absorb light in that region, we can ensure that the absorption in that area is due to the surface plasmon resonance of the Au‐NPs. The shift in the absorption peak and slight broadening that we observe might be connected to the change in the chemical environment after the removal of the solvent or a slight clustering of the NPs. Nevertheless, we can assume that no significant aggregation of the Au‐NPs occurred since no shift of the absorption maximum to ≈600 nm nor a change in the color to blue was observed.

Additionally, we observed that factors like the mixing of the single components, the storage time of the colloid or the ionic strength of the Au@Citrate‐NP suspension strongly influence the optical properties of the final product, which we showed by adjusting the trisodium citrate concentration of the initial aqueous suspension (Section S5: Figure S8a, Supporting Information). According to this finding, we adjusted the synthesis procedure to remove the ions in the colloid beforehand and managed to reproduce the Au@vBCP synthesis more than 15 times using five different Au‐NP batches (Section S5: Figure S9a, Supporting Information) while preserving the optical properties of the Au‐NPs using different BCPs (Section S5: Figure S8b, Supporting Information) and gold concentrations (Figure 3b).

Due to the sensitivity of the surface plasmon resonance, we observed absorption maxima of the final products between 534 nm and 560 nm depending on the Au@Citrate‐NP batch, suggesting a slight clustering of Au‐NPs before the initial network formation. A more detailed discussion about factors that cause the clustering and the reproducibility of the optical properties can be found in Section S5 (Supporting Information). Therefore, we exclusively compare the optical properties of Au@vBCP‐films obtained using the same Au@Citrate‐NP synthesis batch. When comparing the extinction spectra depending on the gold content in Figure 3b, we observed that tripling the content of Au‐NPs from 0.5 to 1.5 wt% does not lead to a significant change in the optical properties or significant aggregation. On the contrary, we observed that the vitrimer with the highest gold content in the series showed the least broadening of the spectra, proving that at least within the doping range that we investigated (0.1–1.5 wt% gold), the plasmonic properties of the NPs can be preserved in the vitrimer material. Additionally, we observed that ≈0.25 wt% of Au‐NPs are necessary to ensure that the whole material shows a uniform purple‐to‐black color compared to the yellow color of the blank vitrimer film with a 0.2 mm thickness (Section S5: Figure S10, Supporting Information).

### 2.3 Thermal Properties

Thermal characterizations are essential to understand how the materials respond to changes in temperature. These properties include thermal stability, glass transition temperature *T*_g_, and dynamic viscoelasticity, which depend on the nature of the materials, e.g., chemical composition and structure. Thermal stability typically correlates with thermal degradation temperatures *T*_5%_ and *T*_50%_, which refer to the specific temperatures at which a material loses 5% and 50% of its initial mass, respectively.

Section S8 (Figure S13, Supporting Information) exhibits the thermal gravimetric analysis (TGA) thermograms of Au@vMHs and all obtained parameters are listed in **Table** **1**. Composites are generally one of the key ways to adjust thermal stability.^[^ ^30^ ^]^ In this case, the introduction of Au‐NPs into a network leads to higher thermal stability compared to the corresponding pure matrix.^[^ ^31^ ^]^ This is due to the fact that Au‐NPs, which withstand high temperatures before losing their structure, also act as a crosslinking agent in the polymer matrix and lead to an increase of *T*_5%_ and *T*_50%_ with the increase of the gold content. Specifically, *T*_5%_ rises from 192 to 208 °C, while *T*_50%_ rises from 422 to 429 °C with a larger concentration of Au‐NPs in the vitrimer materials. The values of weight loss for all materials are similar above ≈470 °C. At 600 °C, it has been found that the remaining weight loss of Au1.5@vMH is determined to be the highest value due to the highest non‐degraded inorganic residue (Au‐NPs) in its matrix. We could observe that the residual content (%char) increases with the gold content (from 5% in Au0@vMH to 9% in Au1.5@vMH) to a higher extend than the added mass of the Au‐NPs, which has been observed before in composites of noble metal NPs in polymer matrices. This phenomenon could be explained by the polymer chains attached to the NP surface, which show an increased thermal stability compared to the unbound chains in the matrix.^[^ ^32^ ^]^

**Table 1:** Thermal properties measured by TGA, DSC, and DMA.

Samples	*T*_5%_ ^a)^ [°C]	*T*_50%_ ^a)^ [°C]	%char^a)^ [%]	*T*_g,DSC_ ^b)^ [°C]	*T*_g,DMA_ ^c)^ [°C]
Au0@vMH	192	422	5	67	103
Au0.1@vMH	202	424	5	66	101
Au0.25@vMH	204	424	6	65	100
Au0.5@vMH	205	427	7	65	99
Au1.0@vMH	207	429	9	63	97
Au1.5@vMH	208	429	9	62	97

^a)^ Thermal degradation point (*T*_5%_, *T*_50%_) and %char characterized by TGA

^b)^ mid‐point glass transition temperature (*T*_g_) measured by DSC

^c)^ temperature‐dependent DMA was performed from 200 to 0 °C with deformation amplitude *γ *= 0.01% at angular frequency *ω* = 10 rad s^−1^ to measure *T*_g,DMA_ at the maximum of the tan *δ* curve.

The *T*_g_ of a composite material depends on the nature of both the matrix and the filler. In a composite polymer network, the filler could limit chain flexibility by acting as a physical and/or covalent crosslinker; on the other hand, adding filler could potentially introduce more free volume and/or disrupt the polymer packing process.^[^ ^33^ ^]^ In this work, differential scanning calorimetry (DSC) thermograms (Section S9: Figure S14, Supporting Information) were recorded from glassy to rubbery state (heating), while dynamic mechanical analysis (DMA) curves (Section S10: Figures S15–S24, Supporting Information) were recorded from rubbery to glassy state (cooling). The *T*_g_s obtained from DSC and DMA exhibit similar trends, showing a slight decrease in *T*_g_ with increasing gold content in the materials. For instance, the *T*_g,DSC_s of Au@vMHs are found to be between 62 and 67 °C, whereas the *T*_g,DMA_s exhibit a range of 97 to 103 °C (Table 1). According to these results, the Au‐NP content does not appear to affect the *T*_g_ of the material significantly. Typically, two *T*_g_s are observed in diblock copolymer due to microphase separation. However, we could observe only one *T*_g_ for all materials by both methods in this work. It has been found the *T*_g,DSC_ values are close to the *T*_g_ of homopolymer PHEMA as reported in the literature,^[^ ^34^, ^35^ ^]^ exhibiting a slight increase due to the modification and crosslinking. However, the *T*_g_ of the PMMA block in DSC could not be observed, possibly due to a low change in heat capacity (Δ*C*p), which might result from factors like the small size of the sample and relative amount of the PMMA block, making it difficult to detect. According to the previous study, the *T*_g,DSC_ of PMMA^7.5^ is 106 °C,^[^ ^35^ ^]^ so the *T*_g,DSC_ of PMMA^6.3^ in this study is expected to be slightly lower. On the other hand, the *T*_g,DMA_s of the materials appear to be broad and significantly exceed the *T*_g_ of a homopolymer PHEMA. The values are shifted toward the *T*_g,DMA_ of the homopolymer PMMA^6.3^, which was measured to be ≈102 °C (Figure S21, Supporting Information). This suggests a possible influence of the two blocks on the observed transition. As a result, the data from both DSC and DMA hints at the possibility of microphase separation; however, it could not be directly observed, probably due to limitations in sensitivity.

In triblock copolymer vitrimers with similar Au‐NP content and crosslink density (where modified PHEMA block serves as a crosslinked block), the *T*_g_ is influenced by both the nature of the polymer blocks, for example, the side group and the chain length of each block. Similar to the diblock copolymer vitrimer, only a single broad *T*_g_, which is in between *T*_g_s of all components, is observed in all triblock copolymer vitrimers. This might be due to low weight fraction of third block leading to small microphase separation, which hard to be directly observed.^[^ ^36^ ^]^ When the chain length of the polymer decreases, *T*_g_ also decreases.^[^ ^37^ ^]^ The MHM‐triblock copolymer has shorter chain length of PMMA blocks at both sides compared to the MH‐diblock copolymer. However, the *T*_g,DMA_ is observed to be 97 °C for Au0.5@vMHM (Figure S22, Supporting Information), which is not significantly lower than the *T*_g,DMA_ of Au0.5@vMH (99 °C). It might be assumed that the BCP architecture does not affect much to thermal properties in this case. These materials, Au@vMH and Au@vMHM, contain a TTC group at the chain end. Meanwhile, the other two materials in this work, namely Au@vMHD and Au@vMHN, contain not only the TTC group at the chain end but also tertiary amines and secondary amides in the side groups of the respective blocks. These functional groups potentially bind with Au‐NPs.^[^ ^38^ ^]^ Yin et al. investigated the *T*_g_s of PNIPAM and PDEAEMA, which have similar molecular weights. It was found that PNIPAM has a higher *T*_g_ compared to PDEAEMA.^[^ ^39^ ^]^ This was assigned to the stronger hydrogen bonding between the PNIPAM units and the more flexible side chain of PDEAEMA. Therefore, Figure S23 (Supporting Information) displays the DMA curve of Au0.5@vMHD showing a *T*_g_ of 83 °C, which is relatively lower than 117 °C for Au0.5@vMHN (Figure S24, Supporting Information). PNIPAM has generally a higher *T*_g_ compared to PMMA with the same chain length due to the restricted chain mobility and strong intermolecular interactions of PNIPAM,^[^ ^40^ ^]^ which also leads to a higher *T*_g,DMA_ of the Au@vMHN compared to the Au@vMHM. In addition, secondary amide groups in PNIPAM could also bind to the Au‐NP surface, causing the higher crosslink density and further elevating the *T*_g_.

### 2.4 Mechanical Properties

In this study, we also report the influence of Au‐NPs on the mechanical properties of the composite materials, as inorganic nanofillers are generally known for their reinforcing properties in polymer matrices. By adding reinforcing materials, properties such as modulus, hardness, and strength can be altered. To determine the mechanical properties of the materials, stress‐strain measurements were performed using a universal testing machine (UTM) at room temperature (≈25 °C) and a nanoindenter. Young's moduli *E* can be obtained from both methods. In addition, the nanoindenter also provides the hardness *H* values of the vitrimers. The storage modulus *G'* at 25 °C, obtained from DMA measurements, is also discussed in this section. The Au@vBCPs exhibit a wide range of tensile strength, depending on the gold content and BCP structure.

**Figure 4:**
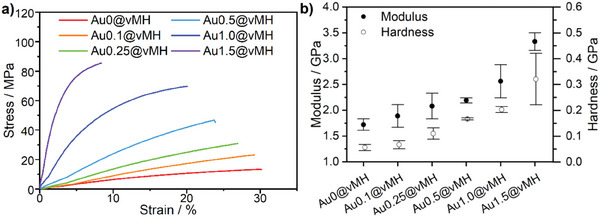
(a) Stress‐strain curves and (b) Young's modulus *E* and hardness *H*, measured by nanoindenter (number of measured spots n = 30) of Au@vBCPs with different gold content, i.e., 0, 0.1, 0.25, 0.5, 1.0, 1.5 wt%.

Au‐NPs have been shown to promote mechanical properties in composite materials by acting as physical crosslinks and reinforcing nanofillers, distributing the stress load evenly due to their high surface area to volume ratio.^[^ ^18^, ^41^ ^]^ The increasing incorporation of gold can significantly increase the stress at break *σ*_m∅_, *E*, and *H*, while reducing the strain at break *ɛ*_m∅_, as shown in **Figure** **4** and **Table** **2**. According to Figures S15–S20 (Supporting Information), the *G’*s show the same trend as well. To be specific, the vitrimer without NPs, Au0@vMH, is an elastic material with *σ*_m∅_ of 13.1 ± 4.9 MPa, *ɛ*_m∅_ of 30.2 ± 2.0%, *E*_t∅_ of 219.7 ± 10.9 MPa, *H*_n_ of 0.056 ± 0.012 GPa, *E*_n_ of 1.72 ± 0.11 GPa, and *G'* of 0.307 GPa. Meanwhile, the material highest gold content, Au1.5@vMH, is found to be the hardest material among all with *σ*_m∅_ of 85.3 ± 5.9 MPa, *ɛ*_m∅_ of 8.3 ± 1.5%, *E*_t∅_ of 779.5 ± 6.7 MPa, *H*_n_ of 0.321 ± 0.099 GPa, *E*_n_ of 3.33 ± 0.17 GPa, and *G'* of 0.542 GPa. This could confirm that gold nanofillers improve the strength and hardness of materials, as they also add more crosslinks to the system by polymer chains and Au‐NPs via a TTC group at the chain end.

**Table 2:** Mechanical and thermo‐mechanical properties measured by UTM, nanoindenter, and DMA.

Samples	*E*_t∅_ ^a)^ [MPa]	*σ*_m∅_ ^a)^ [MPa]	*ɛ*_m∅_ ^a)^ [%]	*H*_n_ ^b)^ [GPa]	*E*_n_ ^b)^ [GPa]	*G'* ^c)^ [GPa]	*G'*_rubber_ ^c)^ [MPa]	*E*_a_ ^d)^ [kJ mol^−1^]
Au0@vMH	219.7 ± 10.9	13.1 ± 4.9	30.2 ± 2.0	0.056 ± 0.012	1.72 ± 0.11	0.307	0.609	32.9 ± 3.0
Au0.1@vMH	319.0 ± 9.0	23.1 ± 3.1	29.2 ± 1.8	0.067 ± 0.016	1.89 ± 0.22	0.353	0.664	36.8 ± 4.1
Au0.25@vMH	406.3 ± 6.9	30.8 ± 1.1	26.9 ± 1.4	0.110 ± 0.022	2.08 ± 0.25	0.383	0.669	37.7 ± 3.2
Au0.5@vMH	466.0 ± 8.5	44.2 ± 2.9	23.9 ± 2.0	0.167 ± 0.004	2.19 ± 0.05	0.479	0.672	38.4 ± 2.7
Au1.0@vMH	604.5 ± 12.4	69.8 ± 6.6	20.1 ± 3.9	0.203 ± 0.012	2.56 ± 0.32	0.527	0.675	55.3 ± 2.0
Au1.5@vMH	779.5 ± 6.7	85.3 ± 5.9	8.3 ± 1.5	0.321 ± 0.099	3.33 ± 0.17	0.542	0.681	61.7 ± 3.4

^a)^ Stress at break (*σ*_m∅_), strain at break (*ɛ*_m∅_), and *E*‐modulus (*E*_t∅_) determined by stress‐strain measurements at room temperature with a strain rate of 10 mm/min (n = 3)

^b)^ *E*‐modulus *E*_n_ and hardness *H*_n_ determined by nanoindentation testing (n = 30)

^c)^ temperature‐dependent DMA was performed from 200 to 0 °C with deformation *γ* = 0.01% and angular frequency *ω* = 10 rad s^−1^ to measure *T*_g,DMA_ at the maximum of the tan *δ* curve, and storage modulus (*G'*) and rubbery plateau (*G'*_rubber_) at 25 and 200 °C, respectively

^d)^ stress relaxation DMA was performed from 160 to 110 °C with 0.01% deformation *γ* to determine the activation energy from an Arrhenius plot of average stress relaxation time and temperature.

According to the previous study, the BCP architecture also influences the mechanical properties of a BCP‐based vitrimer.^[^ ^6^, ^35^ ^]^ Similarly, for composites, the diblock (MH) and triblock (MHM) copolymers based on Au‐NP vitrimers also show different properties due to the network packing and chain length of each block. Figure S22 (Supporting Information) shows the DMA temperature sweep of Au0.5@vMHM. The triblock copolymer vitrimer has a *G'* of 0.641 GPa, which is significantly higher than for the diblock copolymer vitrimer with the same chemical composition (PMMA and PHEMA) and gold content. This is due to the higher chain length of PMMA in the diblock copolymer, resulting in a more elastic Au0.5@vMH. On the other hand, the Au0.5@vMHM has shorter chain length of PMMA at both sides, affecting to the smaller PMMA microdomain. Moreover, PMMA blocks in Au@vMHM being located in the same or two different microdomains during network formation, create loops or bridges. The bridges allow the mechanical properties to be improved, compared to Au@vMH, in which the PMMA block is only located in a single microdomain. These loops could also lead to the shorter domain spacing, which is related to the higher *G'*. This phenomenon has also been observed in our previous study.^[^ ^6^, ^35^ ^]^ The change of the third block from PMMA to PDEAEMA or PNIPAM influences the mechanical properties. A long side group in a polymer can also increase the flexibility of the structure, making the material softer and more elastic. So, the introduction of PDEAEMA or PNIPAM as the third block leads to lower *G'* compared to using PMMA as the third block as well as in the diblock copolymer vitrimers. Additionally, Au0.5@vMHD has a slightly lower *G'* (0.294 GPa) than Au0.5@vMHN (0.311 GPa). This could be due to the stronger hydrogen bonding and shorter side group of PNIPAM (Figures S23 and S24, Supporting Information).

### 2.5 Thermo‐Mechanical Properties

According to the fact that vitrimers exhibit unique thermo‐mechanical properties due to their dynamic covalent crosslinks, *G*′ and *G*″ of composite materials have been studied as well as activation energy *E*_a_ and average stress relaxation time at 150 °C, < *τ* >_150_. Firstly, the amplitude sweeps were conducted at 110 and 200 °C to select the shear amplitude for temperature‐dependent DMA (Section S11: Figures S25–S30a, Supporting Information). The shear amplitude was selected from the linear viscoelastic regime (LVER), which ranges from 0.001 to 1% for Au‐NP vitrimers in this study. In addition, a frequency sweep was performed from 1 to 100 rad s^−1^, as shown in Figures S25–S30b (Supporting Information). So, the temperature‐dependent DMA at an angular frequency of 10 rad s^−1^ and a shear amplitude of 0.01% was performed. As discussed in the previous section, *G'* of Au@vMHs in the glassy state at 25 °C shows the same trend as the thermal degradation temperature, Young's modulus *E*, hardness *H*, and *σ*_m∅_, *G'*_rubber_ (or *G'* at 200 °C) and increases with increasing Au‐NP content in the composite vitrimer. This is due to the good thermal stability of Au‐NPs, as they can still act as physical crosslinks and reinforce the materials, which restricts the network mobility and enhances the stiffness of the vitrimers (Table 2; Figures S15–S20, Supporting Information).

The Au@vBCP vitrimers in this work exhibit the rubbery plateau regime at temperatures above 105 °C. The stress relaxation moduli *G*s were recorded above *T*_g_ by applying a torsional deformation of 0.01% at 110, 120, 130, 140, 150, and 160 °C. Non‐normalized and normalized stress relaxation curves are shown in Figures S25–S30c,d (Supporting Information). In order to prove the associative exchange reaction assumption, it is important that the initial relaxation moduli *G*_0_s are approximately similar for all the measured temperatures (Figures S25–S30e, Supporting Information). Due to the chemically incompatible blocks of polymer chains, a stretched exponential decay is a more appropriate fit as multiple relaxations are expected. This gives the parameters of the relaxation distribution *β* and < *τ* > from each temperature as listed in Tables S4 and S5 (Supporting Information).^[^ ^42^, ^43^ ^]^ The linear relationships between ln < *τ* > and 1000/*T* were therefore plotted as shown in Figures S25–S30f (Supporting Information) to calculate the *E*_a_ reported in Table 2. Note that *E*_a_ therefore is an apparent activation energy related to overlaying different relaxations.

As mentioned earlier, the observed trends of thermal and mechanical properties show that with the increasing gold surface due to higher NP content in the vitrimer network, more interaction between the gold surface and the polymer matrix can occur, resulting in the restriction of chain mobility and rigidity of the network. The trend of *E*_a_ agrees well, as the higher the number of crosslinks per Au‐NP, the more energy is required in the topological rearrangement and dynamic exchange reaction in these vinylogous urethane vitrimer transamination. Moreover, it also influences the slower relaxation process, leading to longer relaxation time when the gold concentration in the vitrimer network is increased. Au@vMHs show *E*_a_ ranging from 32.9 ± 3.0 to 61.7 ± 3.4 kJ mol^−1^ and < *τ* >_150_ ranging from 2.5 to 7.5 s.

As mentioned above, Au‐NPs are stable, contributing to the vitrimer network's good (thermo‐)mechanical properties and overall durability. The measurements are typically conducted under controlled atmospheric conditions, i.e., N_2_ atmosphere for DMA measurements, and temperature of 25 °C and a relative humidity of 28–32% for UTM measurements, to ensure consistency and reliability in evaluation of materials. Ambient factors, however, may also have an impact on the performance. For instance, while being based on hydrophobic PMMA, the material networks could absorb water at high levels of humidity, as detailed in swelling properties (Section S7: Table S3, Supporting Information). Additionally, plasmonic heating of AuNPs may soften the vitrimer matrix, when light intensity reaches a high enough energy. In the case of repeated mechanical stress, there is no agglomeration observed after several compression cycles and the mechanical properties of pristine and recycled materials are comparable, as will be discussed in more detail later. These factors potentially alter the material properties. For future studies, it is interesting to note these factors as key areas for future research to better understanding their impact on the short‐ and long‐term performance and durability.

### 2.6 Reprocessing, Reshaping, Self‐Healing, and Shape‐Memory

**Figure 5:**
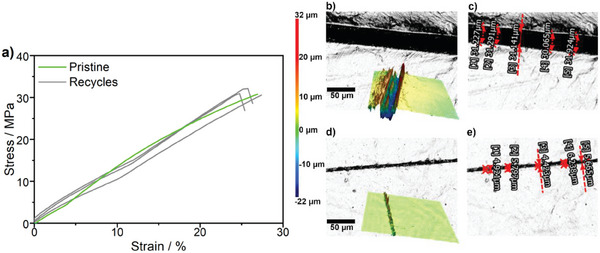
(a) Stress‐strain curves of Au0.25@vMH vitrimer; pristine (green) and 3 recycles (gray). (b) Confocal microscope image and surface profile of vitrimer (Au0.25@vMH), and (c) width of the cut surface when scratched (0 min). (d) Image and surface profile, and (e) width of the cut surface after heating with a heat gun at 140 °C for 1 min. The scratch was made with a cutter. The scale bar is 50 µm.

The dynamic associative exchange reaction of vitrimers results in their permanent as well as dynamic crosslinks. Therefore, these materials can be reshaped and reprocessed by rearrangement of the crosslinked structures at high temperatures. These materials can autonomously heal minor damage owing to their adaptable network and exhibit shape‐memory behavior, returning to their original shape when exposed to stimuli such as heat and, in this case, light.^[^ ^44^ ^]^ **Figure** **5**a confirms that the materials obtained in this work, after grinding into small pieces and hydraulic pressing (150 °C, 10 kN, 2 min), as displayed in Section S12 (Figure S31 and Video S1, Supporting Information), can be reprocessed like thermoplastics while maintaining the mechanical performance of thermosets. The mechanical properties between virgin and recycled materials (3 cycles) demonstrate statistically that they do not differ according to the mean comparison (Table S6 and Figure S32, Supporting Information). The vinylogous urethane chemistry enables Au@vBCP‐films to self‐repair after damage, such as scratches. The self‐healing behavior of the materials can be observed with the naked eye, as depicted in Figure S33 and Video S2 (Supporting Information). The material has been cut and then heated by heat gun at 130 °C. The scratch disappeared gradually as a function of time as shown in Figure S33b,c (Supporting Information), at 30 and 60 s, respectively. This behavior can also be quantifiably observed for the sample Au0.25@vMH under a confocal microscope in Figure 5b–e and in Section S12 (Figures S34–S50, Supporting Information) for other materials studied in this work. In this experiment, the scratch was applied on the surface of the material by a cutter and healed by heating at 140 °C for 60 s. 3D surface profiles have been reconstructed for damaged and healed materials to study the depth and width of an applied cut. The damages introduced by a cutter are ≈10–30 µm depth and 10–40 µm width. All samples display more than 90% efficiency of self‐healing ability triggered by heat within 60 s due to the dynamic transamination reaction (Table S7, Supporting Information). It can be observed that the Au@vBCP composites have a similar self‐healing ability compared to the one without Au‐NPs within the observed period. This also confirms that the presence of Au‐NPs in the network does not interfere with the dynamic exchange reaction of vinylogous urethane vitrimers. Therefore, the Au@vBCP composites also exhibit shape‐memory properties by returning to their original form when exposed to the external stimuli heat (Figure S51 and Video S3, Supporting Information) and light (Figure S52 and Video S4, Supporting Information) within 60 and 40 s, respectively.

This observation is consistent with different vitrimer nanocomposites based on Au‐NPs, which showed a good self‐healing ability.^[^ ^14^, ^20^, ^21^ ^]^ However, the full reprocessability of the materials has not been shown in those works. It is significant to point out that the efficiency of dynamic exchange reaction in composite materials is influenced by the NPs’ size, surface area‐to‐volume ratio, and volume fraction of the NPs in the composite. Huang et al. fabricated nanosilica‐vitrimer composites (∼165 nm), where a cut in the blank vitrimer matrix self‐healed slightly faster than in other composites due to hindered topological rearrangement. The small size of the filler particles does not impede the dynamic exchange reaction and ensures uniform dispersion within the matrix. In the case of nanocomposites with a high inner surface area, such as vitrimer networks with aligned carbon nanotube sheets, they exhibited rapid self‐healing and no agglomeration of aligned carbon nanotube sheets, which might have caused reduced materials performance.^[^ ^45^ ^]^

**Figure 6:**
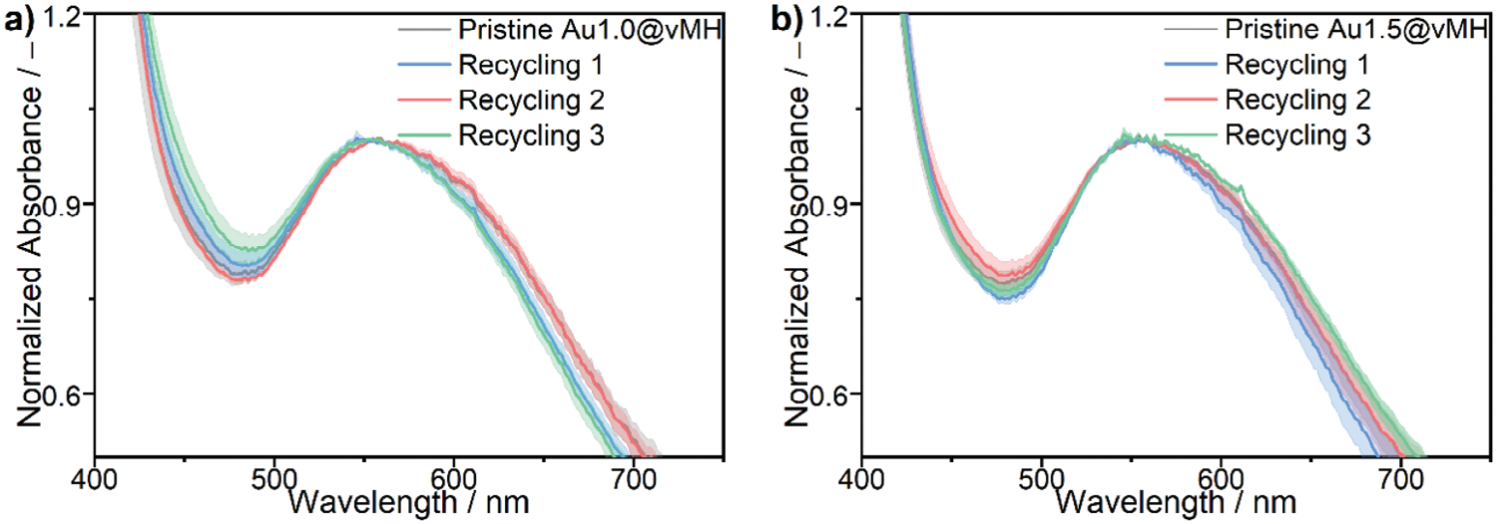
UV–Vis spectra of (a) the Au1.0@vMH film and (b) the Au1.5@vMH film after three recycling steps. The films were measured at four different points, and the average of the spectra, along with their standard deviation, was taken.

Besides the self‐healing and mechanical recyclability, we also investigated the conservation of the optical properties to demonstrate that the Au‐NPs do not demix and form extensive clusters when the material is reformed at 150 °C and 10 kN pressure. This was investigated using UV–Vis spectroscopy under consideration of several spots in the film, which were recycled multiple times (**Figure** **6**). Table S8 and Figure S53 (Supporting Information) provide statistical evidence that the wavelength at the peaks of virgin and recycled materials remains similar.

Doing that, we could observe that even after multiple recycling steps, the optical properties of the film remain, and no additional broadening of the spectrum can be observed. Combined with the conservation of the mechanical properties, we can conclude that the Au‐NPs were successfully integrated as fixed parts of the network, as they are crosslinks with a large functionality.

The associative nature of the vitrimer network causes the network density to remain unchanged during reprocessing. This tight permanent network that surrounds every Au‐NP hinders them from migrating within the composite and segregating into clusters during reprocessing, leading to a conservation of the optical properties and the initial network density. The significance of the integration of the NPs as fixed parts of the network versus the blending of NPs in a vitrimer network has been investigated in a recent study, showing that the material properties were only retained when the NPs are a fixed part of the network.^[^ ^46^ ^]^ Otherwise, a segregation and clustering of the particles was observed, similar to what we observed with films that showed an incomplete ligand exchange (Section S5: Figure S7, Supporting Information). This suggests that if the NPs are just blended inside the material, they possess enough mobility to move within the matrix and form clusters at the high temperatures and under the high pressure used during reprocessing.

### 2.7 Investigation of the Plasmonic Heating Efficiency

The thermo‐reversible properties that we showed present the opportunity to heal cracks or reform the material by increasing the temperature above a certain value. To prevent a temperature induced softening of the whole material during the healing process, we investigated a method to locally heat the material in the damaged area by utilizing plasmonic heating with visible light as an external stimulus. Plasmonic heating using light in the surface resonance frequency of Au‐NPs is widely known as a method for photothermal therapy in biomedical applications with colloidal NP systems but was also demonstrated in vitrimers blended with low amounts of Au‐NPs (<0.5 wt% Au‐NPs).^[^ ^14^, ^19^, ^20^, ^21^ ^]^

**Figure 7:**
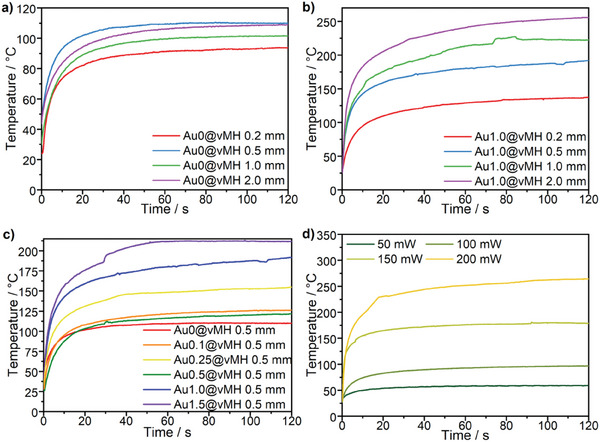
Temperature of the film dependent on the laser's (*λ* = 532 nm, 200 mW, e^−2^ spot size *A* = 3.2 mm^2^) irradiation time of (a) the Au0@vMH‐films with different thicknesses, (b) Au1.0@vMH films with different thickness, (c) Au@vMH films with different gold contents, and (d) the Au0.5@vMH with 2.0 mm thickness using different laser power, i.e., 50, 100, 150, and 200 mW (1.35–5.4 W∙cm^−2^).

However, the dependency of the heating efficiency on the Au‐contents and film thickness has not been extensively investigated. To induce the plasmonic heating, we used a green laser (*λ* = 532 nm) according to the plasmon resonance peak that we observed in the UV–Vis spectra (Section S13: Figure S54, Supporting Information) with different intensities on the Au@vMH‐films with different Au‐contents from 0.1–1.5 wt% and film thicknesses from 0.2–2 mm. The temperature change from the starting temperature of ≈24 °C was recorded using a laser thermometer in dependence of irradiation time in **Figure** **7**. The experimental setup and the results of all measurements are shown in Section S13 (Figures S54–S56 and Table S9, Supporting Information).

Our investigation showed a sharp increase in temperature (up to 210 °C after 10 s) upon irradiation comparable to what was shown previously with Au‐NP blended vitrimers.^[^ ^14^, ^20^, ^21^ ^]^ In all measurements, the heating reaches a plateau after up to 60 s. While the blank vitrimer films also heat up when they are irradiated with the laser, the films that contain Au‐NPs show much higher heating performances leading to a temperature peak of 260 °C (Au1.5@vMH_2 mm) compared to 109 °C in the blank vitrimer (2 mm, 200 mW). The high absorption coefficient of plasmonic Au‐NPs leads to an efficient heating of the material upon irradiation even if only a small amount of filler is added. Due to their high heat conductivity (317 W m^−1^ K^−1^ for gold),^[^ ^47^ ^]^ the Au‐NPs transfer the heat rapidly to the polymer chains, which are directly attached to the particles. This leads to strong local heating near the illuminated NPs, which travels relatively slow through the polymer matrix due to its comparably low heat conductivity (0.192–0.199 W m^−1^ K^−1^ for PMMA and 0.26 W m^−1^ K^−1^ for PHEMA).^[^ ^48^ ^]^ Even after a longer irradiation time, we could observe with an infrared camera that the heating is mostly present locally and rapidly decreases with the distance to the illuminated spot. This behavior is generally known for plasmonic nanostructures, showing that while the temperature still increases at short distances from the illuminated structure, the maximum temperature that the spot reaches depends on the proximity to the illuminated spot.^[^ ^21^, ^49^ ^]^ This means that certain areas can be specifically targeted with a laser to activate the self‐healing without softening the whole material because of the slow temperature increase in the area outside the illuminated spot. We could show that the shape memory of a bent sample could be triggered by carefully positioning it into the beam path (Figure S51 and Video S4, Supporting Information).

We could also observe that higher film thicknesses lead to a strong increase in the heating performance of the Au@vMH films (119 °C at 0.2 mm and 260 °C at 2 mm thickness) due to the higher amount of active Au‐NPs per area while the film thickness of the blank vitrimer only slightly increases the heating performance (93 °C at 0.2 mm and 113 °C at 2 mm thickness). We expect that the increase of the thickness further increases the heating performance as long as the whole cross‐section is illuminated by the laser, which is dependent on the NP concentration and the laser intensity. Due to the continuous increase of heat we assume that a Au1.5@vMH film with the thickness of 2.0 mm can still be penetrated by the laser with an intensity of 5.4 W cm^−2^ but we expect that the maximum penetration depth is close to 2.0 mm due to the transmission of the laser light we observed during the experiments. Similarly, we could observe the increase in heating performance when the gold content inside the film is increased due to the higher concentration of plasmonic particles. Apart from the composition of the film, the power of the laser also influences the heating performance due to higher amounts of energy that is introduced into the system leading to a temperature increase of 97 °C at 100 mW compared to 260 °C at 200 mW laser power. Concluding the investigation, we could show that introducing Au‐NPs strongly increases the heating efficiency, especially for higher film thicknesses, and the local temperatures that are necessary to activate the self‐healing can be easily reached in short illumination times. Additionally, we could show that the shape memory can be activated using plasmonic heating (Figure S51 and Video S4, Supporting Information), and small cuts can be healed.

## 3 Conclusion

A synthetic route of BCP‐based vinylogous urethane vitrimers reinforced with Au‐NPs is successfully developed. The key to its success lies in the interaction between the Au‐NPs and the polymer chains in such hybrid materials, which could be achieved by the anchoring groups along or at the end of the polymer chains. Due to the use of a specific RAFT agent, all BCPs contain TTC groups at the chain end, which could be directly anchored to the gold surface of the NPs to integrate the NPs as fixed points in the network and prevent aggregation. A ligand exchange procedure is developed that shows a crucial impact of the synthesis parameters on the NP distribution and optical properties, which is adapted to the different di‐and triblock copolymers using different gold contents. The resulting nanocomposites after network formation show the conservation of the plasmonic properties of the Au‐NPs and the mechanical properties even after several reprocessing cycles showing a recyclability of the material. The optical properties allow for effective plasmonic heating that can utilize intense light as an external stimulus to trigger the self‐healing and shape memory of the material. The resulting materials also demonstrate the improvement of thermal, mechanical, and thermo‐mechanical properties due to the incorporation of Au‐NPs as additional network points. Notably, the use of Au@BCP‐NPs as a base of the vitrimer nanocomposites is a great example of how properties that are unique to NPs, like surface plasmon resonance, can be integrated into bulk material and express desirable processability, self‐healing, and shape‐memory that can be triggered by both heat and light. The successful integration of Au‐NPs into the BCP‐based material thus enhances its overall performance, e.g. thermo‐mechanical properties, without compromising the characteristic properties such as self‐healing of the base vinylogous urethane vitrimer and broadens the utility of the material due to the light‐induced heating and self‐healing possibilities.

## 4 Experimental Section

### Materials

Trisodium citrate dihydrate (99%, Honeywell, Germany), citric acid (99%, Sigma–Aldrich, Germany), chloroauric acid trihydrate (HAuCl_4_∙3 H_2_O, 99%, Sigma–Aldrich, Germany), tetrasodium 2,2′,2′′,2′′′‐(ethane‐1,2‐diyldinitrilo)tetraacetate (EDTA, 97%, Sigma–Aldrich, Germany), 4‐cyano‐4‐[(dodecylsulfanylthiocarbonyl)sulfanyl]pentanoic acid (CDTPA, 97%, TCI, China), *tert*‐butyl acetoacetate (Alfa Aesar, 98%, Germany), 1,5‐diaminopentane (>98%, TCI, Belgium), *N*,*N*‐ dimethylformamide (DMF, >99.5%, VWR Chemicals, France), tetrahydrofuran (THF, 99.7%, Fisher Scientific, Germany), methanol (99%, Merck KGaA, Germany), 1,4‐dioxane (99%, Grüssing GmbH, Germany), cyclohexane (95%, Fischer Scientific, Belgium), and *n*‐hexane (95%, Fisher Scientific, Austria) were purchased. Chloroform‐*d*_1_ (CDCl_3_, 99.8%, Eurisotop) and THF‐*d*_8_ (TDF, 99.5%, Deutero) were used without further purification. The monomers methyl methacrylate (MMA, 99%, Sigma–Aldrich, Japan), 2‐hydroxyethyl methacrylate (HEMA, 97%, Sigma–Aldrich, Germany), and 2‐(diethylamino)ethyl methacrylate (DEAEMA, 99%, Sigma–Aldrich, USA) were filtrated over a basic aluminum oxide (98%, Sigma–Aldrich, Germany) column to remove the inhibitor before use. *N‐*isopropyl acrylamide (NIPAM, 98%, TCI, Japan) was recrystallized three times from cold n‐hexane before use. Other reagents and solvents were used as received.

Throughout all experiments, ultrapure water (resistivity of 18.2 MΩ∙cm at 25 °C) from a Synergy Millipore water purification device was used.

### Synthesis—Synthesis of Au‐NPs

The Au‐NPs were synthesized via an inverse Turkevich method according to a procedure of Schulz et al. to obtain Au‐NPs in aqueous medium with a narrow size distribution and a mean diameter of 12 nm.^[^ ^25^ ^]^

Six hundred milliliters of an aqueous solution of trisodium citrate (2.75 mmol) and 200 mL of an aqueous solution of citric acid (2.75 mmol) were mixed under stirring in a 2 L beaker. In another 1 L beaker, 64 mg HAuCl_4_∙3 H_2_O (163 µmol) was dissolved in 200 mL water to obtain the Au(III) precursor solution (813 µmol). The buffer solution was covered and heated to its boiling point where it was kept for 15 min under vigorous stirring (800 rpm, will be referred to as stirring in the following section). The Au(III) precursor solution was heated to a temperature of 90–100 °C while stirring. After 15 min of stirring at the boiling point of the buffer solution, 10 mg of EDTA (26 µmol) and the hot Au(III) precursor solution were quickly added to the buffer solution under stirring. The reaction mixture was stirred for 20 min. In the first 2 min after the addition of the Au(III) precursor, the transparent solution changed color first to blue, then to purple, and finally to red.

After 20 min, the beaker was removed from the hot plate, stirred y until the solution cooled to 70 °C, and then the reaction mixture was transferred to a round‐bottom flask. The volume of the red colloidal Au‐NP solution was carefully reduced under vacuum to 200–300 mL. Finally, the colloidal Au‐NP solution was dialyzed in a cellulose dialysis bag (Spectra/Por) with a flat diameter of 25.5 mm and a molecular weight cut‐off of 6–8 kDa against 5 L water for 2 weeks. The dialysis water bath was changed twice a day.

### Synthesis—Synthesis of BCPs

In this work, the BCP of poly(methyl methacrylate) (PMMA) and poly(2‐hydroxyethyl methacrylate) (PHEMA), PMMA‐*b*‐PHEMA), was synthesized via photoRAFT polymerization according to the method in previous studies.^[^ ^6^, ^35^ ^]^ A 0.20 g (0.495 mmol) CDTPA and 5.54 mL (52.0 mmol) MMA were mixed in 21 mL 1,4‐dioxane in a 30 mL glass vial. A 25 wt% concentration of monomer and RAFT agent was maintained. Additionally, 1 wt% DMF was added as an internal standard for ^1^H NMR. The solution was degassed with nitrogen for 20 min, and then heated to 70 °C in a 500 mL beaker as a water bath. For initiation of the polymerization a green light‐emitting diode (LED) with an intensity maximum of 522 nm and light intensity of 1.13 mW cm^−2^ was chosen, since CDPTA absorbs at 400–530 nm. The LED strip was attached to the inner side of an aluminum cylinder with a diameter of 16.3 cm and a height of 15.0 cm.^[^ ^50^ ^]^ To control the degree of polymerization and dispersity, the monomer conversion was not allowed to exceed 60% by tracking the monomer conversions through kinetic studies from ^1^H NMR spectra over time. Monomer conversions were determined by the decrease in vinyl signals compared to before polymerization (Equation S1, Supporting Information). To terminate the polymerization, the glass vial was placed in an ice bath and the solution was exposed to air. The polymer product was then purified by precipitation in cyclohexane to remove unreacted monomer and RAFT agent. After 3 times of precipitations, the light yellowish product was dried *in vacuo* at 30–40 °C for 24 h. The same procedure was used to synthesize the second block (PHEMA). The degree of polymerization of this block was calculated from the PMMA macroRAFT agents. Specifically, PMMA (2.2 g, 22 mmol) and HEMA (4.7 mL, 38 mmol) were dissolved in 28.0 mL 1,4‐dioxane and 0.28 mL DMF. The solid concentration (polymer and monomer) was reduced from 25 to 20 wt% compared to the first block to obtain a lower viscosity solution. THF‐*d*_8_ was used as a solvent for the ^1^H NMR measurements in this step. After the purification and drying process, the yellowish solid PMMA‐*b*‐PHEMA was retained for the next steps.

In addition, PMMA‐*b*‐PHEMA was further polymerized to obtain three different triblock copolymers using the same procedures as the second block. Specifically, diblock copolymer and the third block monomer were dissolved in 20 wt% of 1,4‐dioxane and 1 wt% and further polymerized via photoRAFT at 70 °C under nitrogen atmosphere and green LED light irradiation. These third blocks were PMMA, poly(2‐(diethylamino)ethyl methacrylate) (PDEAEMA), or poly(*N‐*isopropyl acrylamide) (PNIPAM). In summary, there is one diblock copolymer and three triblock copolymers with similar molecular weights (16.5 kDa) and PHEMA block weight percentages (58%).

### Synthesis—Acetoacetylation of BCPs

One gram of PMMA‐*b*‐PHEMA diblock copolymer (66.5 µmol) and *tert*‐butyl acetoacetate (0.67 mL, 4.46 mmol) were dissolved in 5.3 mL DMF in a round bottom flask. The solid concentration was maintained at 20%, and the ratio of hydroxyl groups in the diblock copolymer to *tert*‐butyl acetoacetate was 1/1 (full modification). The modification from hydroxyl to acetoacetate groups via condensation reaction was performed at 135 °C in a distillation system to remove the by‐product, *tert*‐butyl alcohol.^[^ ^51^ ^]^ 92–97% conversion was targeted. After 3 h, the solvent and the by‐product were then removed by an oil pump at 0.1 mbar. The dark yellowish product was cooled down to room temperature for the next step. In addition, acetoacetylated triblock copolymers were also prepared using the same method as for the diblock copolymer. Following this procedure, roughly 1 g of acetoacetylated BCP (BCP‐AcAc) was obtained.

### Synthesis—Functionalization of Au‐NPs with BCP Chains

A 0.27 g of a PMMA‐*b*‐PHEMA‐*b*‐PDEAEMA polymer was dissolved in 20 mL of methanol and mixed with 20 mL of the aqueous Au‐NP suspension (4.5 mg of Au). The homogeneous violet mixture was stirred vigorously for 1 h, ultrasonicated for 1 h, and finally stirred for another 22 h. The foaming violet suspension was centrifugated at 8000 rcf for 60 min. The light violet supernatant was carefully dried *in vacuo* to prevent strong foaming until a purple residue remained. The purple/black precipitant and the purple residue were dissolved in 3 mL of DMF by shaking and ultrasonicating. Finally, 3 mL of a suspension of functionalized Au@BCP‐NPs in DMF was obtained.

### Synthesis—Functionalization of Au‐NPs with BCP‐AcAc

The functionalization of the Au@Citrate‐NPs with BCP‐AcAc chains was performed using the different BCPs, different solvent mixtures (DMF/water and THF/water), and polymer/Au ratios. An example of the most reliable synthesis route is described in the following with a polymer/Au ratio of 1/0.005 (0.5 wt%). The method can be applied to any synthesized BCPs and different gold contents while keeping the solvent mixture ratios.

One gram of BCP‐AcAc (4.46 mmol acetoacetate groups for PMMA‐*b*‐PHEMA) was dissolved in 30 mL of THF. The yellow polymer solution was quickly added to 30 mL of an aqueous Au@Citrate‐NP (5 mg of gold) under vigorous stirring. The slightly turbid violet mixture was vigorously stirred for 1 h, ultrasonicated for 1 h, and finally stirred for another 22 h. The mixture was vigorously stirred at room temperature for another 24 h after the ultrasonication. THF was removed from the mixture using a rotary evaporator (40 °C water bath, 230 mbar) to obtain a turbid, violet suspension. The suspension was centrifugated at 2000 rcf for 1 h, and the purple precipitant dissolved in 15 mL of THF. The turbid supernatant was dried in vacuum to obtain a slightly purple residue. The residue was dissolved in the purple solution of the precipitate using ultrasonication, and a clear purple solution of BCP‐AcAc‐functionalized NPs (Au@BCP‐AcAc) in THF was obtained.

The content of gold NPs was varied by increasing the volume of the aqueous NP solution while keeping the polymer mass and the THF/water ratio constant.

### Synthesis—Network Formation of BCP‐Based Vitrimers

One gram of acetoacetylated diblock copolymer (4.46 mmol acetoacetate group) was dissolved in 4 mL of THF in a glass vial and stirred to homogeneity. The concentration of substances was kept at 40 wt%. In order to drive a transamination as a dynamic exchange reaction, a molar excess of the amino groups is required, so the ratio of acetoacetate group to amino group was 1/1.1, or *R*‐value is 0.91. The crosslinking agent, 1,5‐diaminopentane (0.29 mL, 2.45 mmol), was added and stirred at room temperature (24 °C) for 1 h. It was then air dried on a Teflon sheet with folded edges and placed in a vacuum oven at 100 °C for 24 h, followed by 150 °C for 30 min to fully cure the vitrimer network. The triblock copolymer vitrimers were crosslinked by the same procedures. The resulting materials are transparent and yellowish. Finally, all the vitrimers obtained were placed between two Teflon sheets, hydraulically pressed to the desired thickness, and cut into the desired shape for further studies.

### Synthesis—Vitrimer Formation of Au@BCP‐NPs

To form the vitrimer network, 0.290 mL of 1,5‐diaminopentane (2.45 mmol, 1.1 molar excess with respect to the total acetoacetate functions) was added to 15 mL of the purple Au@BCP‐NP suspension (1 g BCP‐AcAc, 5 mg Au, 4.46 mmol acetoacetate groups). The solution was stirred vigorously for at least 90 min, drop‐cast onto a Teflon sheet, and air‐dried under ambient pressure and temperature. To completely remove the solvent, the purple vitrimer film was dried in a vacuum oven using the same procedures as the abovementioned. After drying, roughly 1 g of the Au‐doped BCP‐based vitrimer (Au@vBCP) was obtained

### Methods—Proton Nuclear Magnetic Resonance (^1^H NMR) Spectroscopy

^1^H NMR spectra were obtained using a BRUKER AVANCE II 400 MHz instrument at room temperature (296 K) from 16 to 0 ppm with CDCl_3_‐*d*_1_ and THF‐*d*_8_ as solvents and DMF as an internal standard. Data was interpreted using MestReNova (Version 9.0.1, Mestrelab Research S.L.).

### Methods—Fourier Transform Infrared (FTIR) Spectroscopy

A Bruker FT‐IR Vertex 70 with a diamond attenuated total reflection (ATR)‐probe provided ATR‐FTIR spectra ranging the wavenumber region from 4000 to 600 cm^−1^ at room temperature with 64 scans and a resolution of 4 cm^−1^. Spectra were processed by OPUS 5.5 software.

### Methods—Size Exclusion Chromatography (SEC)

All values of number average molecular weight and dispersity of the polymer samples were measured using an AGILENT 1260 INFINITY system equipped with PSS SDV separation columns and an AGILENT 1260 Series RID refractive index detector using 0.1 M lithium chloride (LiCl) in *N*,*N*‐dimethylacetamide (DMAc) as the eluent at a flow rate of 1.0 mL min^−1^. The system was calibrated against narrow‐distributed PMMA standards. All data were processed using PSS WinGPC UniChrom.

### Methods—Transmission Electron Microscopy (TEM)

For TEM measurements, 10 µL of a diluted NP suspension in ethanol were drop cast onto a copper grid 200 (meshes), coated with a carbon film. The copper grid was purchased from Science Services GmbH, and the copper film was deposited using physical vapor deposition at the University of Hamburg. Measurements were taken with a Tecnai G2 of the FEI Company operated at a voltage of 120 kV or a JEOL JEM 1011 operated at a voltage of 100 kV in the bright field mode. Images were taken with an Eagle 4K HS CCD camera. For size distributions, 300 nanoparticles were manually measured using ImageJ. The distribution curves were obtained by fitting the particle diameters in a log‐normal distribution using the software “OriginPro 2021” (version 9.8.0.200).

### Methods—Atomic Absorption Spectrometry

Approximately 10 mg of the sample were dissolved in 5 mL of aqua regia (HCl/HNO_3_, 3/1 (v/v)) using a microwave (Anton Paar Multiwave 7501, 250 °C, 943 W maximum power, 1 h) and diluted with water to a total volume of 15 mL.

The content of Au was determined using graphite furnace atomic absorption spectrometry (GF‐AAS) with the “AAnalyst 600” from Perkin Elmer as the average of a double determination.

### Methods—Ultraviolet–Visible (UV‐Vis) Spectroscopy

UV–vis extinction measurements were conducted with the UV5 spectrophotometer of Mettler Toledo in a quartz cuvette with a length of 1 cm at 293 K. In the spectra, the absorbance is plotted against the wavelength. The blank solvent was subtracted as a background for the colloidal systems. The same device with a custom‐made measurement cell (sample holder) was used to record the absorbance of thin vitrimer films (thickness < 1 mm). Four different spots on each film were measured, and the average was taken to show a representative spectrum of the film. The empty measurement cell was subtracted as the background for the film measurements.

### Methods—Dynamic Mechanical Analysis (DMA)

An MCR 502 rotational rheometer (Anton Paar GmbH) was used to perform oscillatory shear and static stress relaxation experiments. The gap between a Peltier plate and an 8 mm plate‐plate geometry was usually set to 1 mm and covered by a heat chamber with constant nitrogen gas to control the temperature. All of the samples were prepared as circular discs with a diameter of 8 mm and a thickness of 1 mm.

Prior to the oscillatory shear measurements, amplitude sweeps were performed at angular frequency *ω* of 10 rad s^−1^ and a normal force of 1 N at 25, 110, and 200 °C from shear strain *γ* of 0.001% to 10.0%. These tests confirmed that the selected strain amplitude *γ*_0_ was within the linear viscoelastic range (LVER), which resulted in strain independence of the storage modulus *G'* and the loss modulus *G”*. Moreover, frequency sweeps using a shear amplitude *γ* of 0.01% and a normal force of 1 N were recorded at an angular frequency *ω* from 1 to 100 rad s^−1^ at temperatures of 25, 110, and 200 °C. Temperature sweeps were performed at an angular frequency *ω* = 10 rad s^−1^ and a shear amplitude *γ* = 0.01%. Storage and loss moduli were recorded as functions of temperature ranging from 200 to 0 °C at cooling rates of 1 K min^−1^. In the case of stress relaxation experiments, relaxation moduli *G* were recorded as a function of time *t* with an initial step‐deformation *γ* of 0.1% at different temperatures *T* ranging from 110 to 160 °C. The relaxation times and activation energies were fitted to a stretched exponential decay (Equation 1).^[^ ^42^, ^43^ ^]^

(1) $$\frac{G}{G_{0}}=e^{-{\left(\frac{t}{\tau^{*}}\right)}^{\beta }}$$

$\tau^{*}\;$ is the relaxation time, $G_{0}$ is the initial relaxation modulus, and β is the relaxation distribution parameter (0 < β≤1). In the case of single relaxation (Maxwell model), β equals to 1. When β is less than 1, there is an overlay of relaxation modes with different rates. The average relaxation time <τ> can be calculated by (Equation 2);^[^ ^42^, ^43^ ^]^

(2) $$\langle \tau \rangle =\frac{\tau^{*}\;\Gamma \left(\frac{1}{\beta }\right)}{\beta }$$

where Γ is the gamma function. The ln < τ > is plotted against 1000/T, resulting in a linear relationship. The slope can be used to evaluate the activation energy *E*_a_ by (Equation 3);^[^ ^42^, ^43^ ^]^

(3) $$\langle \tau \rangle =\tau_{0}e^{\frac{E_{a}}{\mathrm{RT}}}$$

whereas *R* is the gas constant 8.314 J mol^−1^. All data were processed by RheoCompass software (version 1.19.266, Anton Paar GmbH).

### Methods—Differential Scanning Calorimetry (DSC)

A DSC 204 F1 Phoenix (NETZSCH Gerätebau GmbH) differential scanning calorimeter was used to determine the thermal properties of all vitrimer samples (≈5–10 mg) in an aluminum crucible under a nitrogen atmosphere. The thermograms cover temperatures between −50 and 160 °C at a heating rate of 10 K min^−1^. The second heating curve was interpreted by Proteus analysis (NETZSCH Gerätebau GmbH).

### Methods—Thermal Gravimetric Analysis (TGA)

The thermal stabilities of all vitrimer samples (≈5–10 mg) were measured in a ceramic crucible using a TGA 209 F1 Iris (NETZSCH Gerätebau GmbH) with a temperature range of 25–600 °C, a heating rate of 10 K min^−1^, and under nitrogen atmosphere. Subsequently, the data were processed by using Proteus analysis (NETZSCH Gerätebau GmbH).

### Methods—Material Preparation and Reprocessing

After drying in the oven, all vitrimer samples were placed between two Teflon sheets, then heated above their glass transition temperatures and pressed by a hydraulic press (Paul‐Otto Weber GmbH) at 150 °C into a thickness of 1 mm with a pressure of 10 kN and time of 2 min. To cut samples to the desired shape, they were heated above their glass transition temperature (150 °C) with a hot air gun (Steinel HL 2020 E) and then cut using a ZCP020 (Zwick Roell) for tensile testing and an 8 mm round hole punch tool for DMA. For reprocessing, small pieces of material were held between two Teflon sheets with the same parameters as described above.

### Methods—Tensile Testing

A universal testing machine (UTM) type Z020 (Zwick Roell) with a 5 kN load cell was used at room temperature (25 °C) and a relative humidity of 28–32% according to test standard DIN EN ISO 527‐1, initial load 0.1 MPa, specimen length 13.24 mm, initial rate of 1 mm min^−1^ between 0.05% and 0.25% elongation, and the rate of 10 mm min^−1^ above 0.25% elongation. All specimens were prepared with a hydraulic press at a thickness of 1 mm and cut into dog‐bone type specimens at a 2‐mm width according to ISO 527‐2.

### Methods—Photothermal Heating of the Colloidal Systems Depending on Laser Intensity

To investigate the photothermal heating efficiency, vitrimer films with different thicknesses and gold contents were positioned in the beam path of a 532 nm laser diode (finesse 532 pure, Laser Quantum). The laser was operated in continuous wave mode with a performance of 200 mW (5.4 W cm^−2^ intensity). The 1/e^2^ spot size is given by the manufacturer to be 3.2 mm^2^, assuming a Gaussian profile.

The films were illuminated for 2 min and the temperature change during the measurement was recorded with an Optris CTlaser LTF infrared thermometer. The distance between the sample and the thermometer was 70 mm, and the measurement interval was 9 ms.

### Methods—Self‐Healing Testing

One millimeter thick films were scratched with a cutter and healed by heating with a hot air gun (Steinel HL 2020 E) at 150 °C for 60 s. 3D images of surface materials were taken for damaged and healed surfaces using a confocal microscope VK‐X160K, Keyence (Japan), with an optical lens of 20x magnification. A two‐way light source (laser light and white light) was used in the microscope. A 3D‐profile of surface roughness can be constructed by detecting height information and light intensity. The depth and width of the scratch were analyzed by MultiFileAnalyzer.

### Methods—Ultrasonication

The heatable ultrasonic bath „Sonorex Digiplus DL514 BH″ from Bandelin Electronics was used at maximum amplitude to disperse NPs and assist with the dissolution of polymers.

### Methods—Swelling Ratio, Gel Fraction, and Soluble Fraction Testing

To determine the soluble fraction, pristine materials with an initial weight *W*_i_ of 40–60 mg were immersed in 5 mL THF for 24 h at room temperature. The remaining solution (THF and sol) was removed and then 3 mL THF was added. After 24 h, the solution was transferred to a glass vial with a glass dropper and dried to observe the remaining uncrosslinked part (sol). The process was then repeated 2 times until no sol was observed in the glass vial. Materials were dried in a vacuum oven at 100 °C to constant dried weight *W*_d_. The gel fraction [%] and soluble fraction [%] are *W*_d _*100/* W*_i_ and (*W*_i –_ *W*_d_) *100/* W*_i_, respectively. Materials (after removal of the sol fraction) with an initial weight *W*_i_ of 40–60 mg were soaked in water at room temperature to fully swollen state *W*_s_ (24 h). The swelling ratios *S* [%] were then calculated as *S* = (*W*_s –_ *W*_d_) *100/ *W*_d_.

### Methods—X‐Ray Photoelectron Spectroscopy (XPS)

For XPS measurements, 100 µL of aqueous NP suspensions were drop‐cast on a silicon wafer and immobilized on a 60 mm XPS sample holder using double‐sided carbon tape. The measurements were carried out using the PHI VersaProbe III equipped with Al 1486.6 eV mono X‐ray source at 24.8 W resulting in a beam diameter of 100 µm. The pass energy for survey spectra was 224.0 eV with an increment of 0.2 eV and 0.2 s per data point. The pass energy for high‐resolution measurements was 27.0 eV in increments of 0.05 eV and 2.4 s per data point. All spectra were charge‐corrected according to advantageous carbon binding energy at 284.8 eV with a charge neutralizer (1 V and 3 µA). The signals in the high‐resolution spectra were fitted using MultiPak software (version 9.8). The background was fitted using a Shirley function for all elements. The elemental spectra of N 1s, S 2p, and C 1s were fitted with a Gaussian‐Lorentzian function, and the Au 4f peaks were fitted with an asymmetric Gaussian‐Lorentzian function.

### Methods—Nanoindentation

The nanoindentation tests are performed in an iMicro Indentation System OBID‐1276‐1 (Nanomechanics, Inc., USA) at room temperature, with a diamond Berkovich tip (Synton‐MDP, Switzerland) and aluminum specimen using the InView RunTest software and method “Advanced Dynamics E and H”. This method utilizes the iMicro Dynamics option and a constant strain rate to perform an indentation to a desired load or depth. Moreover, it can control the experiment through several inputs, affecting the frequency of oscillation, dynamic displacement, *etc*. The following values are used: Poisson ratio 0.35, target load 1000 mN, target depth 2000 nm, surface approach distance 2000 nm, surface approach velocity 100 nm s^−1^, strain rate 0.2 s^−1^, max load time 1 s, harmonic displacement 2.0 nm, frequency target 110.0 Hz. The evaluation of the measurement was performed by InView Review software.

### Methods—Statistical Analysis

All the statistical analyzes were performed by using OriginPro 2023b (10.0.5.153). One way Analysis of Variance (ANOVA) was used to evaluate significant differences with Tukey's Range Test to calculate significant mean differences which accepted the 0.05 probability as significant.

## Conflict of Interest

The authors declare no conflict of interest.

## Author Contributions

P.S. and S.W. contributed equally to this work. The experiments were designed and executed by P.S. and S.W., under the supervision of B.H. and V.A. C.J. assisted in the execution of the experiments. The manuscript was written, edited, and reviewed through the contributions of all authors. All authors have given approval to the final version of the manuscript.

## Supporting information



Supporting Information

Supplemental Video 1

Supplemental Video 2

Supplemental Video 3

Supplemental Video 4

## Data Availability

The data that support the findings of this study are available in the supplementary material of this article.
