# Supplementary material for: Nanocomposites from Au‐Doped Vinylogous Urethane Vitrimers Based on Different Block Copolymers and Their Recyclability in Combination with Plasmonic Heating
Source: Macromol Rapid Commun. 2025 Mar 6;46(12):2401027. doi: 10.1002/marc.202401027 (PMC12183147; doi:10.1002/marc.202401027)
Supplement: Supplementary file 1 — Supporting Information [file MARC-46-2401027-s005.pdf]

Macromolecular Rapid Communications

Supporting Information

for *Macromol. Rapid Commun.*, DOI 10.1002/marc.202401027

Nanocomposites from Au-Doped Vinylogous Urethane Vitrimers Based on Different Block Copolymers and Their Recyclability in Combination with Plasmonic Heating

Patrick Schütz, Siraphat Weerathaworn, Clas Jürgensen, Birgit Hankiewicz and Volker Abetz**

Nanocomposites from Au-doped vinylogous urethane vitrimers based on different block copolymers and their recyclability in combination with plasmonic heating

*Patrick Schütz, Siraphat Weerathaworn, Clas Jürgensen, Birgit Hankiewicz**, and Volker Abetz**

E-Mail: birgit.hankiewicz@uni-hamburg.de, volker.abetz@uni-hamburg.de

Contents

S1 Determination of the Monomer Conversion

S2 Structural Confirmation of the Polymers and Nanocomposites

S3 Characterization of the Gold Nanoparticles (Au-NPs)

S4 Alternative Synthesis Route 1

S5 Optical Properties of the Nanocomposites Obtained *via* Synthesis Route 2

S6 X-ray Photoelectron Spectroscopy (XPS)

S7 Swelling Properties of the Nanocomposites

S8 Thermogravimetric Analysis (TGA)

S9 Differential Scanning Calorimeter (DSC)

S10 Temperature Sweeps

S11 Rheological Properties

S12 Reprocessing, Reshaping, Self-healing, and Shape-memory

S13 Investigation of Plasmonic Heating of the Nanocomposites

S1 Determination of Monomer Conversion

The conversion (α) of monomers in photoRAFT was calculated based on the decline of the vinyl ^{1}H NMR signal at the end of the reaction (M_i+ M'_i) compared to before polymerization (M_0 + M'_0).

$$\alpha = \left(1 - \frac{I_{\mathrm{M}_i} + I_{\mathrm{M}_i'}}{I_{\mathrm{M}_0} + I_{\mathrm{M}_0'}}\right) \cdot 100\% \qquad \text{(S1)}$$

I_{M_0} and $I_{\mathrm{M}_0'}$ are the signal integrals at the beginning of the polymerization, whereas I_{M_i} and $I_{\mathrm{M}_i'}$ are those at the end. DMF is used as an internal standard for NMR integration. During the polymerization, the signal of DMF does not change.

S2 Structural Confirmation of the Polymers and Nanocomposites

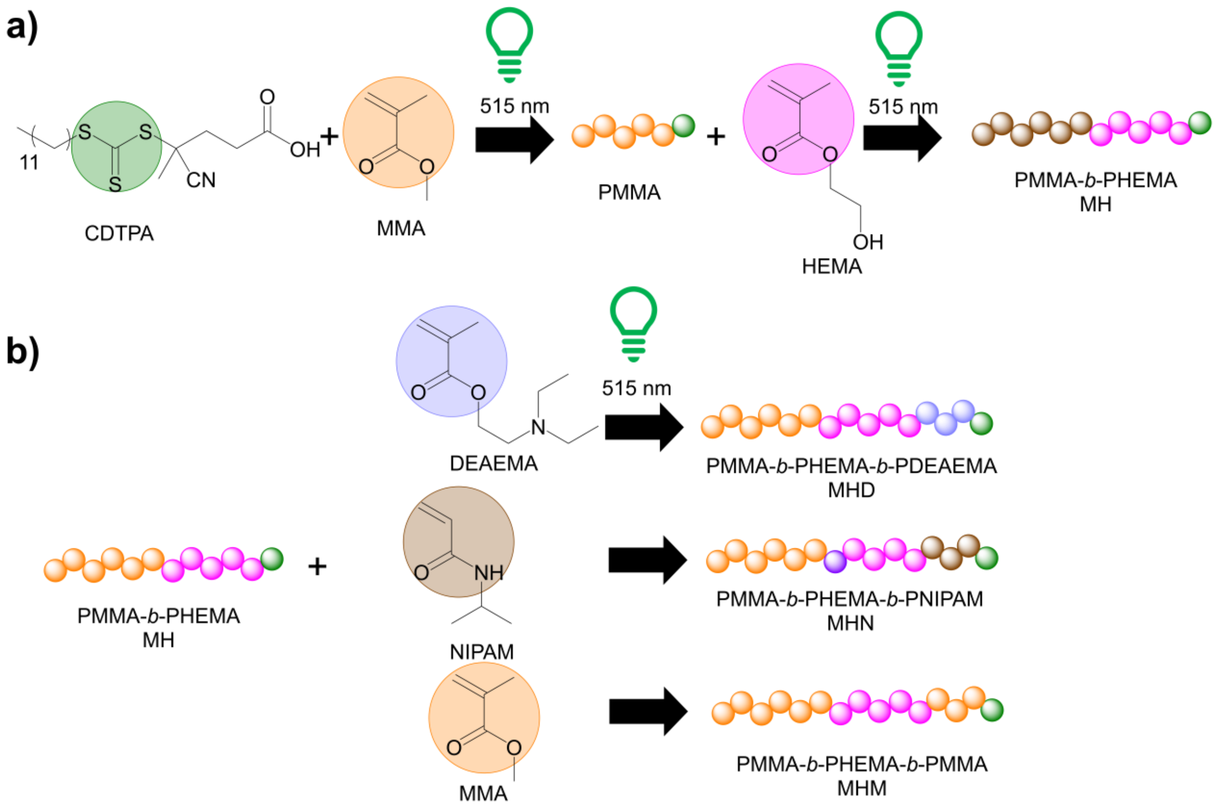

Figure S1: Synthetic route of **a)** the diblock copolymer PMMA-*b*-PHEMA and **b)** the three different triblock copolymers PMMA-*b*-PHEMA-PDEAEMA, PMMA-*b*-PHEMA-*b*-PNIPAM and PMMA-*b*-PHEMA-*b*-PMMA using photoiniferter RAFT-polymerization.

Table S1: Chemical characteristics of di- and triblock copolymers.

Samples (Sample Abbreviation)	Molar ratio$_{syn}$ [-]	Monomer conversion [%]	%wt [a] [%]	$\overline{M}_{n,NMR}$ [b] [kDa]	$\overline{M}_{n,SEC}$ [c] [kDa]	$Đ$ [d] [-]
PMMA-*b*-PHEMA (MH)	CDTPA/MMA 1/105	60	42	6.3	11.2	1.45
	PMMA/HEMA 1/1.75	61	58	16.1		
PMMA-*b*-PHEMA-*b*-PDEAEMA (MHD)	CDTPA/MMA 1/124	50	34	6.2	11.7	1.83
	PMMA/HEMA 1/1.80	63	57	15.4		
	PMMA-*b*-PHEMA /DEAEMA 1/19	54	9	16.7		
PMMA-*b*-PHEMA-*b*-PMMA (MHM)	CDTPA/MMA 1/135	44	36	5.9	10.8	1.56
	PMMA/HEMA 1/2.22	55	58	15.3		
	PMMA-*b*-PHEMA /MMA 1/19	31	6	16.4		
PMMA-*b*-PHEMA-*b*-PNIPAM (MHN)	CDTPA/MMA 1/135	44	34	5.9	11.3	1.63
	PMMA/HEMA 1/2.22	55	57	15.3		
	PMMA-*b*-PHEMA /NIPAM 1/19	50	9	16.9		

[a]weight percent composition of each block of the final product; [b]number average molecular weight determined by ^{1}H NMR spectra $\overline{M}_{n,NMR}$ of the purified backbones according to RAFT end group after each polymerization step; [c,d] number average molecular weight determined by SEC $\overline{M}_{n,SEC}$ and dispersity $Đ$ of final polymers with DMAc as an eluent and narrowly distributed PMMA as standard.

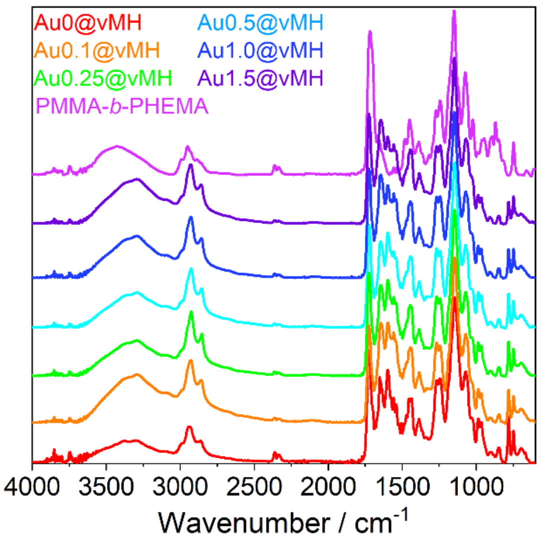

Figure S2: ATR-FTIR spectra of diblock copolymer and diblock copolymer vitrimers with various amounts of Au-NPs, *i.e.*, 0, 0.1, 0.25, 0.5, 1.0, and 1.5 wt%.

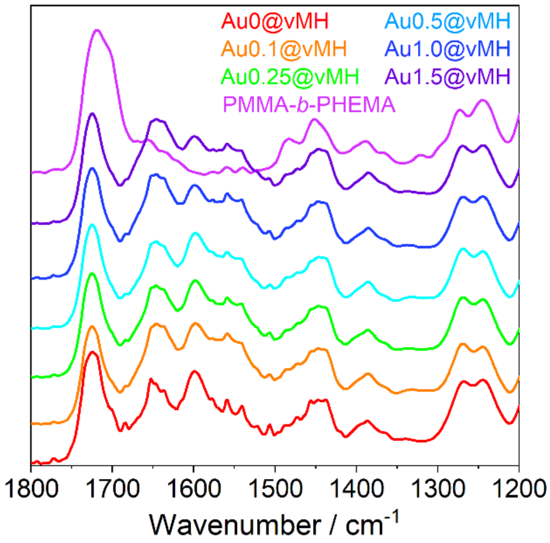

Figure S3: Enlarged ATR-FTIR spectra of diblock copolymer and diblock copolymer vitrimers with various amounts of Au-NPs, *i.e.*, 0, 0.1, 0.25, 0.5, 1.0, and 1.5 wt%.

S3 Characterization of the gold nanoparticles (Au-NPs)

To observe if the Au@Citrate-NPs' morphology and optical properties change significantly during the procedure preceding the ligand exchange, TEM and UV–Vis-spectroscopy were performed as shown in **Figure S4** and **Figure S5**.

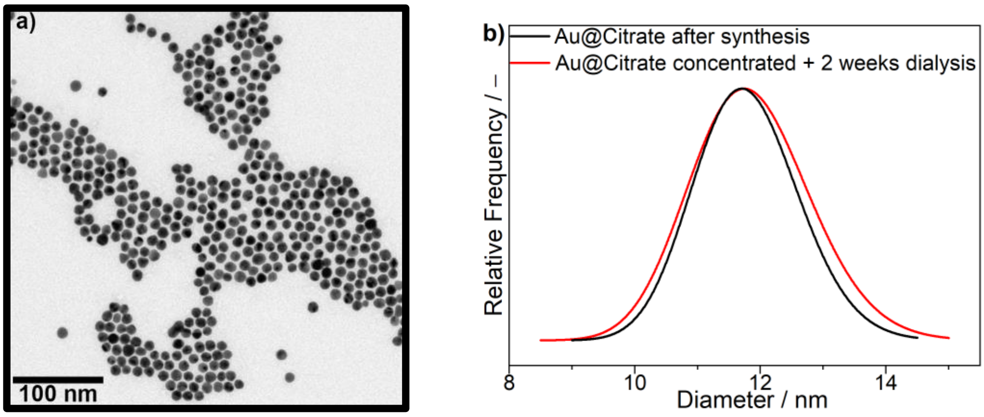

Figure S4: (a) Representative TEM image of the as-synthesized Au@Citrate-NPs and **(b)** the size distribution curves of the as-synthesized Au@Citrate-NPs (d = 11.8±0.8 nm, black) and the same Au@Citrate-NPs after the NP concentration was tripled *via* distillation and the particles were dialyzed for 2 weeks (d =11.8±0.9 nm, red).

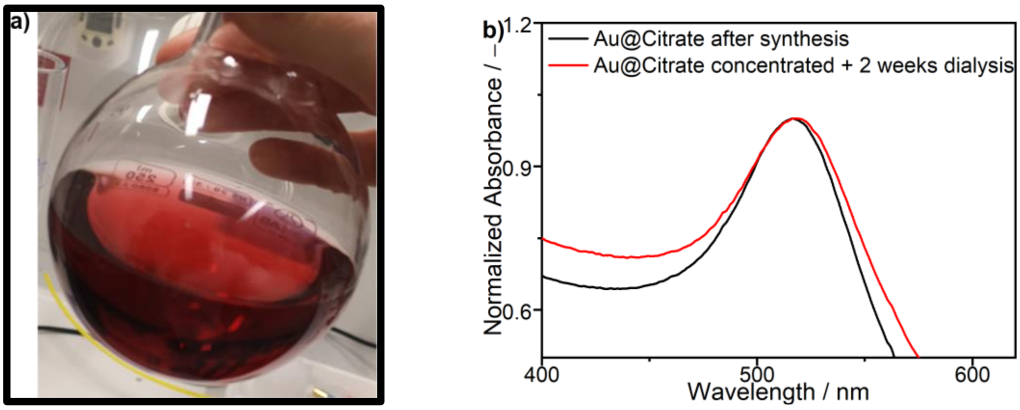

Figure S5: (a) Photograph of a diluted Au@Citrate-NP suspension and **(b)** the normalized UV–Vis-extinction spectra of the diluted as synthesized Au@Citrate-NPs (black) and the Au@Citrate-NPs after the NP concentration was tripled *via* distillation and 2 weeks of dialysis against water (red).

S4 Alternative Synthesis Route 1

In **Figure 1** in the main article, two different synthesis routes are presented to obtain the nanocomposites. In the following, synthesis route 1 will be discussed in more detail.

To functionalize the Au@Citrate-NPs in aqueous suspension with the different BCPs they need to be solved in the same medium. Since the phase transfer of the Au@Citrate-NPs without using another ligand has a high likelihood of causing irreversible aggregation of the NPs, we first tried to solve the BCPs in water. We found that even the more polar BCPs PMMA-*b*-PHEMA-*b*-PNIPAM and PMMA-*b*-PHEMA-*b*-PDEAEMA were not soluble in water. After trying different mixtures of water and polymer solutions in methanol, we found that the particles and polymer can be mixed by adding an equal volume of the polymer solution to the aqueous Au@Citrate-NP suspension. After 1 h of vigorous stirring, 1 h of ultrasonication, and 22 h of stirring at room temperature, the ligand exchange is completed, and a clear purple colloidal solution is obtained. After methanol was removed *via* distillation, we could observe the formation of a turbid violet dispersion, showing that the Au@MHD-NPs are not well soluble in pure water, which indicates that the ligand exchange was successful (**Figure S6a**). The Au@MHD-NPs could then be isolated by centrifugation (8000 rcf, 60 min) or by removing water *via* distillation. The precipitated Au@MHD-NPs can be dissolved in organic solvents like DMF or THF, forming a clear purple suspension for further procedures while preserving the optical properties of the Au-NPs (**Figure S6b**). This further indicates that the functionalization procedure was successful since the Au@Citrate-NPs could not be dispersed in organic solvents without aggregation of the Au-NPs before the ligand exchange. The acetoacetylation of the Au@MHD-NPs can be performed following the same procedure as for the pure BCPs. After the reaction was finished, DMF was removed *via* distillation, and the Au@MHD-AcAc-NPs dissolved in THF for the crosslinking procedure, leaving a blue clear suspension. The change in color of the suspension after the acetoacetylation suggests that the particle changed either in size or aggregated during the procedure, which was investigated using TEM and UV–Vis-spectroscopy (**Figure S6c, d**). The investigation revealed that the Au-NPs formed chain-like oligomers during the reaction, which can also be observed as a shoulder in the extinction spectrum similar to gold nanorods or gold nanocorals.[1] The same optical properties can also be observed when the Au@MHD-AcAc-NPs are crosslinked to form the Au@vMHD films (**Figure S6d**). Due to this chain formation, which leads to a change in the optical properties, we chose synthesis route 2 over synthesis route 1 to obtain the Au@vBCP-films with a less drastic change of the optical properties of the initial Au@Citrate-NPs as shown in **Figure S6d**.

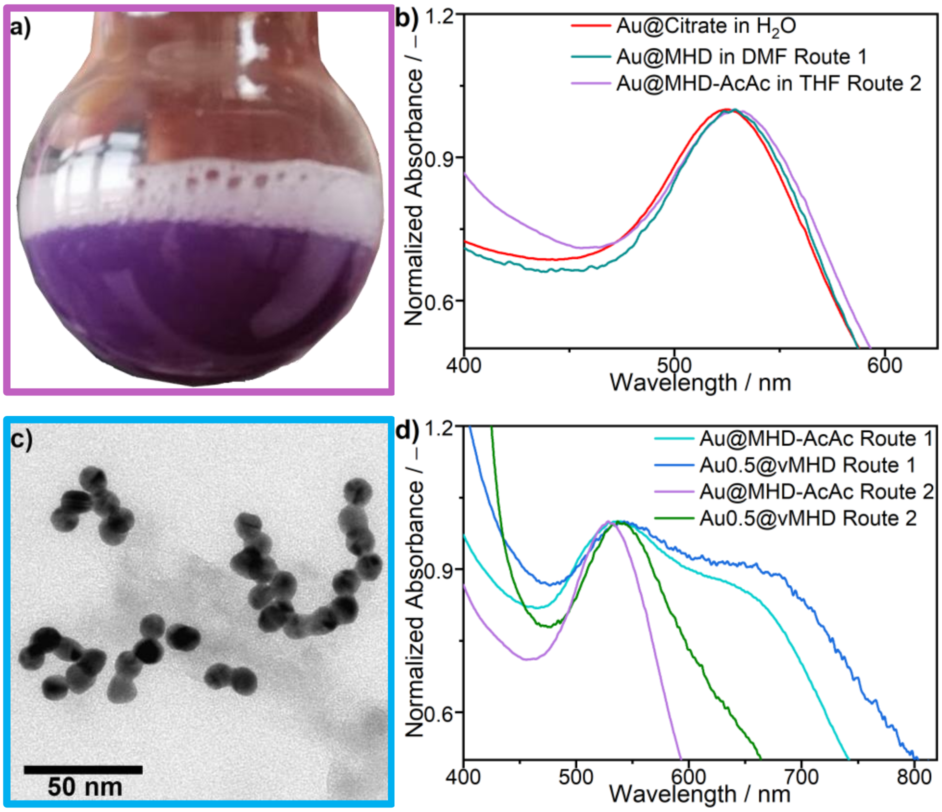

Figure S6: (a) Photo of the Au@MHD-AcAc in water after removing the THF from the reaction mixture. **(b)** Normalized UV–Vis-extinction spectra of the initial Au@Citrate-NPs in water (red), the Au@MHD-NPs in DMF obtained in synthesis route 1 (light green) and Au@MHD-AcAc-NPs in THF obtained in synthesis route 2 (violet). **(c)** A representative TEM-image of the Au@BCP-AcAc-NPs that were prepared using Au@Citrate-NPs (**Figure S4**) following synthesis route 1. **(d)** Normalized UV–Vis-extinction spectra of Au@MHD-AcAc-NPs in THF and the Au@vMHD film obtained by following synthesis route 1 (cyan and blue) and synthesis route 2 (violet and green).

S5 Optical Properties of the Nanocomposites Obtained *via* Synthesis Route 2

The study showed that the nanocomposites' optical properties depend on multiple parameters that influence how the NPs are dispersed within the materials. When comparing multiple materials synthesized using multiple NP-batches and different polymers, we could observe that sometimes the NPs form macroscopic aggregates during the transfer to the organic solvent or the crosslinking procedure (**Figure S7**).

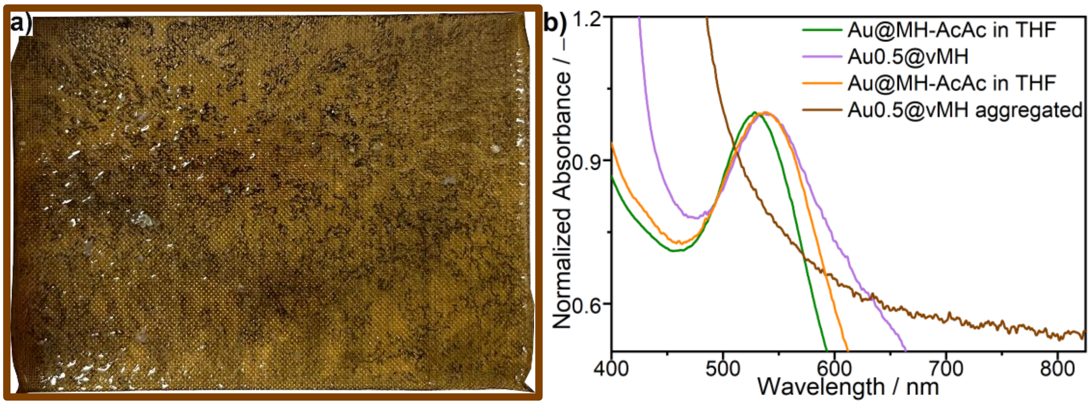

Figure S7: (a) Photograph of an Au0.5@vMH-film that showed macroscopic aggregation of the Au-NPs. **(b)** The normalized UV–Vis extinction spectrum of Au@MH-AcAc-NPs in THF (green) that formed an Au0.5@vMH film with well-dispersed Au-NPs (violet), and Au@MH-AcAc-NPs in THF (orange) that formed an Au@vMH-film with aggregated Au-NPs (brown).

This behavior took place while seemingly not changing the procedure and was dependent on the NP batch that was used but could not be predicted by the TEM images or optical properties of the Au@Citrate-NPs. We concluded that the post-synthesis treatment of the Au@Citrate-NPs, which determines the ionic strength or the NP concentration of the colloid, is crucial to the properties of the final product. We investigated this by changing the ionic strength of the initial Au@Citrate-NP colloid, showing that an increase in ionic strength leads to aggregation and a broadening of the surface plasmon resonance (**Figure S8a**). When the ionic strength in the colloid is even higher, aggregates of the Au-NPs in the material start to form.

The different BCPs (PMMA-*b*-PHEMA, PMMA-*b*-PHEMA-*b*-PMMA, PMMA-*b*-PHEMA-*b*-PNIPAM, and PMMA-*b*-PHEMA-*b*-PDEAEMA) could be used to form Au0.5@vBCP-films (**Figure S8b**). We could observe that it is possible for all four polymer structures, but the optical properties depend on the Au-NP batch that was used. this is shown by nearly identical extinction spectra for the films containing PMMA-*b*-PHEMA-*b*-PMMA and PMMA-*b*-PHEMA-*b*-PNIPAM, even though they have very different functional groups and hydrophilicity.

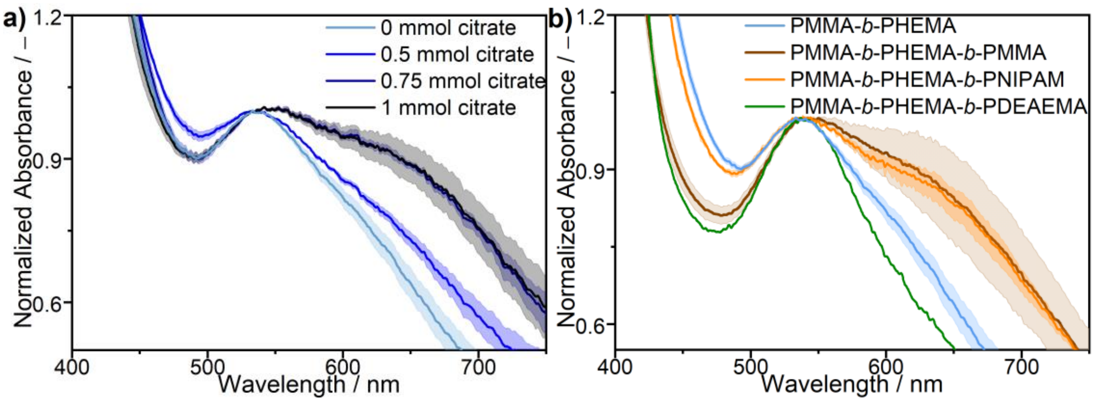

Figure S8: (a) The normalized UV–Vis extinction spectra of Au0.5@vMH-films using an Au@Citrate-NP colloid that was dialyzed against aqueous trisodium citrate solutions with different concentrations or water for two weeks (0 mmol citrate). The Au@Citrate-NPs were synthesized in the same batch. **(b)** The normalized UV–Vis extinction spectra of Au0.5@vBCP films using different BCP structures. The Au0.5@vMHM and Au0.5@vMHN films were synthesized using the same NP batch.

To investigate the reproducibility dependent on the Au-NP batch that was used to synthesize Au@vBCP films, the optical properties of three Au0.5@vMH films and one Au0.5@vMHN film using Au-NPs from four different batches were compared (**Figure S9a**). We can observe that three out of four films using different Au-NP batches showed an absorption maximum in the area of 533–546 nm and a good overlap of the spectrum even though the orange curves show a significant broadening, such as the magenta curve. The broadening of the surface plasmon resonance of Au-NPs is generally connected to the growth of the particles or the formation of clusters, which could be induced by high ionic strength, as discussed before, but also by aging of the Au-NPs. Using TEM, the size of the Au-NPs was evaluated in a 6-month period after dialysis for 2 weeks, showing no significant change in the particle size distribution. This makes it likely that the change is connected to the aggregation of the NPs. After dialysis of the as-synthesized Au-NPs, the presence of ligands in the medium is drastically reduced. While this procedure aids the ligand exchange reaction, it also reduces the long-term colloidal stability of Au-NPs. Due to the reduced amount of ligands, the NPs are more likely to aggregate over time to minimize their surface energy. The effect of aging is investigated by comparing the optical properties of two Au0.5@vMH films that were synthesized using the same Au-NP batch right after the dialysis of Au-NPs and after a two-month period (**Figure S9b**). Here, we also observe a broadening connected to the particles being stored for an extended time before use.

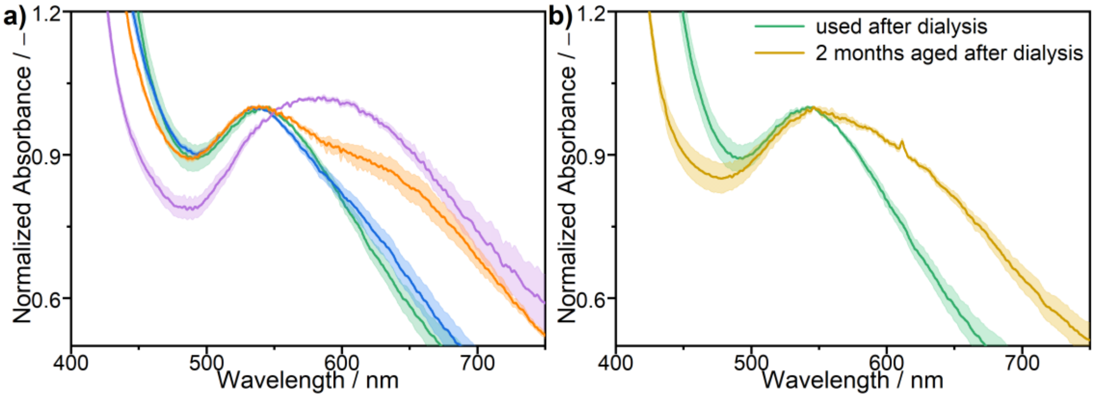

Figure S9: (a) The normalized UV–Vis extinction spectra of Au0.5@vMH-films (blue, green, and magenta) and Au0.5@vMHN (orange) using Au-NPs synthesized in different batches. **(b)** The normalized UV–Vis extinction spectra of Au0.5@vMH films using Au-NPs right after 2 weeks of dialysis (green) and after an additional 2 months of aging after 2 weeks of dialysis (orange). The films were synthesized using the same Au-NP batch.

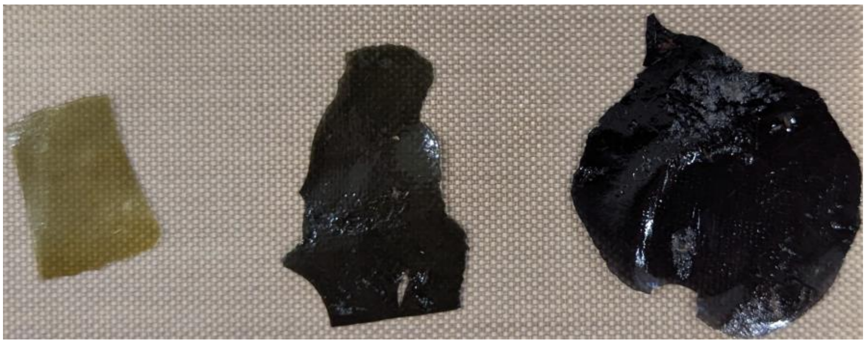

Figure S10: Photograph of 0.2 mm thick Au@vMH-films with different gold contents of 0.1 wt% (left), 0.25 wt% (middle) and 0.5 wt% (right).

Table S2: The theoretically possible gold content (sum of the masses of polymer, Au-NPs and crosslinker) of different Au@vBCP films, the amounts measured with GF-AAS (double determination), and the integration efficiency of gold given by the ratio of the theoretical content and the measured content.

Sample (Au:Polymer)	Theoretical gold content [wt%]	Measured gold content [wt%]	Integration efficiency [%]
Au0.1@vMH	0.08	0.05±0.01	63
Au0.25@vMH	0.20	0.14±0.01	65
Au0.5@vMH	0.40	0.36±0.01	93
Au1.0@vMH	0.79.	0.75±0.01	95
Au1.5@vMH	1.19	0.92±0.01	78
Au1.0@vMHD	0.80	0.71±0.01	89
Au0.5@vMHN	0.40	0.33±0.01	82
Au0.5@vMHM	0.40	0.49±0.01	123

S6 X-ray photoelectron spectroscopy (XPS)

To investigate the presence of the polymer and gain insights on the binding state, XPS was performed with the Au@MH-NPs and Au1.5@vMH films. The high-resolution spectra of Au@MH-NPs in **Figure S6.1a** show the C 1s peaks of C=O at 288.9 eV, C–O at 286.6 eV, and C–C/C–H at 284.8 eV connected to the functional groups of the polymer chains.[2–4] The characteristic doublet of Au $4f_{7/2}$ and Au $4f_{5/2}$ was detected at 87.5 eV and 83.8 eV, confirming the NPs' Au^0 oxidation state.[5] The Au–S linkage of the TTC group can be seen in the S 2p peak at 163.4 eV, which aligns with previously shown binding energies of 162.5–163.6 eV for TTC-terminated polymers on gold surfaces.[6] The broad S 2p peak around 168.8 eV shows sulfur, which is either unbound or oxidized due to X-ray irradiation.[7] The measurements verify the presence of the polymer on the Au-NPs' surface with the S 2p peak, which suggests the chemisorption of the TTC group to the gold surface.

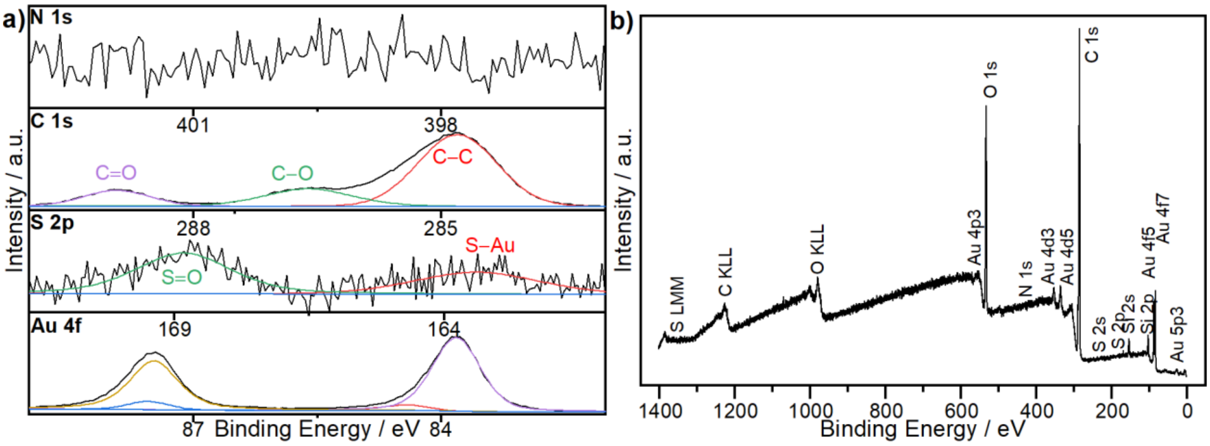

Figure S11: (a) High-resolution XPS spectra of N 1s, C 1s, S 2p, and Au 4f of Au@MH-NPs. The measured spectrum is shown in black, and the background in blue. Peaks of different binding states are fitted onto the curve. **(b)** The XPS survey spectrum of Au@MH-NPs.

The survey spectrum shows the BCP's elements C, O, and S as well as Au from the NPs (**Figure S11b**). No significant impurities were detected in the sample.

The chemical composition of the surface of an Au1.5@vMH-film was also investigated with XPS in **Figure S12**. The survey spectrum shows the vitrimers's elements C, O, N, and S, as well as Au from the NPs. (**Figure S12b**) No significant impurities were detected in the sample. The high-resolution spectra show the C 1s peaks of C=O at 288.9 eV, C–O at 286.4 eV, and C–C/C–H at 284.7 eV connected to the functional groups of the polymer chains.[2–4] The characteristic doublet of Au $4f_{7/2}$ and Au $4f_{5/2}$ was lower in intensity than the Au@MH-NPs due

to the lower volume concentration of Au in the sample. The doublet of Au $4f_{7/2}$ and Au $4f_{5/2}$ was detected at 87.0 eV and 83.5 eV, confirming the conservation of the Au^0 oxidation state. (**Figure S12a**).[5] The N 1s peak, which could not be seen in the Au@MH-NPs, can be detected at 399.5 eV in the vitrimer due to the amines that form the dynamic network.[8] The S 2p peak of the TTC group could not be detected in the spectrum due to the lower volume concentration of bound sulfur compared to the Au@MH-NPs. Therefore, the measurement verifies the presence of Au-NPs within the nanocomposites.

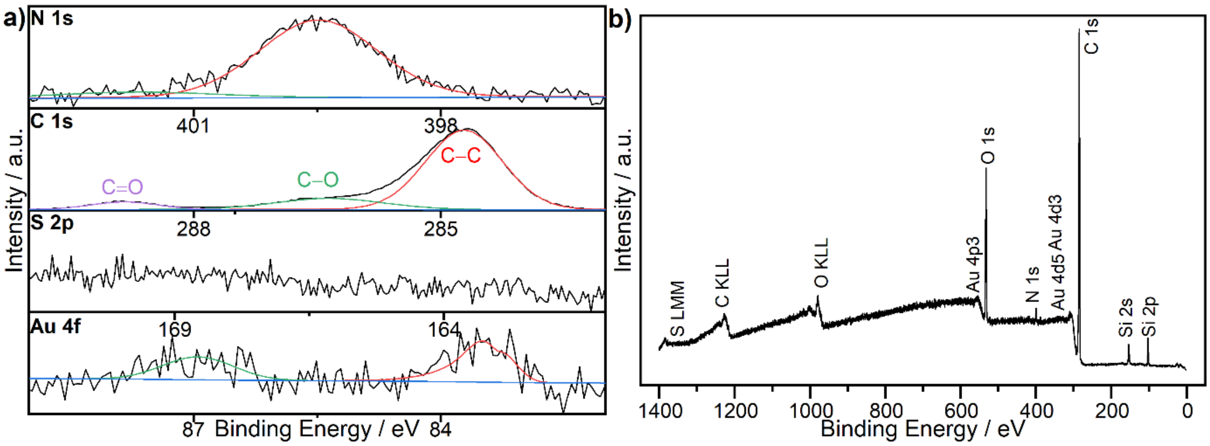

Figure S12: (a) High-resolution XPS spectra of N 1s, C 1s, S 2p, and Au 4f of Au1.5@vMH. The measured spectrum is shown in black, and the background in blue. Peaks of different binding states are fitted onto the curve. **(b)** The XPS survey spectrum of Au1.5@vMH.

S7 Swelling Properties of the Nanocomposites

Table S3: Swelling ratio in water, gel fraction in THF, and soluble fraction in THF of materials.

Samples	Swelling ratio in water [wt%]	Gel fraction in THF [wt%]	Soluble fraction in THF [wt%]
Au0@vMH	88.5	90.0	10.0
Au0.1@vMH	86.9	90.1	9.9
Au0.25@vMH	85.9	90.9	9.1
Au0.5@vMH	72.9	91.0	9.0
Au1.0@vMH	71.9	93.0	7.0
Au1.5@vMH	66.1	93.2	6.8
Au0.5@vMHM	73.2	93.2	6.8
Au0.5@vMHD	85.3	93.0	7.0
Au0.5@vMHN	91.2	93.5	6.5

S8 Thermogravimetric Analysis (TGA)

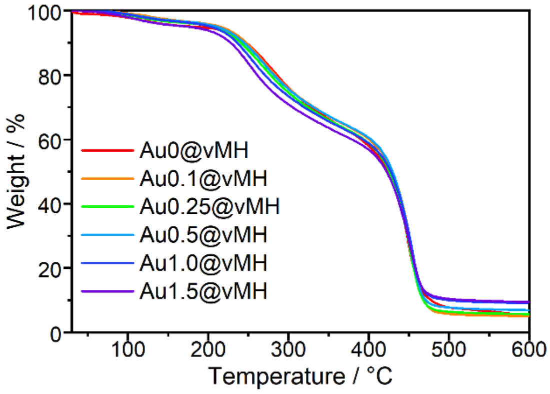

Figure S13: TGA thermograms of Au@vMH vitrimers with various amounts of Au-NPs, *i.e.*, 0, 0.1, 0.25, 0.5, 1.0, and 1.5 wt%. The temperature ranges from 25 to 600 °C with a heating rate of 10 °C min^{-1} and under the nitrogen atmosphere.

S9 Differential Scanning Calorimetry (DSC)

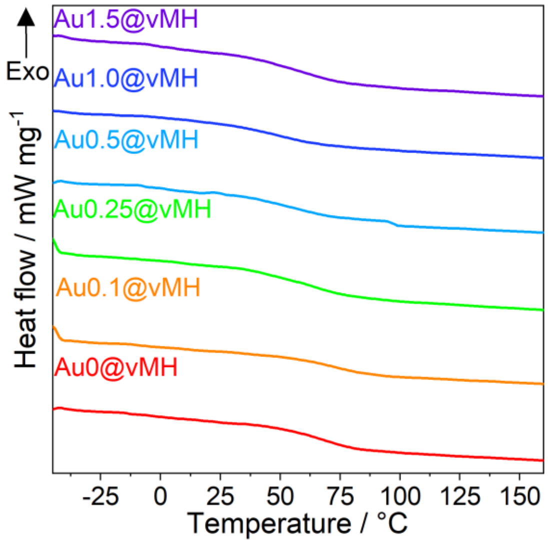

Figure S14: DSC thermograms (second heating curves) of Au@vMH vitrimers with various amounts of Au-NPs, *i.e.*, 0, 0.1, 0.25, 0.5, 1.0, and 1.5 wt%. The temperature ranges from −45 to 160 °C with a heating rate of 10 °C min^{-1}.

S10 Dynamic Mechanical Analysis: Temperature Sweeps

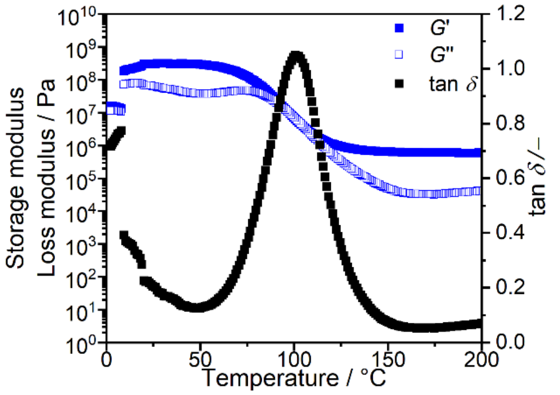

Figure S15: Temperature sweep of Au0@vMH showing storage moduli G' (filled blue symbol), loss moduli G'' (open blue symbol), and tan δ (filled black symbol) ranging from 200 to 0 °C with angular frequency ω = 10 rad s^{-1} and deformation γ = 0.01%.

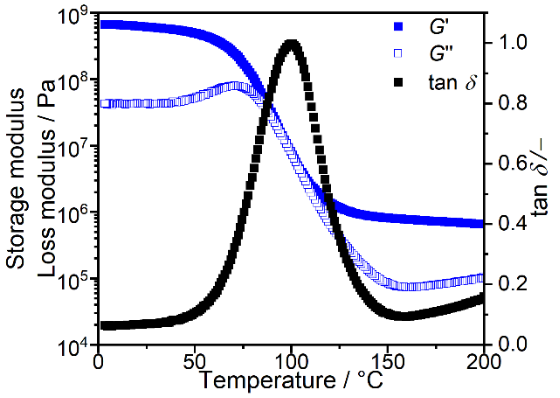

Figure S16: Temperature sweep of Au0.1@vMH showing storage moduli G' (filled blue symbol), loss moduli G'' (open blue symbol), and tan δ (filled black symbol) ranging from 200 to 0 °C with angular frequency ω = 10 rad s^{-1} and deformation γ = 0.01%.

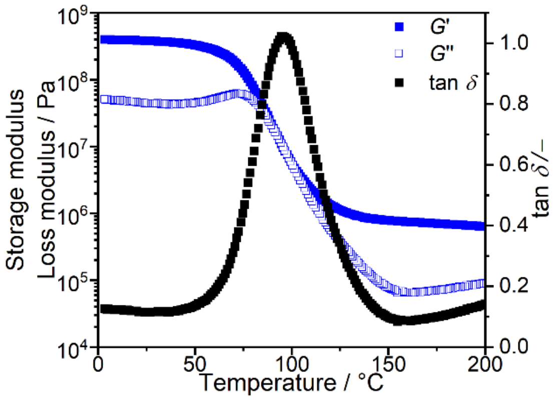

Figure S17: Temperature sweep of Au0.25@vMH showing storage moduli *G′* (filled blue symbol), loss moduli *G″* (open blue symbol), and tan *δ* (filled black symbol) ranging from 200 to 0 °C with angular frequency ω = 10 rad s^{-1} and deformation γ = 0.01%.

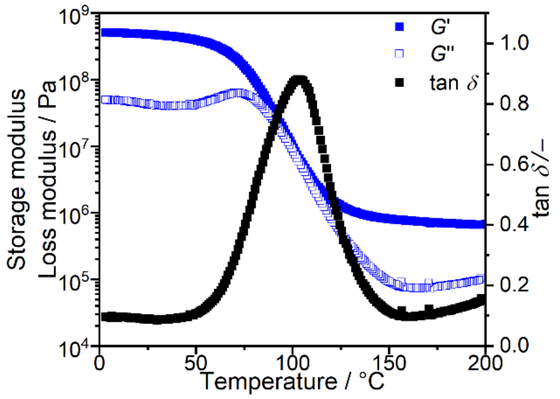

Figure S18: Temperature sweep of Au0.5@vMH showing storage moduli *G′* (filled blue symbol), loss moduli *G″* (open blue symbol), and tan *δ* (filled black symbol) ranging from 200 to 0 °C with angular frequency ω = 10 rad s^{-1} and deformation γ = 0.01%.

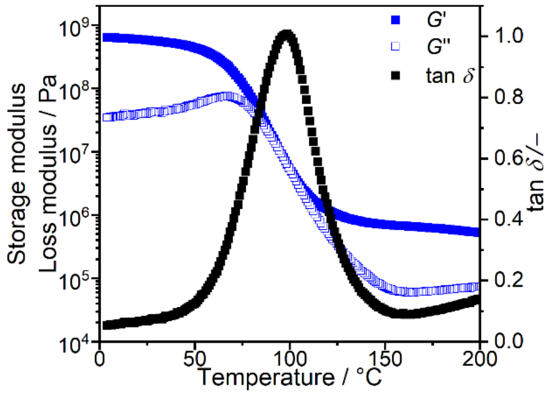

Figure S19: Temperature sweep of Au1.0@vMH showing storage moduli G' (filled blue symbol), loss moduli G'' (open blue symbol), and tan δ (filled black symbol) ranging from 200 to 0 °C with angular frequency ω = 10 rad s^{-1} and deformation γ = 0.01%.

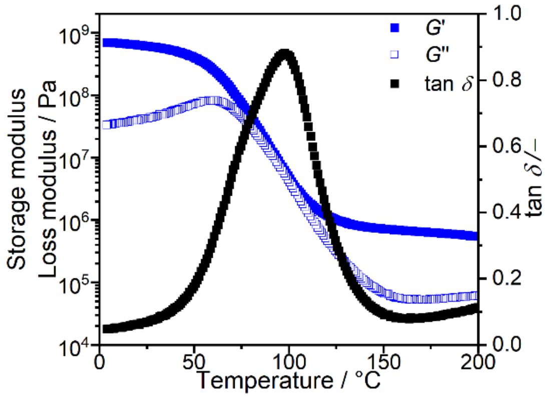

Figure S20: Temperature sweep of Au1.5@vMH showing storage moduli G' (filled blue symbol), loss moduli G'' (open blue symbol), and tan δ (filled black symbol) ranging from 200 to 0 °C with angular frequency ω = 10 rad s^{-1} and deformation γ = 0.01%.

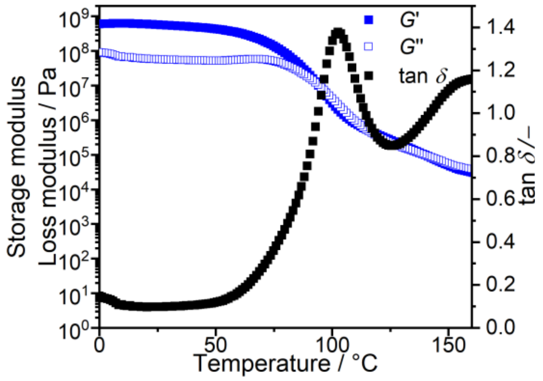

Figure S21: Temperature sweep of a PMMA homopolymer showing storage moduli G' (filled blue symbol), loss moduli G'' (open blue symbol), and tan δ (filled black symbol) ranging from 160 to 0 °C with angular frequency ω = 10 rad s^{-1} and deformation γ = 0.01%.

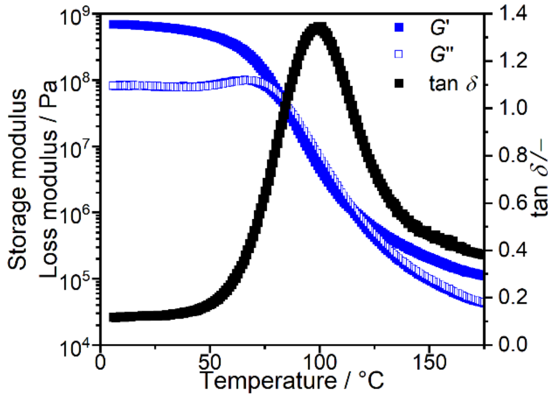

Figure S22: Temperature sweep of Au0.5@vMHM showing storage moduli G' (filled blue symbol), loss moduli G'' (open blue symbol), and tan δ (filled black symbol) ranging from 200 to 0 °C with angular frequency ω = 10 rad s^{-1} and deformation γ = 0.01%.

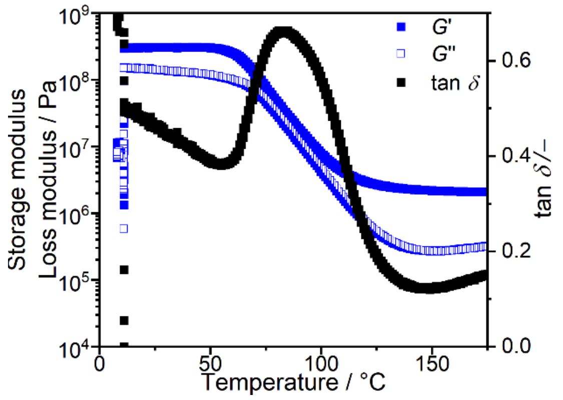

Figure S23: Temperature sweep of Au0.5@vMHD showing storage moduli G' (filled blue symbol), loss moduli G'' (open blue symbol), and tan δ (filled black symbol) ranging from 200 to 0 °C with angular frequency ω = 10 rad s^{-1} and deformation γ = 0.01%.

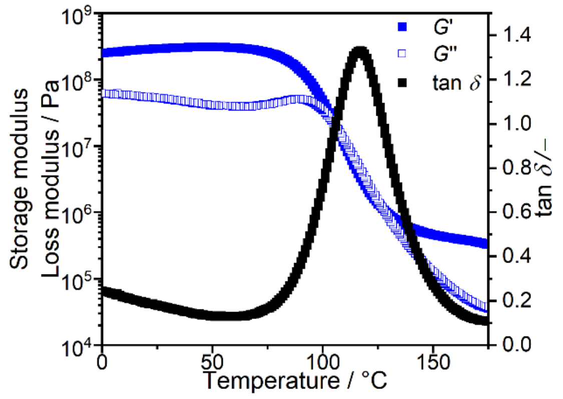

Figure S24: Temperature sweep of Au0.5@vMHN showing storage moduli G' (filled blue symbol), loss moduli G'' (open blue symbol), and tan δ (filled black symbol) ranging from 200 to 0 °C with angular frequency ω = 10 rad s^{-1} and deformation γ = 0.01%.

S11 Rheological Properties

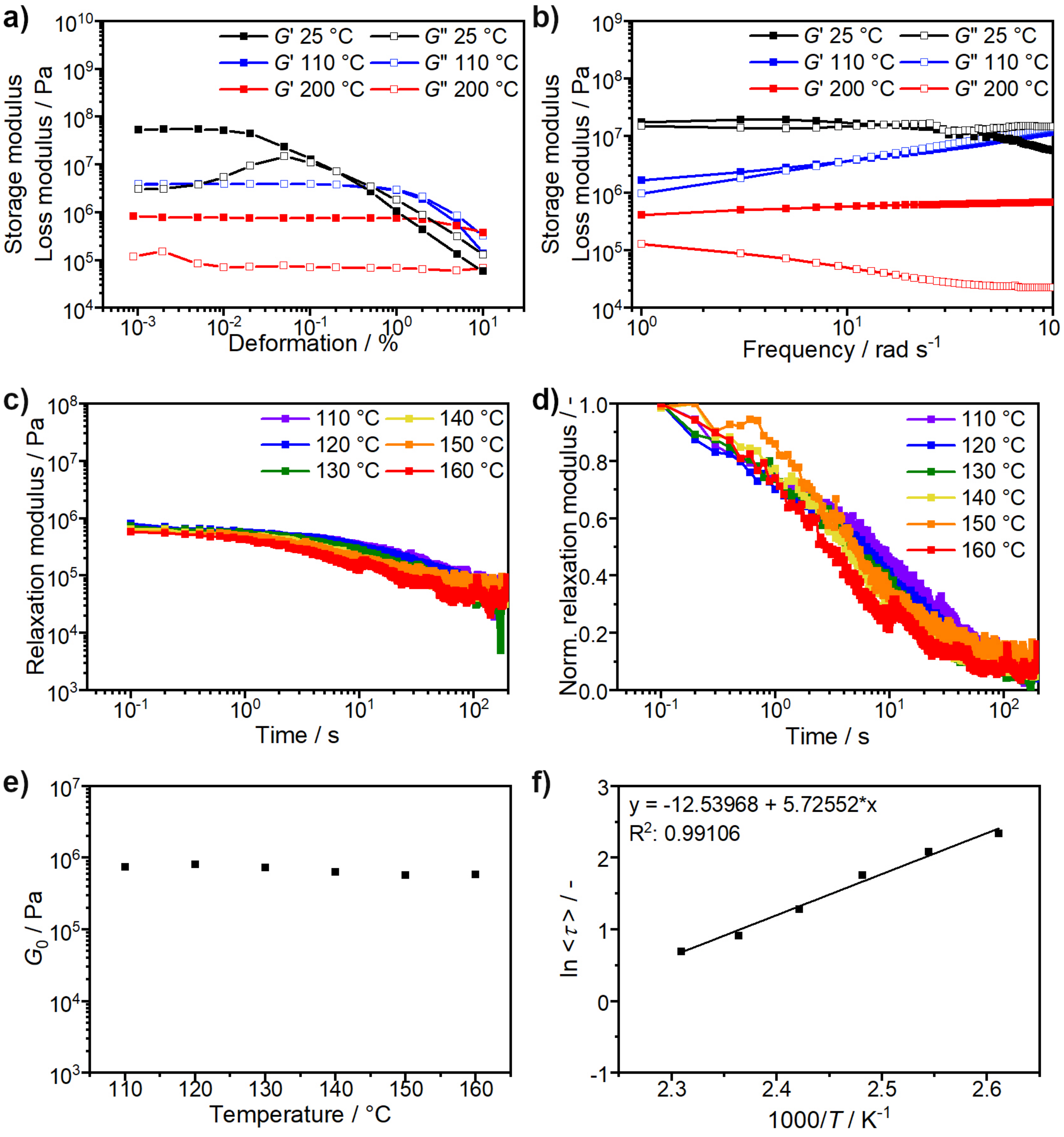

Figure S25: DMA curves of Au0@vMH showing storage moduli G' (filled symbol) and loss moduli G'' (open symbol) in **(a)** amplitude sweep (ω = 10 rad s^{-1}) and **(b)** frequency sweep (γ = 0.01%) at 25 (black square symbol), 110 (blue square symbol) and 180 °C (red square symbol). Stress relaxation DMA performed at various temperatures from 110 to 150 °C with 0.1 % deformation γ, providing **(c)** non-normalized curves, **(d)** normalized curves, **(e)** initial relaxation moduli, and **(f)** average stress relaxation obtained from a stretched exponential decay as a function of temperature.

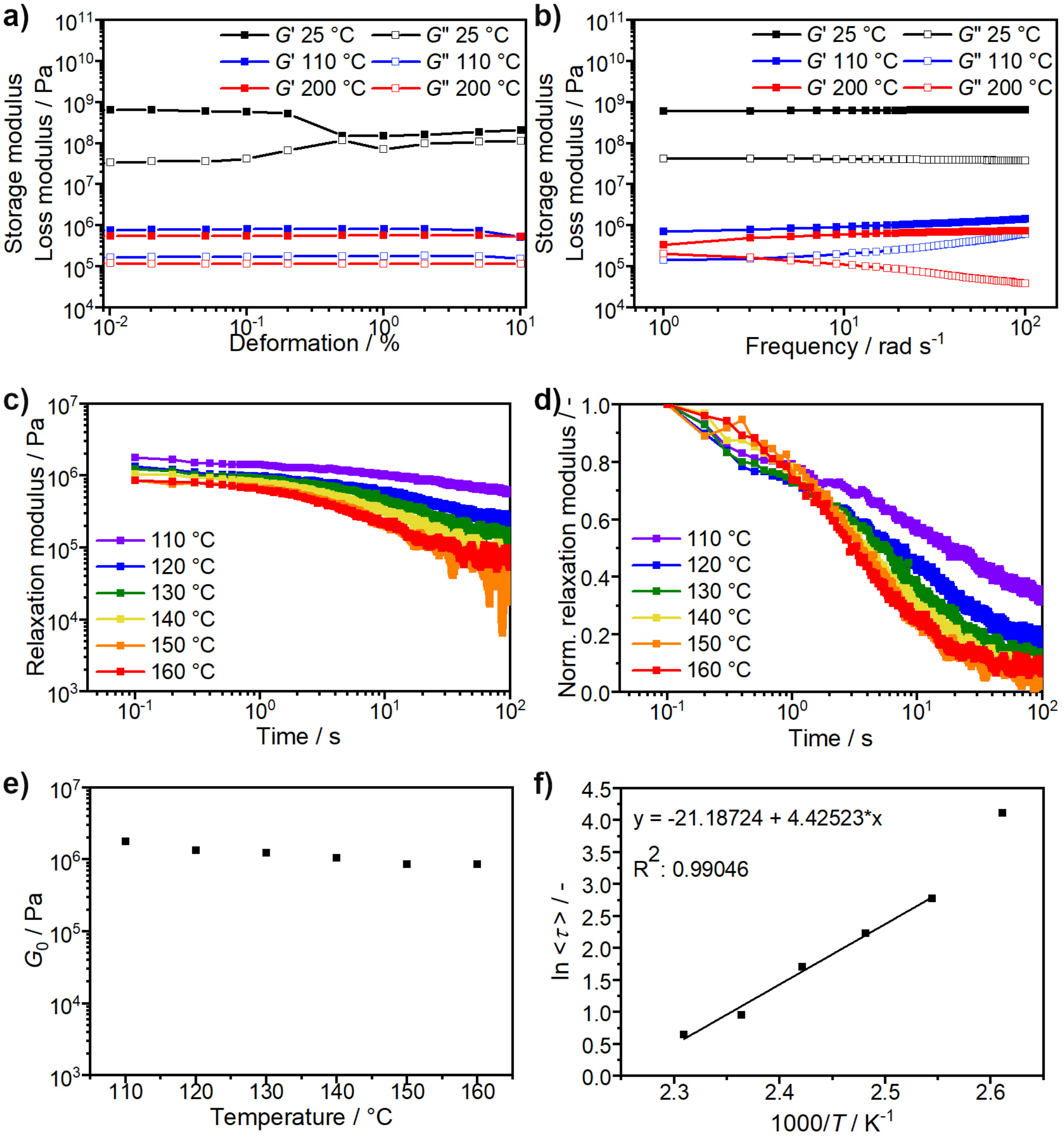

Figure S26: DMA curves of Au0.1@vMH showing storage moduli G' (filled symbol) and loss moduli G'' (open symbol) in **(a)** amplitude sweep (ω = 10 rad s^{-1}) and **(b)** frequency sweep (γ = 0.01%) at 25 (black square symbol), 110 (blue square symbol) and 180 °C (red square symbol). Stress relaxation DMA performed at various temperatures from 110 to 150 °C with 0.1 % deformation γ, providing **(c)** non-normalized curves, **(d)** normalized curves, **(e)** initial relaxation moduli, and **(f)** average stress relaxation obtained from a stretched exponential decay as a function of temperature.

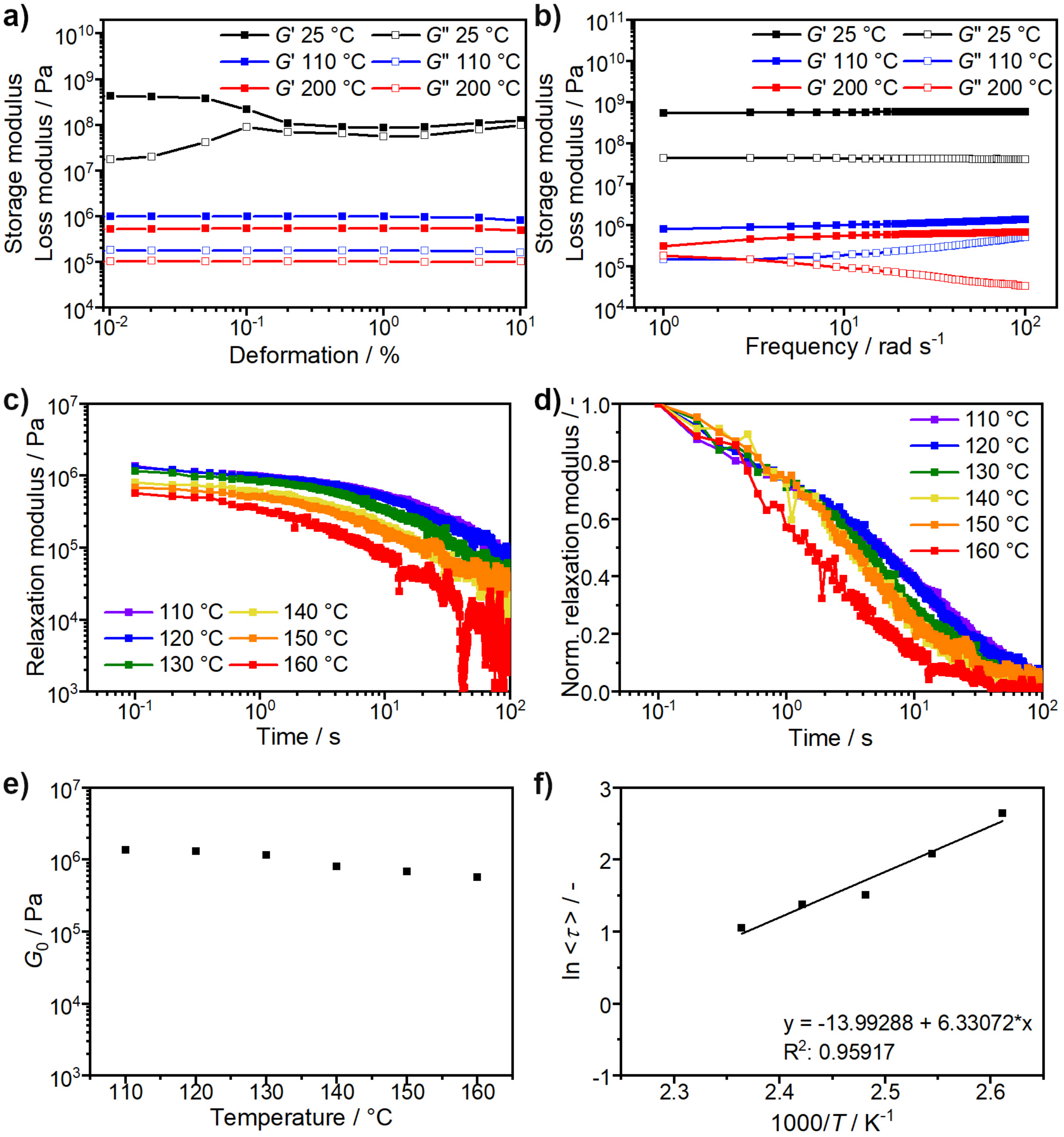

Figure S27: DMA curves of Au0.25@vMH showing storage moduli G' (filled symbol) and loss moduli G'' (open symbol) in **(a)** amplitude sweep (ω = 10 rad s^{-1}) and **(b)** frequency sweep (γ = 0.01%) at 25 (black square symbol), 110 (blue square symbol) and 180 °C (red square symbol). Stress relaxation DMA performed at various temperatures from 110 to 150 °C with 0.1 % deformation γ, providing **(c)** non-normalized curves, **(d)** normalized curves, **(e)** initial relaxation moduli, and **(f)** average stress relaxation obtained from a stretched exponential decay as a function of temperature.

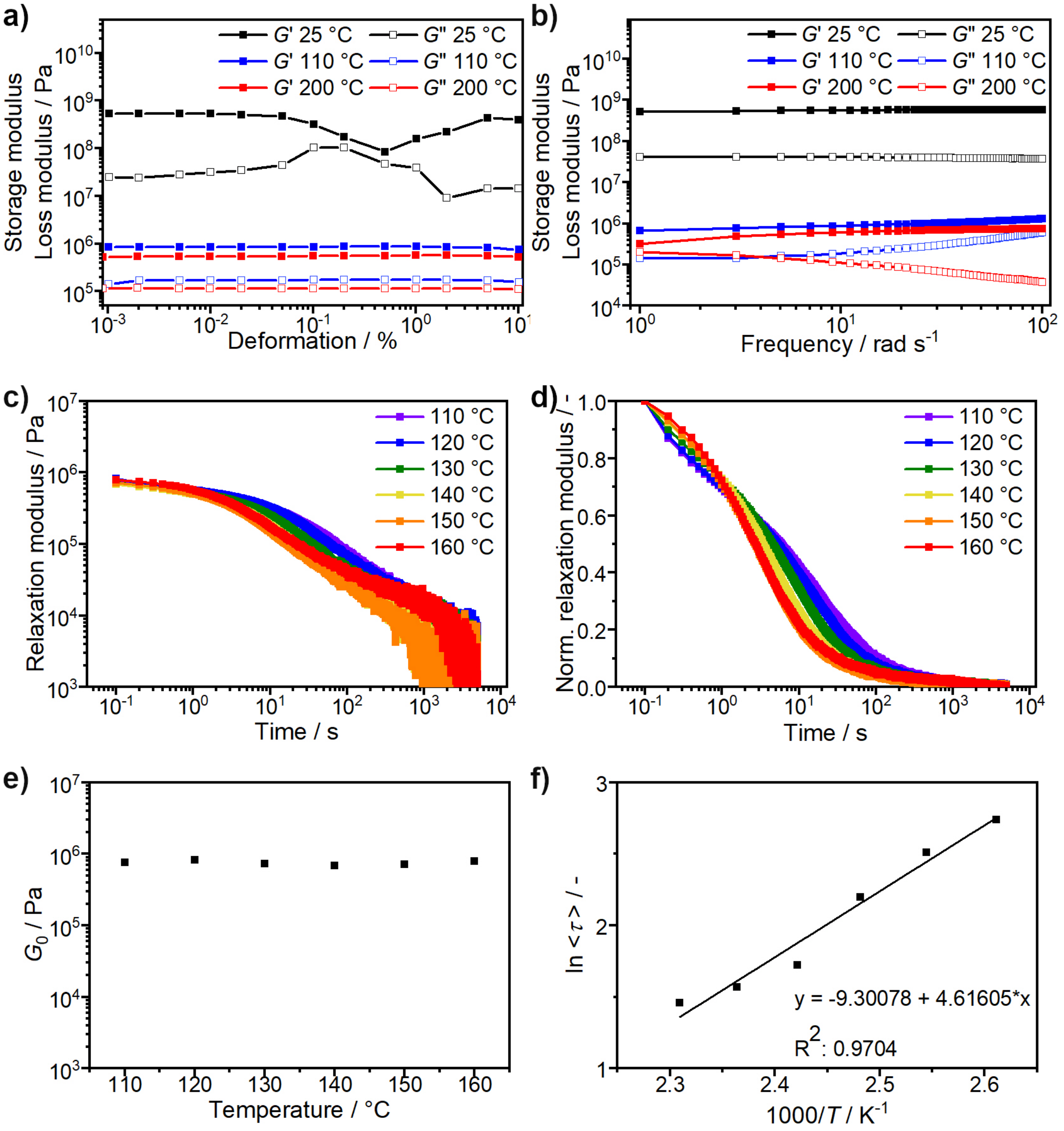

Figure S28: DMA curves of Au0.5@vMH showing storage moduli G' (filled symbol) and loss moduli G'' (open symbol) in **(a)** amplitude sweep (ω = 10 rad s^{-1}) and **(b)** frequency sweep (γ = 0.01%) at 25 (black square symbol), 110 (blue square symbol) and 180 °C (red square symbol). Stress relaxation DMA performed at various temperatures from 110 to 150 °C with 0.1 % deformation γ, providing **(c)** non-normalized curves, **(d)** normalized curves, **(e)** initial relaxation moduli, and **(f)** average stress relaxation obtained from a stretched exponential decay as a function of temperature.

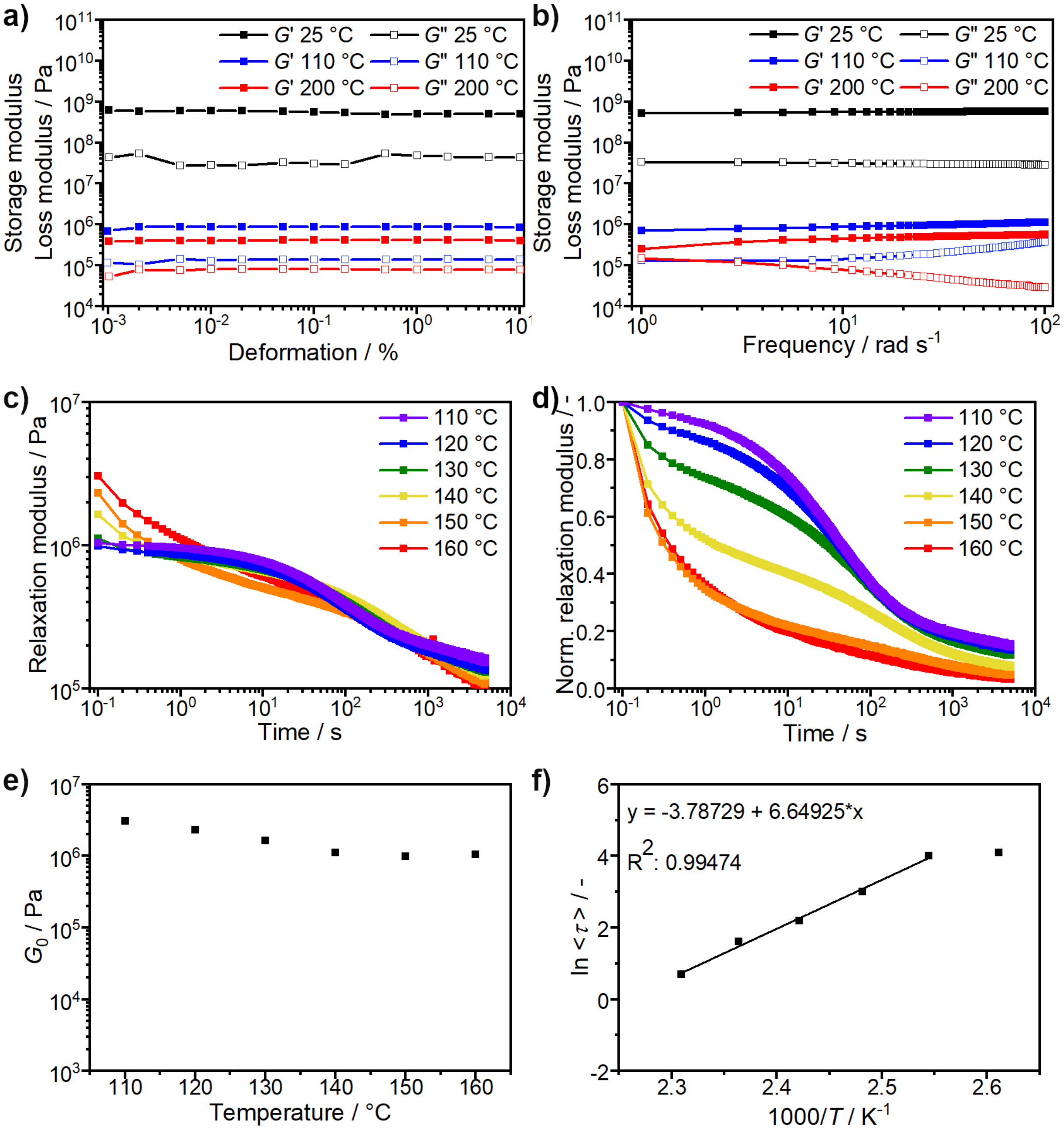

Figure S29: DMA curves of Au1.0@vMH showing storage moduli *G′* (filled symbol) and loss moduli *G″* (open symbol) in **(a)** amplitude sweep (ω = 10 rad s^{-1}) and **(b)** frequency sweep (γ = 0.01%) at 25 (black square symbol), 110 (blue square symbol) and 180 °C (red square symbol). Stress relaxation DMA performed at various temperatures from 110 to 150 °C with 0.1 % deformation γ, providing **(c)** non-normalized curves, **(d)** normalized curves, **(e)** initial relaxation moduli, and **(f)** average stress relaxation obtained from a stretched exponential decay as a function of temperature.

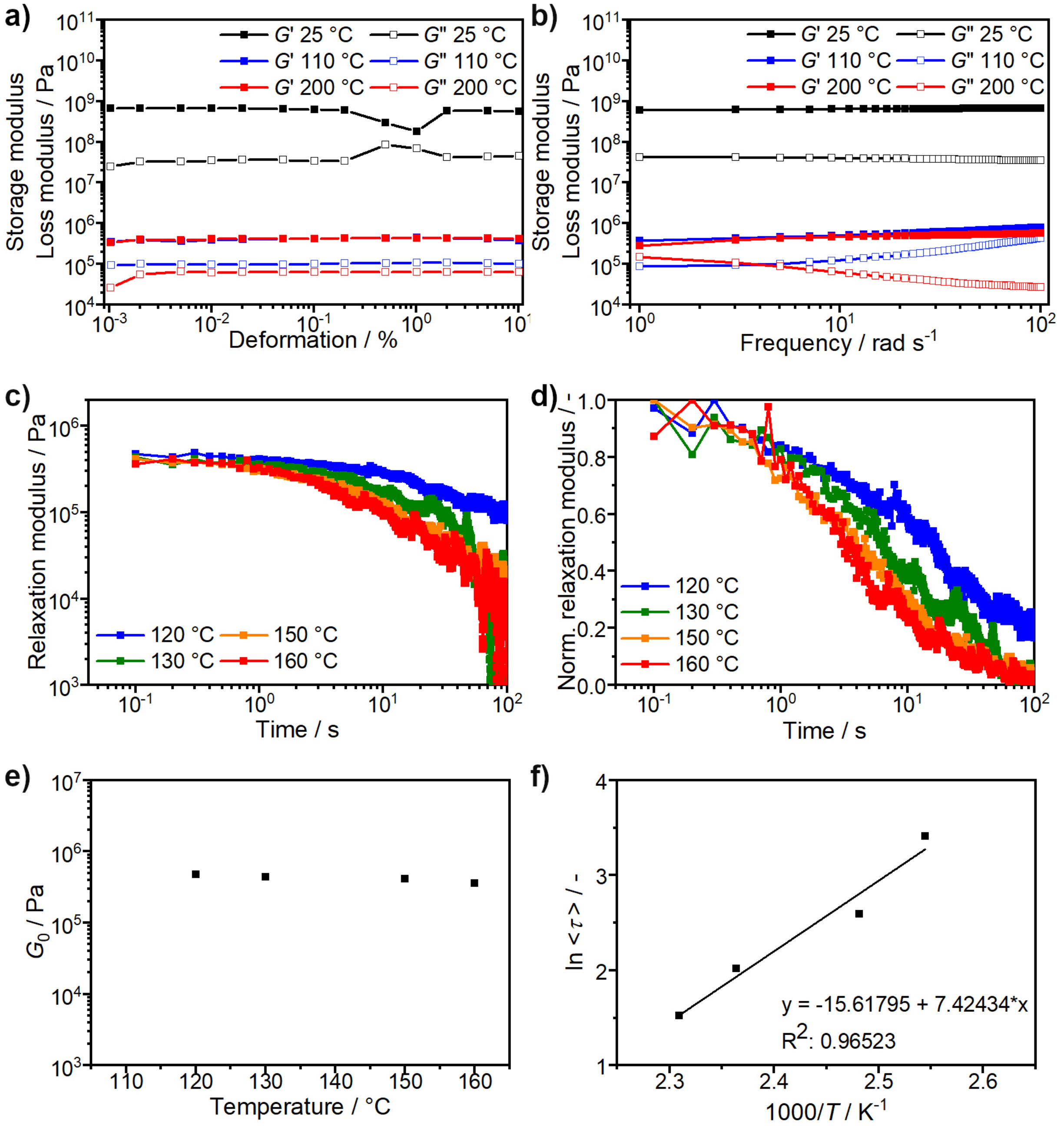

Figure S30: DMA curves of Au1.5@vMH showing storage moduli G' (filled symbol) and loss moduli G'' (open symbol) in **(a)** amplitude sweep (ω = 10 rad s^{-1}) and **(b)** frequency sweep (γ = 0.01%) at 25 (black square symbol), 110 (blue square symbol) and 180 °C (red square symbol). Stress relaxation DMA performed at various temperatures from 110 to 150 °C with 0.1 % deformation γ, providing **(c)** non-normalized curves, **(d)** normalized curves, **(e)** initial relaxation moduli, and **(f)** average stress relaxation obtained from a stretched exponential decay as a function of temperature.

Table S4: The parameter of relaxation distribution β.

Samples	T = 110 °C	T = 120 °C	T = 130 °C	T = 140 °C	T = 150 °C	T = 160 °C
Au0@vMH	0.10827	0.0912	0.06219	0.05109	0.05668	0.01066
Au0.1@vMH	-	0.02756	0.05553	0.11359	0.39276	0.47630
Au0.25@vMH	0.57191	0.44114	0.43714	0.48748	0.28798	-
Au0.5@vMH	0.24911	0.17149	0.13265	0.20396	0.18613	0.09328
Au1.0@vMH	0.06575	0.08042	0.11449	0.07082	0.05894	0.05538
Au1.5@vMH	-	0.07445	0.2219	-	0.35525	0.51587

Table S5: Average stress relaxation time $< \tau >$ obtained from a stretched exponential decay.

Samples	T = 110 °C	T = 120 °C	T = 130 °C	T = 140 °C	T = 150 °C	T = 160 °C
Au0@vMH	10.4	8.0	5.8	3.6	2.5	2.0
Au0.1@vMH	-	16.0	9.3	5.5	2.6	1.9
Au0.25@vMH	14.1	8.0	4.5	3.9	2.9	-
Au0.5@vMH	15.5	12.3	9.0	5.6	4.8	4.3
Au1.0@vMH	-	-	20.0	9.0	5.0	2.0
Au1.5@vMH	-	30.2	13.4	-	7.5	4.5

S12 Reprocessing, Reshaping, Self-healing, and Shape-memory

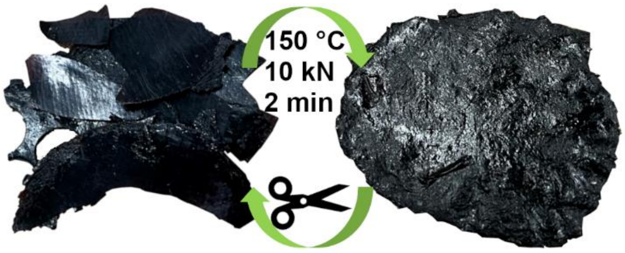

Figure S31: Vitrimer reprocessing cycles by grinding into small pieces and hydraulic pressing (150 °C, 10 kN, 2 min). The sample is Au0.5@vMH and the process shown in **Video S1**.

Table S6: Mechanical properties of pristine and recycled Au0.25@vMH measured by UTM.

	$E_{t\emptyset}$ [a] [MPa]	$\sigma_{m\emptyset}$ [a] [MPa]	$\varepsilon_{m\emptyset}$ [a] [%]
Pristine	406.3±6.9^{A}	30.8±1.1^{A}	26.9±1.4^{A}
Recycle 1	410.6±1.6^{A}	28.6±1.6^{A}	26.3±1.4^{A}
Recycle 2	403.8±4.6^{A}	30.6±1.0^{A}	27.4±0.4^{A}
Recycle 3	403.0±3.3^{A}	27.1±2.0^{A}	25.9±1.1^{A}

[a] Stress at break ($\sigma_{m\emptyset}$), strain at break ($\varepsilon_{m\emptyset}$), and E-modulus ($E_{t\emptyset}$) determined by stress-strain measurements at room temperature with a strain rate of 10 mm/min. Mean values and standard deviation (n=3) in the same column with different superscript letters (capital letters) indicate significant differences using Tukey's range test accepted 0.05 probability as significant.

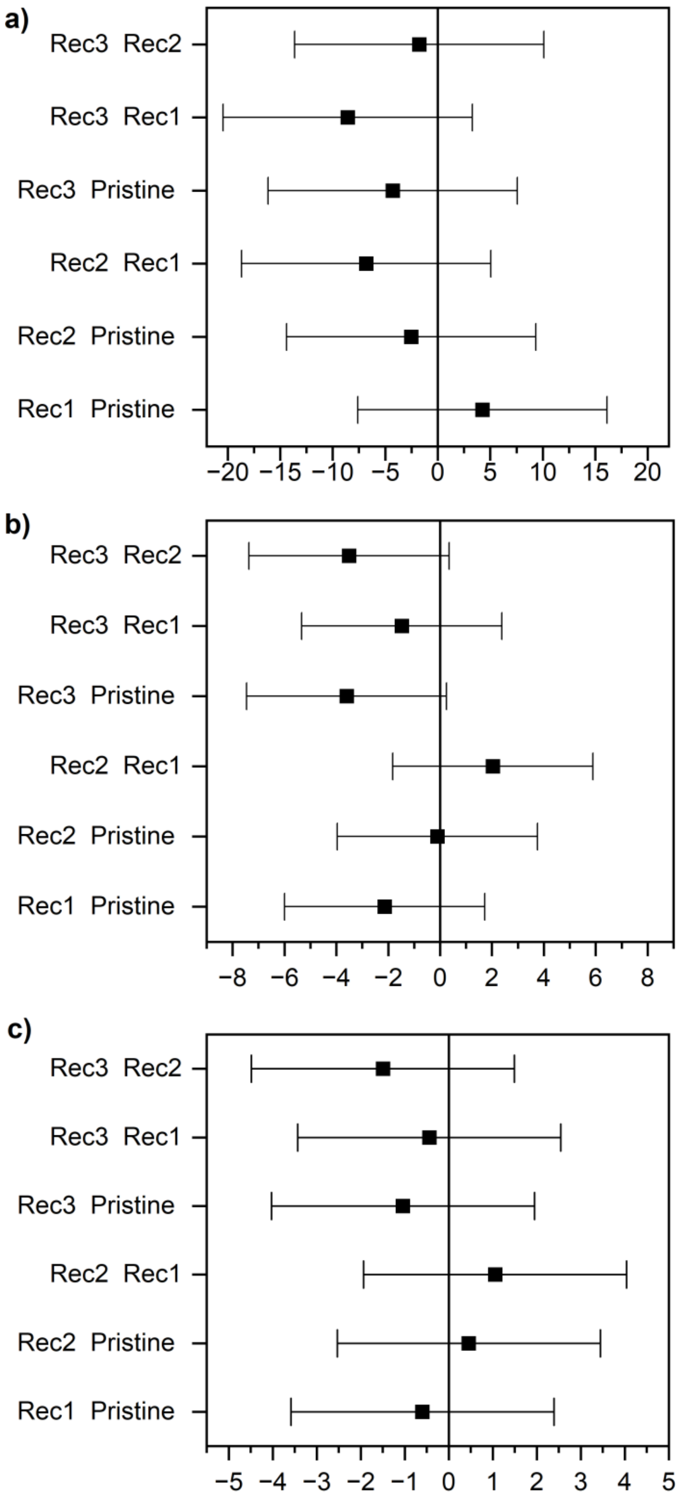

Figure S32: Means comparison plot of **(a)** *E*-modulus, **(b)** stress at break, **(c)** strain at break of pristine and recycle (3 times) of Au0.25@vMH (n=3) from Tukey's range test in one way ANOVA accepted 0.05 probability as significant.

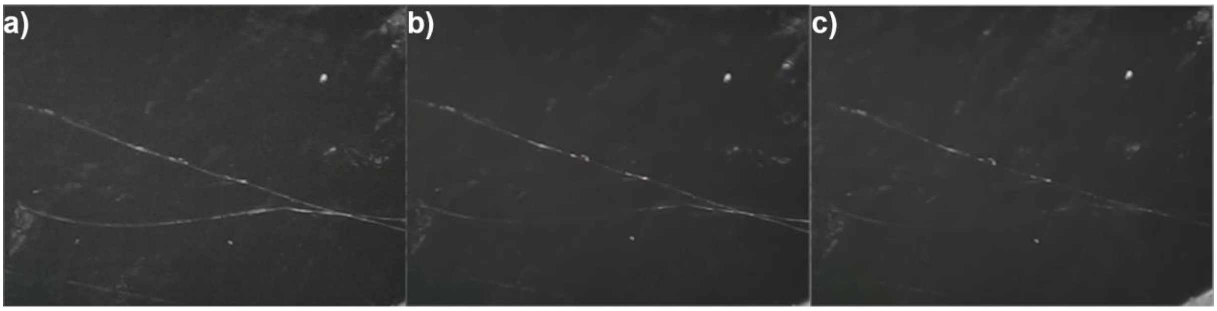

Figure S33: Self-healing of vitrimer (Au0.5@vMHN) over time **(a)** when scratched (0 s) and after heating with a heat gun at 130 °C for **(b)** 30 s and **(c)** 60 s. The pictures were taken from **Video S2**.

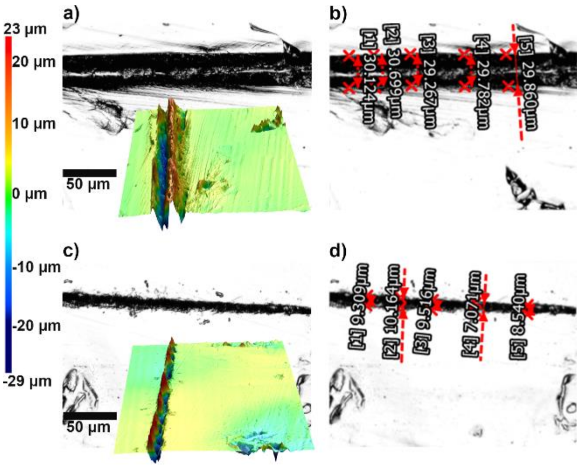

Figure S34: **(a)** Confocal microscope image and surface profile of vitrimer (Au0@vMH), and **(b)** width of cut surface when scratched (0 min). **(c)** Image and surface profile, and **(d)** width of the cut surface after heating with a heat gun at 140 °C for 1 min. The scratch was made with a cutter. The scale bar is 50 µm.

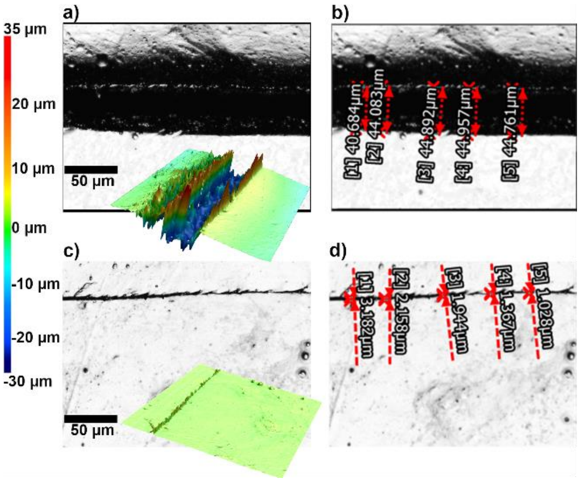

Figure S35: (a) Confocal microscope image and surface profile of vitrimer (Au0.1@vMH), and **(b)** width of cut surface when scratched (0 min). **(c)** Image and surface profile, and **(d)** width of the cut surface after heating with a heat gun at 140 °C for 1 min. The scratch was made with a cutter. The scale bar is 50 μm.

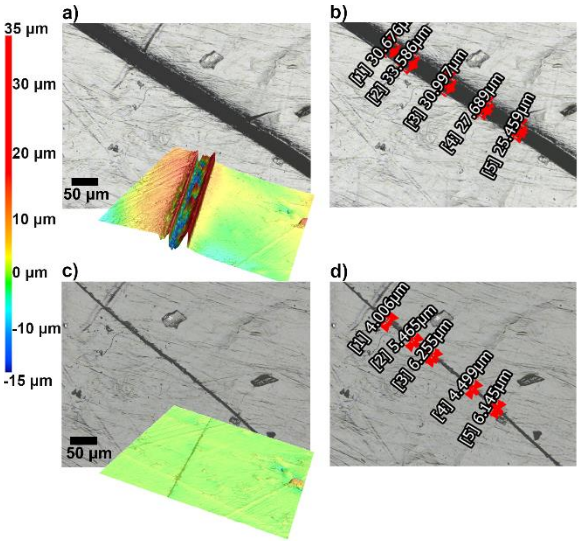

Figure S36: (a) Confocal microscope image and surface profile of vitrimer (Au0.5@vMH), and **(b)** width of cut surface when scratched (0 min). **(c)** Image and surface profile, and **(d)** width of the cut surface after heating with a heat gun at 140 °C for 1 min. The scratch was made with a cutter. The scale bar is 50 μm.

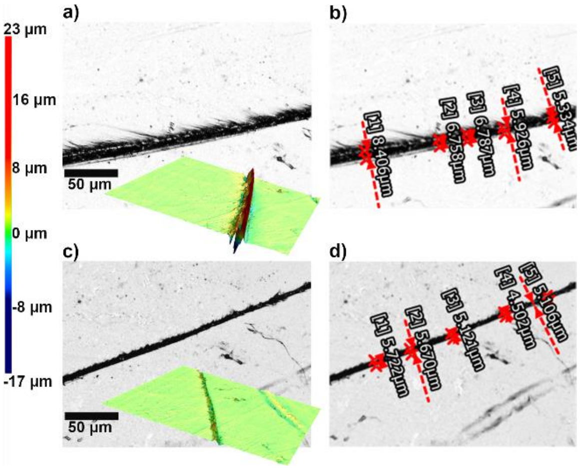

Figure S37: **(a)** Confocal microscope image and surface profile of vitrimer (Au1.0@vMH), and **(b)** width of cut surface when scratched (0 min). **(c)** Image and surface profile, and **(d)** width of the cut surface after heating with a heat gun at 140 °C for 1 min. The scratch was made with a cutter. The scale bar is 50 μm.

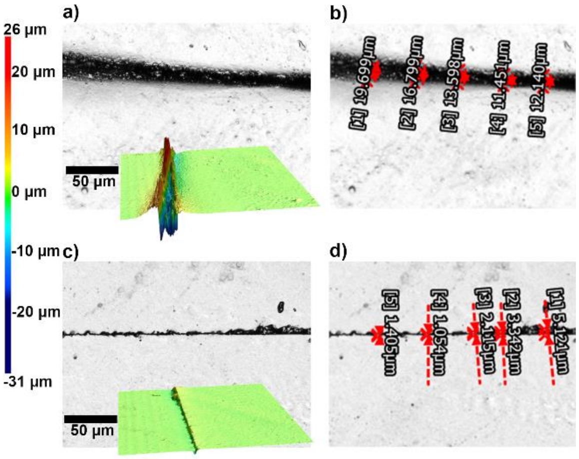

Figure S38: **(a)** Confocal microscope image and surface profile of vitrimer (Au1.5@vMH), and **(b)** width of cut surface when scratched (0 min). **(c)** Image and surface profile, and **(d)** width of the cut surface after heating with a heat gun at 140 °C for 1 min. The scratch was made with a cutter. The scale bar is 50 μm.

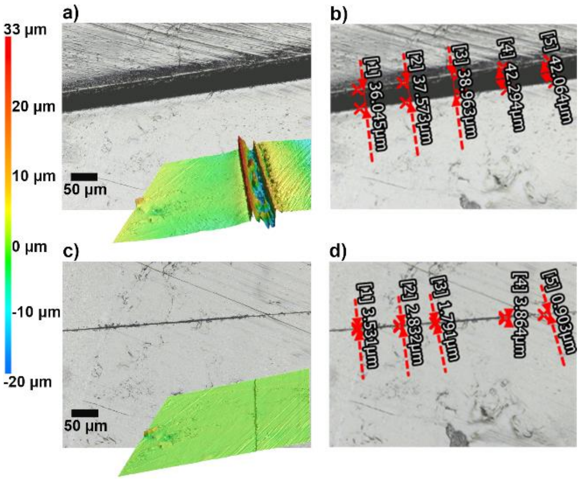

Figure S39: (a) Confocal microscope image and surface profile of vitrimer (Au0.5@vMHM), and **(b)** width of cut surface when scratched (0 min). **(c)** Image and surface profile, and **(d)** width of the cut surface after heating with a heat gun at 140 °C for 1 min. The scratch was made with a cutter. The scale bar is 50 μm.

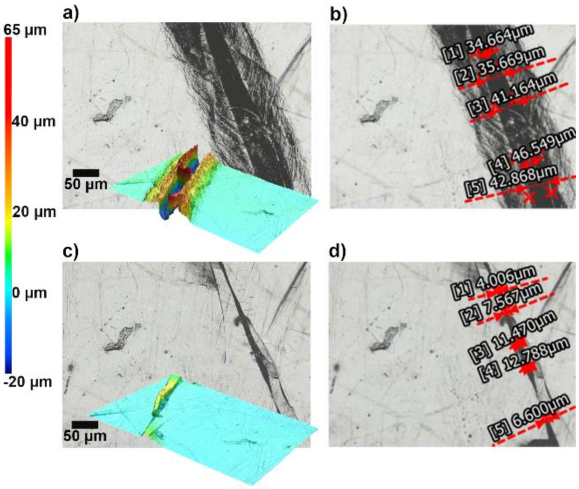

Figure S40: (a) Confocal microscope image and surface profile of vitrimer (Au0.5@vMHD), and **(b)** width of cut surface when scratched (0 min). **(c)** Image and surface profile, and **(d)** width of the cut surface after heating with a heat gun at 140 °C for 1 min. The scratch was made with a cutter. The scale bar is 50 μm.

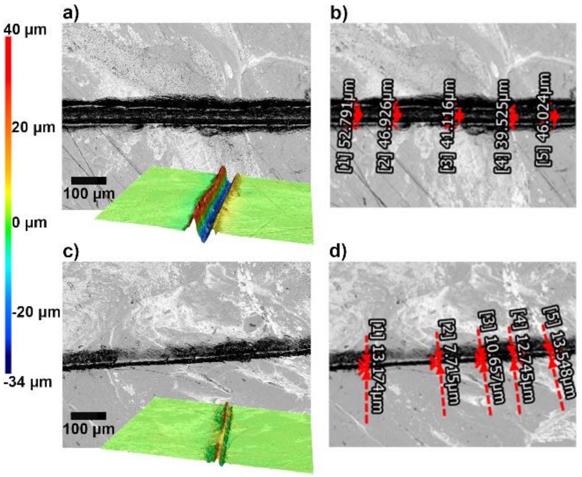

Figure S41: (a) Confocal microscope image and surface profile of vitrimer (Au0.5@vMHN), and **(b)** width of cut surface when scratched (0 min). **(c)** Image and surface profile, and **(d)** width of the cut surface after heating with a heat gun at 140 °C for 1 min. The scratch was made with a cutter. The scale bar is 50 µm.

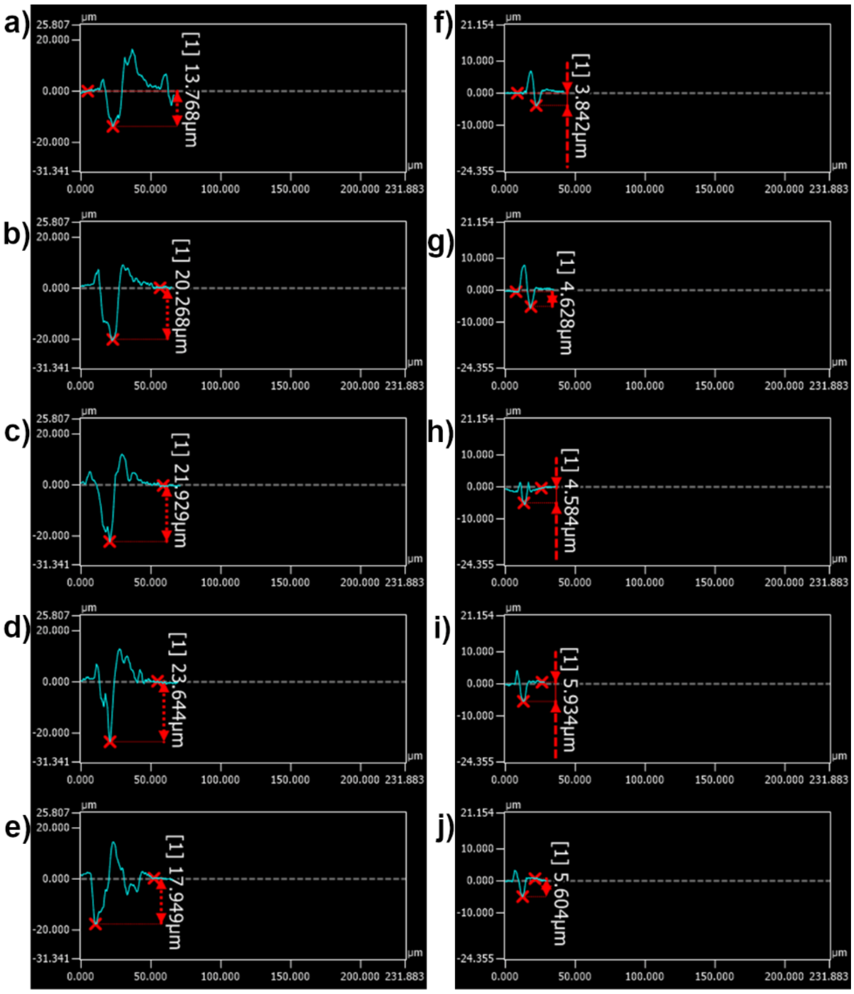

Figure S42: Surface profiles of **(a–e)** damaged by cutter and **(f–j)** healed materials (Au0@vMH) after applying heat by a heat gun at 140 °C for 1 min.

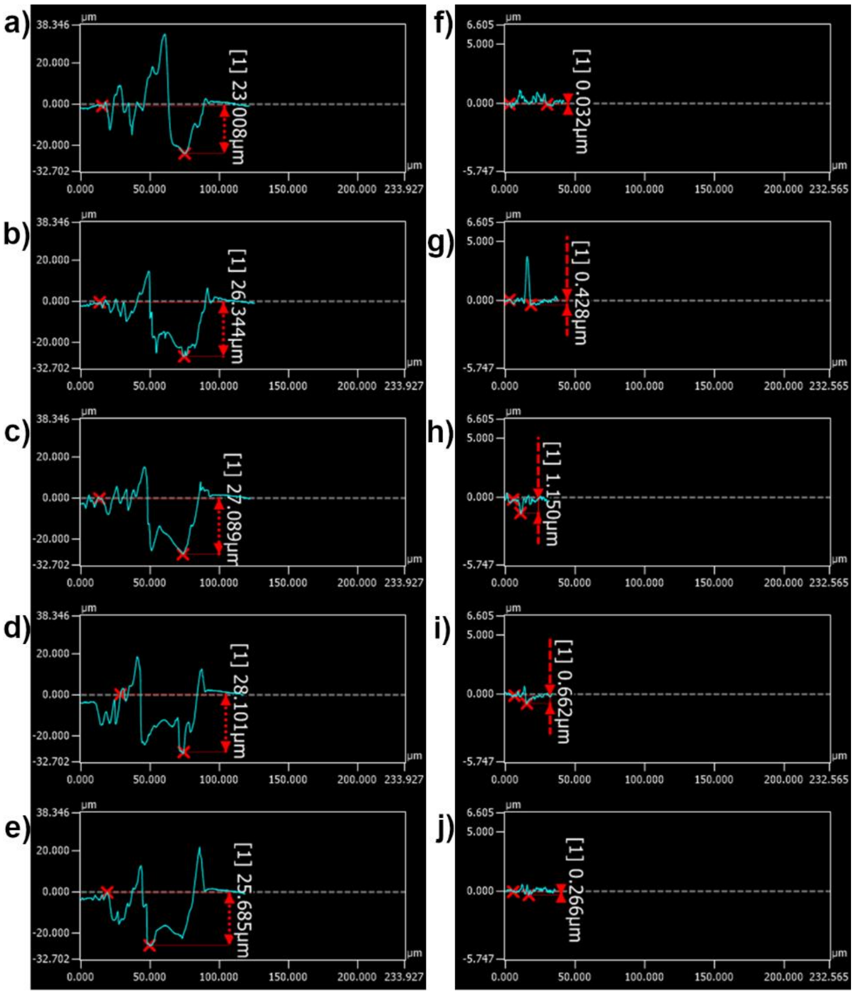

Figure S43: Surface profiles of **(a–e)** damaged by cutter and **(f–j)** healed materials (Au0.1@vMH) after applying heat by a heat gun at 140 °C for 1 min.

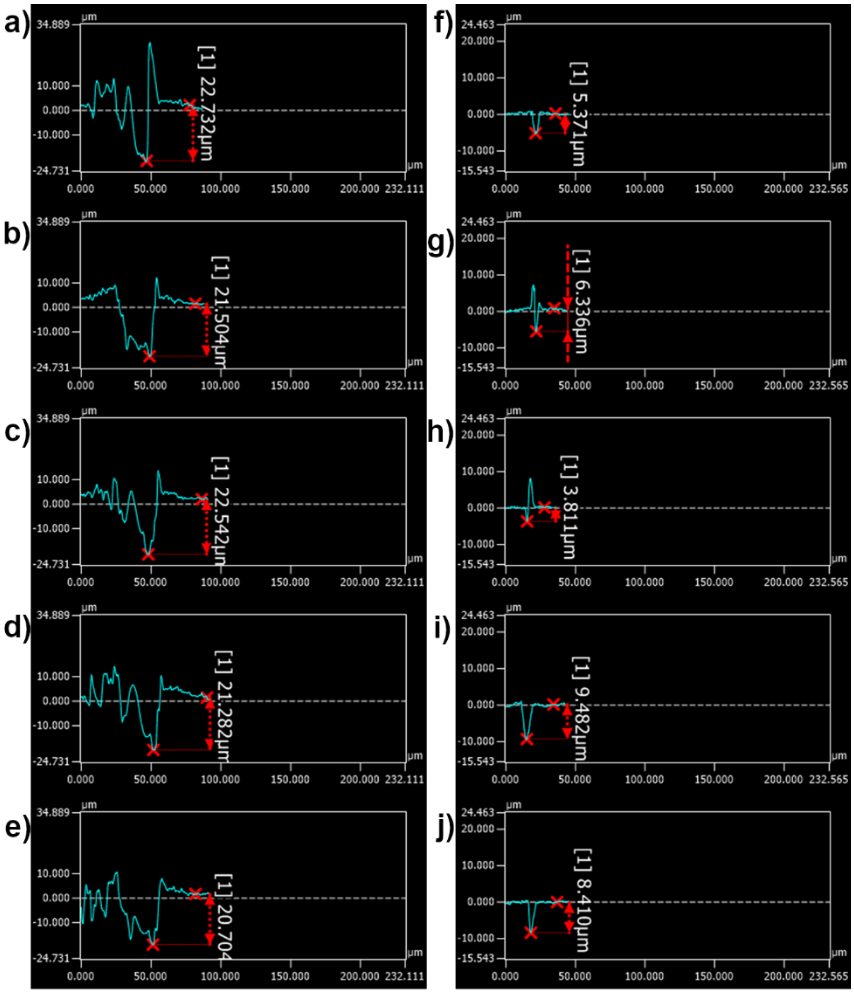

Figure S44: Surface profiles of **(a–e)** damaged by cutter and **(f–j)** healed materials (Au0.25@vMH) after applying heat by a heat gun at 140 °C for 1 min.

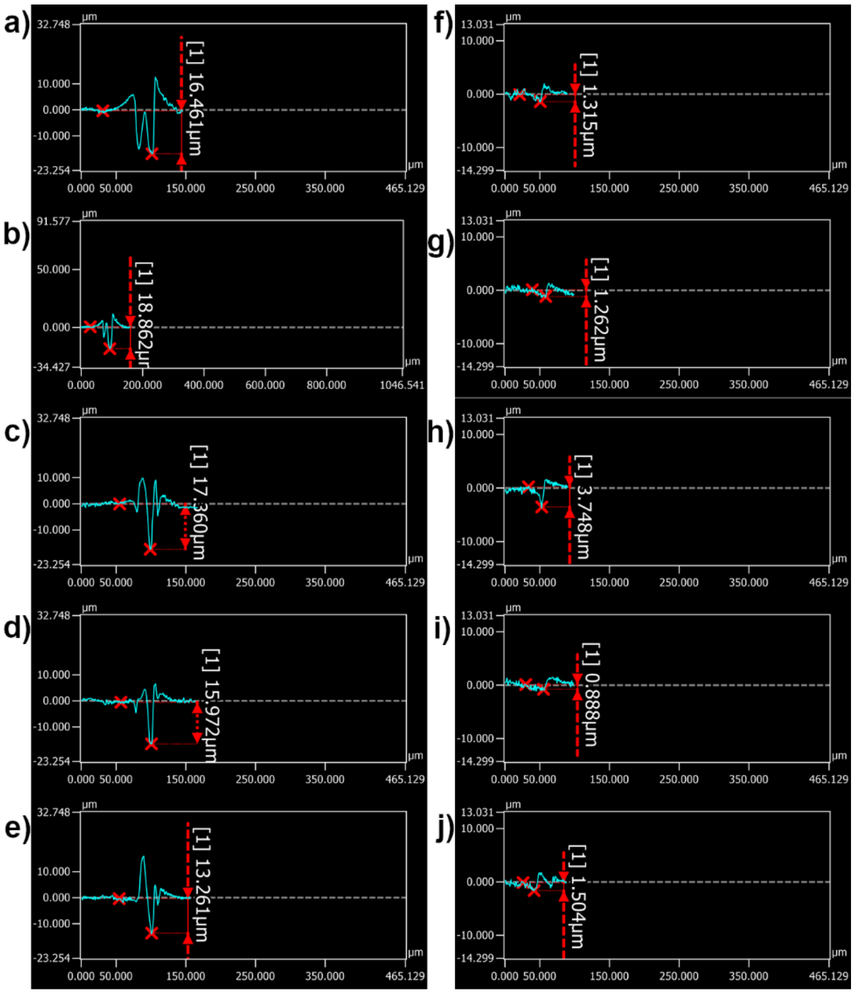

Figure S45: Surface profiles of **(a–e)** damaged by cutter and **(f–j)** healed materials (Au0.5@vMH) after applying heat by a heat gun at 140 °C for 1 min.

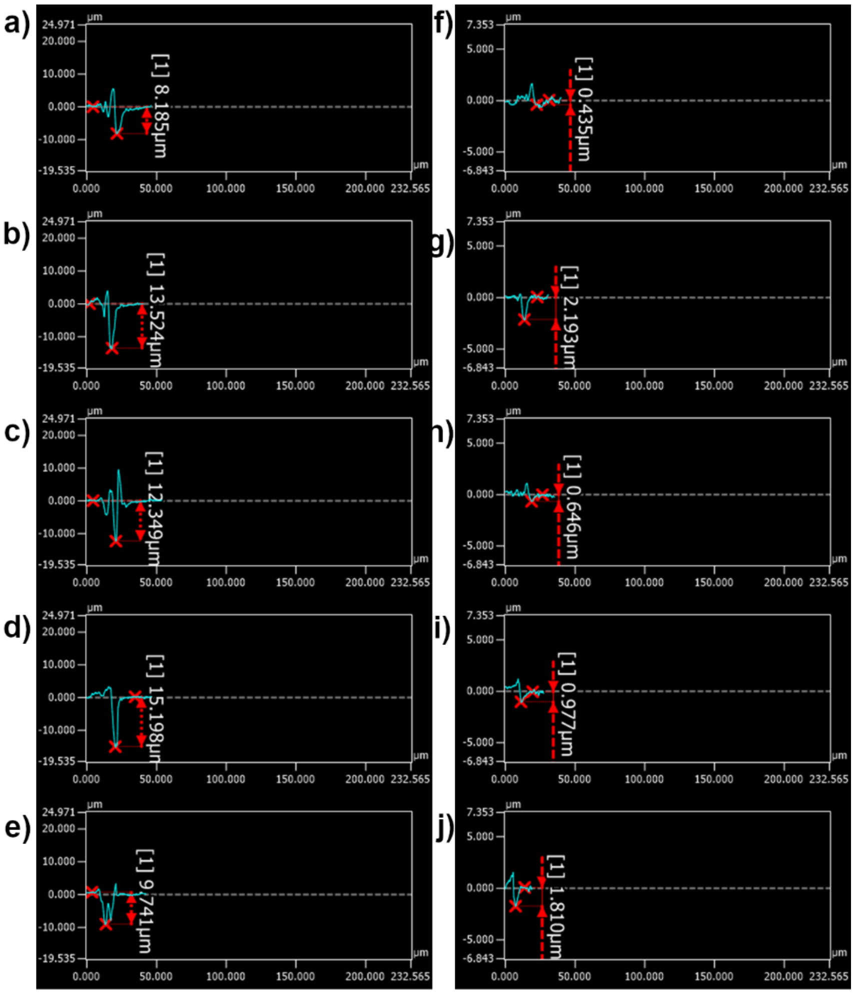

Figure S46: Surface profiles of **(a–e)** damaged by cutter and **(f–j)** healed materials (Au1.0@vMH) after applying heat by a heat gun at 140 °C for 1 min.

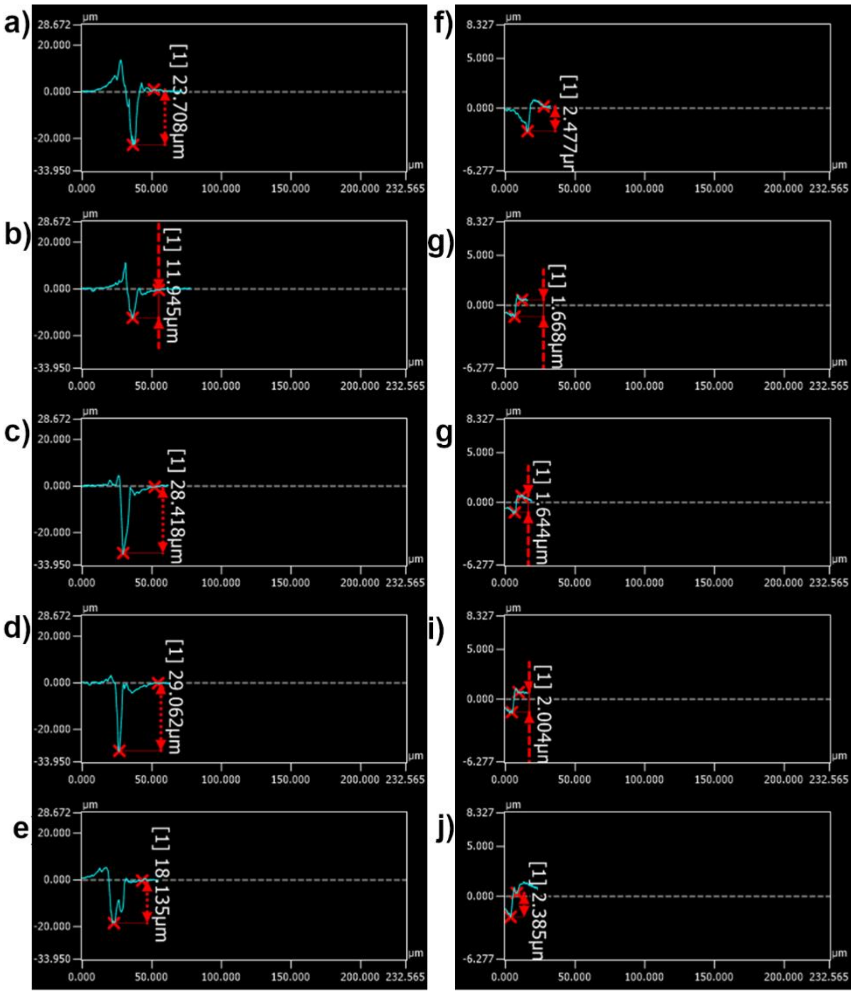

Figure S47: Surface profiles of **(a–e)** damaged by cutter and **(f–j)** healed materials (Au1.5@vMH) after applying heat by a heat gun at 140 °C for 1 min.

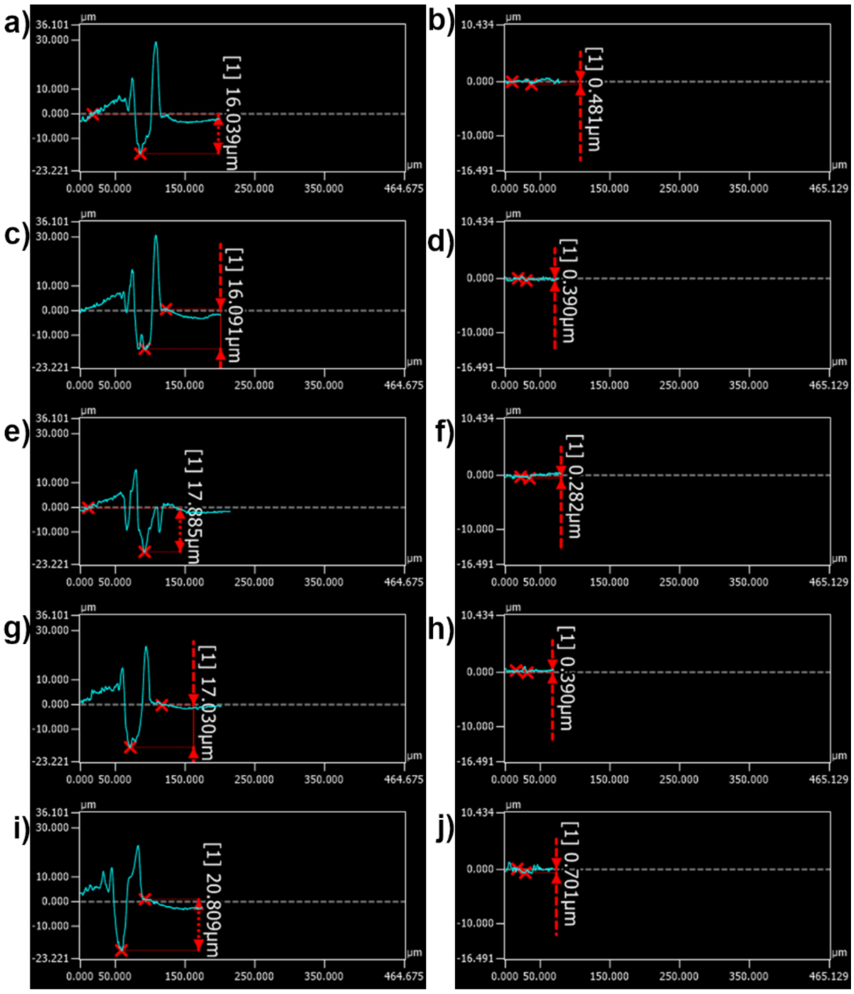

Figure S48: Surface profiles of **(a–e)** damaged by cutter and **(f–j)** healed materials (Au0.5@vMHM) after applying heat by a heat gun at 140 °C for 1 min.

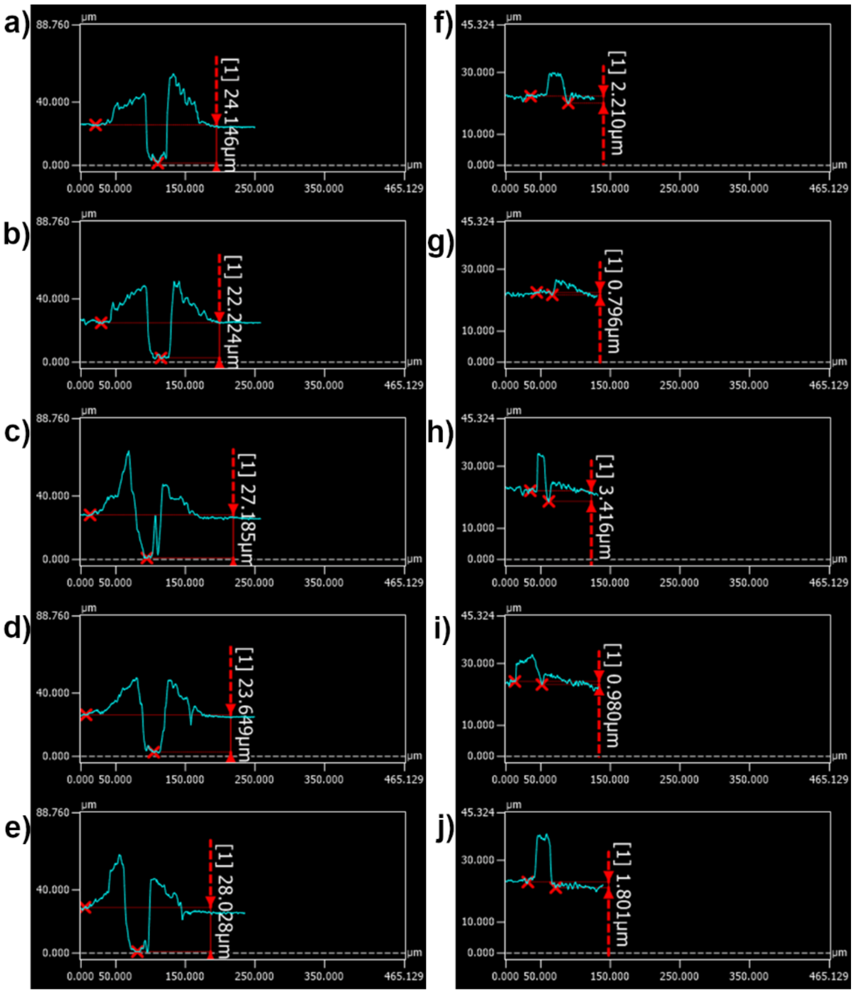

Figure S49: Surface profiles of **(a-e)** damaged by cutter and **(f-j)** healed materials (Au0.5@vMHD) after applying heat by a heat gun at 140 °C for 1 min.

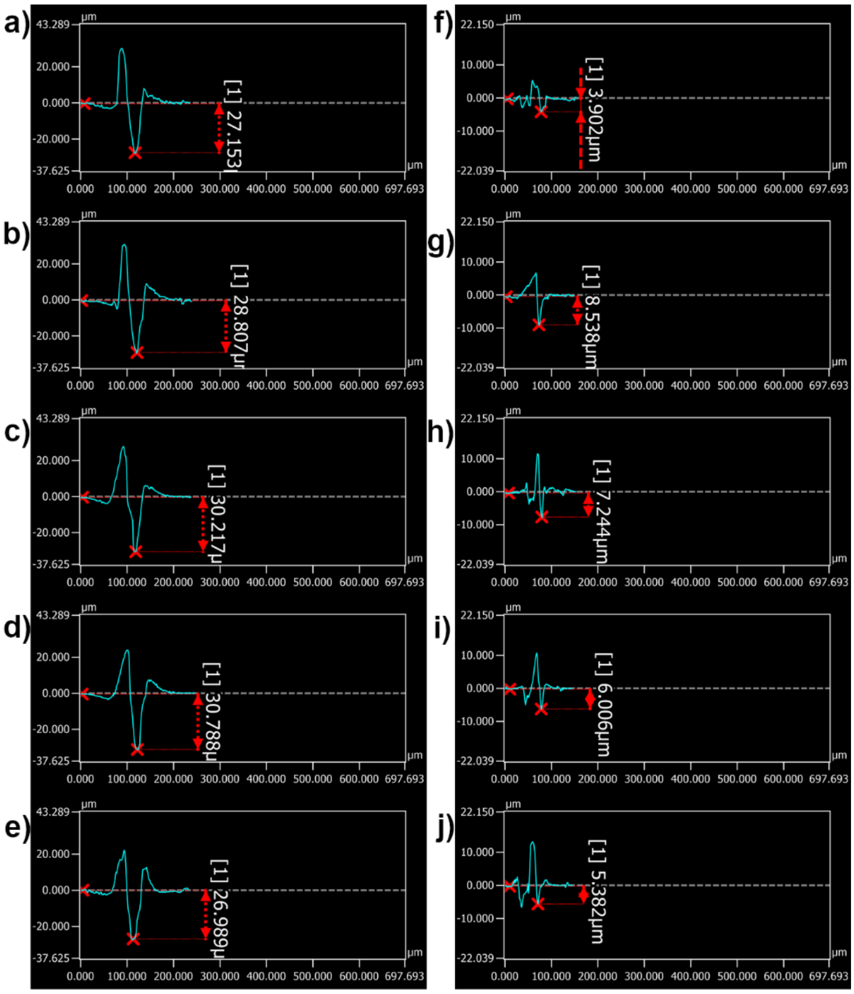

Figure S50: Surface profiles of **(a-e)** damaged by cutter and **(f-j)** healed materials (Au0.5@vMHN) after applying heat by a heat gun at 140 °C for 1 min.

Table S7: Average of deepness measured by a confocal microscope.

Samples	Depth [µm] [a]		Width [µm] [a]	
	0 min (scratch)	1 min (healed)	0 min (scratch)	1 min (healed)
Au0@vMH	19.51±3.43	4.92±0.76	29.95±0.47	8.92±1.06
Au0.1@vMH	26.05±1.72	0.50±0.39	43.88±1.63	1.94±0.74
Au0.25@vMH	21.75±0.77	6.68±2.04	31.13±0.60	5.41±0.65
Au0.5@vMH	16.38±1.85	1.74±1.02	29.68±2.82	5.27±0.89
Au1.0@vMH	11.80±2.53	1.21±0.68	6.64±1.04	5.22±0.45
Au1.5@vMH	22.37±6.30	2.04±0.35	22.37±6.30	2.61±1.48
Au0.5@vMHM	25.05±2.20	1.84±0.94	25.05±2.20	8.49±3.22
Au0.5@vMHD	29.63±3.60	4.90±2.46	29.63±3.60	11.57±2.17
Au0.5@vMHN	17.57±1.76	0.45±0.14	17.57±1.76	2.58±1.10

[a] Mean values and standard deviation calculated from surface profiles (n=5).

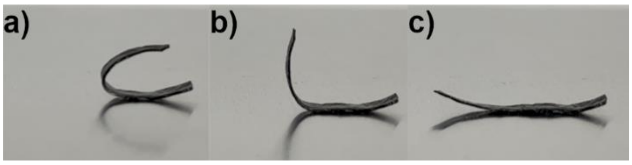

Figure S51: Shape-memory behavior initiated by a heat gun at 110 °C over time; **(a)** temporary shape (0 s) and after heating with a heat gun at 130 °C for **(b)** 30 s and **(c)** 60 s. The sample is Au0.5@vMHN (0.5 mm thickness). The pictures were taken from **Video S12.3**.

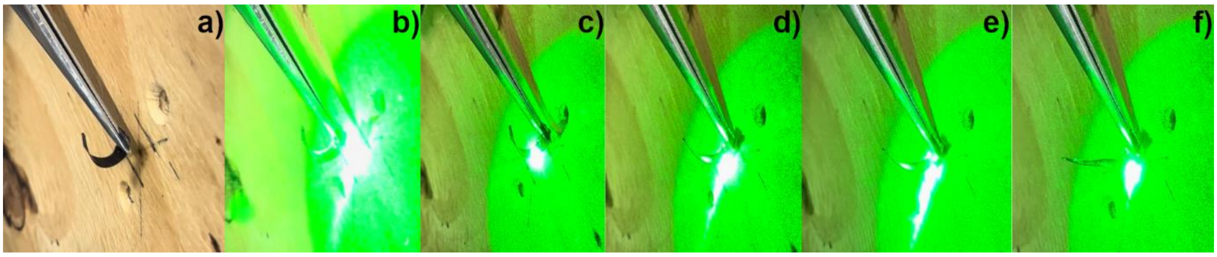

Figure S52: Shape-memory behavior initiated by photothermal effect (λ = 532 nm, 200 mW) over time; temporary shape **(a)** before and during illumination for **(b)** 0 s, **(c)** 10 s, **(d)** 20 s, **(e)** 30 s, and **(f)** 40 s. The sample is Au1.0@vMH (0.5 mm thickness). The pictures were taken from **Video S4**.

Table S8: Average of the wavelength at the peaks of UV–Vis spectra of pristine and recycled vitrimer films.

	λ_{max} [nm][a]	
	Au1.0@vMH	Au1.5@vMH
Pristine	558.7±1.1[A]	555.9±2.8[A]
Recycle 1	557.9±1.1[A]	558.6±0.9[A]
Recycle 2	555.7±1.6[A]	554.9±1.4[A]
Recycle 3	555.4±3.1[A]	555.2±2.2[A]

[a] Mean values and standard deviation (n=4) in the same column with different superscript letters (capital letter) indicate significant differences using Tukey's range test accepted 0.05 probability as significant.

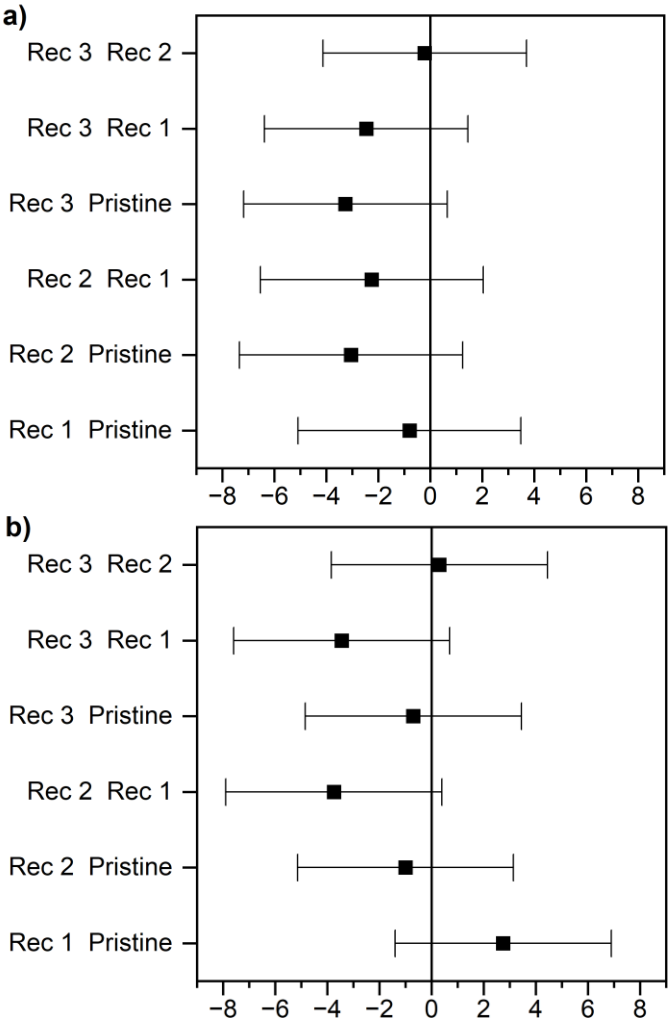

Figure S53: Means comparison plot of the wavelength at the peaks of UV–Vis spectra of pristine and recycle (3 times) **(a)** Au1.0@vMH and **(b)** Au1.5@vMH from Tukey's range test in one way ANOVA accepted 0.05 probability as significant (n=4).

S13 Investigation of Plasmonic Heating of the Nanocomposites

An image of the experimental setup for the plasmonic heating efficiency is shown in **Figure S54**. The irradiation time-dependent temperature curves of Au@vMH films with different doping concentrations and film thicknesses that were not shown in the main text are shown in the following (**Figure S55**and **Figure S56**). The results are also listed in **Table S9**.

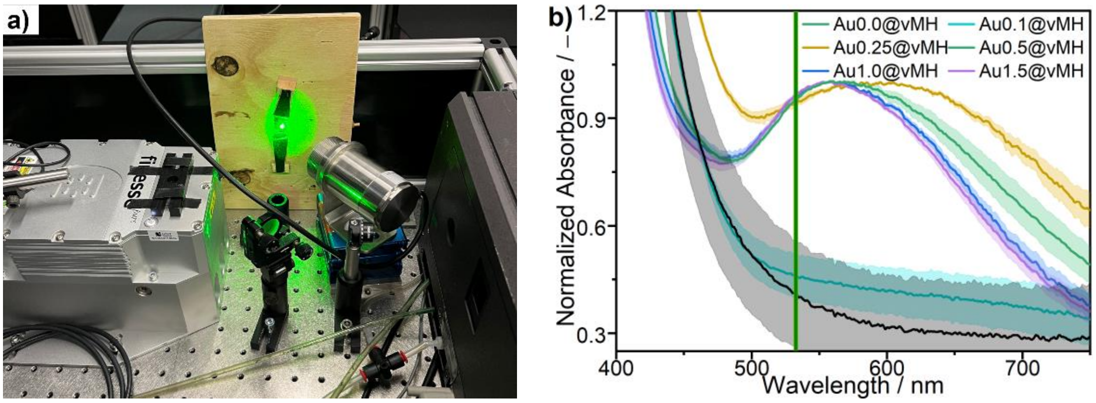

Figure S54: (a) Experimental setup to determine the photothermal heating efficiency with a green laser (λ = 532 nm, P = 50–200 mW, e^{-2} spot size A = 3.2 mm^2 that is directed to the fixed Au@vMH-film. The temperature was monitored with an infrared camera and the laser thermometer that is focused on the spot of the green laser. **(b)** Normalized UV–Vis extinction spectra of the Au@vMH-films with gold contents of 0, 0.1, 0.25, 0.5, 1, and 1.5 wt% with the wavelength of the excitation laser (λ = 532 nm) for plasmonic heating as green region. The spectra of the films were obtained by averaging the spectra of four different spots on the film, and their standard deviation was depicted as an error bar.

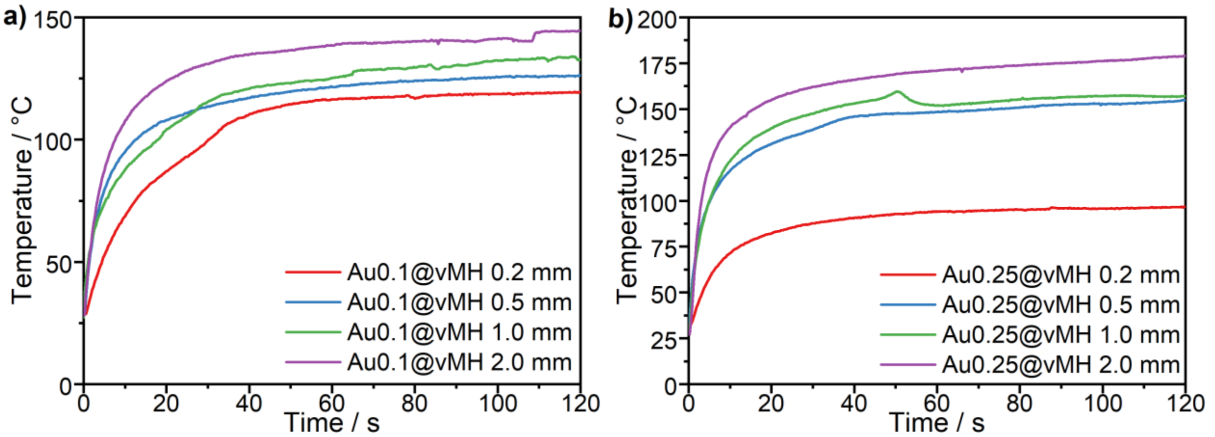

Figure S55: Photothermal heating efficiency. Time-dependent temperature curves of **(a)** Au0.1@vMH films and **(b)** Au0.25@vMH films with different thicknesses, *i.e.*, 0.2, 0.5, 1.0, and 2.0 mm (λ = 532 nm, P =200 mW, e^{-2} spotsize A = 3.2 mm^2, t = 120 s).

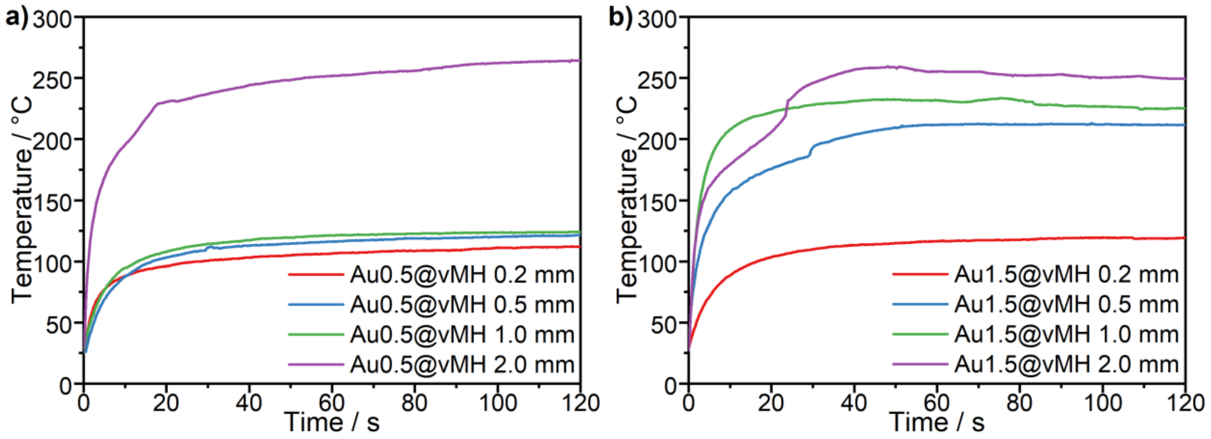

Figure S56: Photothermal heating efficiency. Time-dependent temperature curves of **(a)** Au0.5@vMH films and **(b)** Au1.5@vMH films with different thicknesses, *i.e.*, 0.2, 0.5, 1.0, and 2.0 mm (λ = 532 nm, P =200 mW, e^{-2} spotsize A = 3.2 mm^2, t = 120 s).

Table S9: Parameter of photothermal heating efficiency.

Samples	Thickness [mm]	Intensity [W·cm^{-1}]	$\Delta T_{120\ s}$ [°C]	Initial heating rate [°C s^{-1}]	Initial heating rate [°C J^{-1}]
Au0@vMH	0.2	5.4	69.7	4.1	20.5
	0.5	5.4	66.4	3.7	18.5
	1.0	5.4	71.7	3.8	19.0
	2.0	5.4	65.1	3.0	15.0
Au0.1@vMH	0.2	5.4	91.1	4.1	20.5
	0.5	5.4	98.2	5.0	25.0
	1.0	5.4	105.5	5.4	27.0
	2.0	5.4	116.6	6.6	33.0
Au0.25@vMH	0.2	5.4	66.2	5.9	29.5
	0.5	5.4	126.8	11.5	57.5
	1.0	5.4	128.9	12.8	64.0
	2.0	5.4	152.0	19.8	99.0
Au0.5@vMH	0.2	5.4	87.1	11.3	56.5
	0.5	5.4	95.9	12.3	61.5
	1.0	5.4	94.8	15.0	75.0
	2.0	5.4	234.1	41.1	205.5
	2.0	1.35	28.7	4.8	96.0
	2.0	2.7	68.9	9.5	95.0
	2.0	4.05	151.0	34.4	229.3
Au1.0@vMH	0.2	5.4	108.5	13.5	67.5
	0.5	5.4	163.5	29.3	146.5
	1.0	5.4	194.2	30.0	150.0
	2.0	5.4	230.0	44.7	223.5
Au1.5@vMH	0.2	5.4	90.8	10.4	52.0
	0.5	5.4	183.3	29.6	148.0
	1.0	5.4	198.2	49.6	248.0
	2.0	5.4	222.2	51.3	256.5

References

[1] a) A. Poletti, G. Fracasso, G. Conti, R. Pilot, V. Amendola, *Nanoscale* **2015**, *7*, 13702; b) L. B. Scaffardi, N. Pellegri, O. de Sanctis, J. O. Tocho, *Nanotechnology* **2005**, *16*, 158; c) L. Vigderman, B. P. Khanal, E. R. Zubarev, *Advanced materials (Deerfield Beach, Fla.)* **2012**, *24*, 4811-41, 5014.

[2] S. Zhang, X. Niu, Q. Wang, D. Li, W. Zhang, Y. Chen, F. Ran, *J. Mater. Res.* **2021**, *36*, 925.

[3] C. Li, C. Wang, Z. Ji, N. Jiang, W. Lin, D. Li, *Eur. Polym. J.* **2019**, *113*, 404.

[4] S. Slavin, A. H. Soeriyadi, L. Voorhaar, M. R. Whittaker, C. R. Becer, C. Boyer, T. P. Davis, D. M. Haddleton, *Soft Matter* **2012**, *8*, 118.

[5] D. V. Leff, L. Brandt, J. R. Heath, *Langmuir* **1996**, *12*, 4723.

[6] a) C.-A. Fustin, A.-S. Duwez, *J. Electron. Spectrosc. Relat. Phenom.* **2009**, *172*, 104; b) S. Slavin, A. H. Soeriyadi, L. Voorhaar, M. R. Whittaker, C. R. Becer, C. Boyer, T. P. Davis, D. M. Haddleton, *Soft Matter* **2012**, *8*, 118; c) D. Sahu, H.-C. Chu, P.-J. Yang, H.-C. Lin, *Macro Chemistry & Physics* **2012**, *213*, 1550.

[7] a) W. Li, L. Huo, D. Wang, G. Zeng, S. Xi, B. Zhao, J. Zhu, J. Wang, Y. Shen, Z. Lu, *Colloids Surf., A* **2000**, *175*, 217; b) A.-S. Duwez, *Journal of Electron Spectroscopy and Related Phenomena* **2004**, *134*, 97.

[8] a) S. Won, D. J. Phillips, M. Walker, M. I. Gibson, *J. Mater. Chem. B* **2016**, *4*, 5673; b) B. Kannan, D. A. Higgins, M. M. Collinson, *Langmuir* **2012**, *28*, 16091.
